# ALDOA contributes to colorectal tumorigenesis and metastasis by targeting YAP

**DOI:** 10.1038/s41420-024-02249-z

**Published:** 2025-01-05

**Authors:** Liang Sun, Ting Lu, Linhua Jiang, Huihui Yao, Qixuan Xu, Jie Sun, Xiaoqin Yang, Songbing He, Xinguo Zhu

**Affiliations:** 1 Department of General Surgery, The First Affiliated Hospital of Soochow University, Suzhou, China; 2 Department of Ultrasound, The First Affiliated Hospital of Soochow University, Suzhou, China; 3 School of Basic Medical Sciences, Suzhou Medical College of Soochow University, Suzhou, China

**Keywords:** Cancer metabolism, Gastrointestinal cancer

## Abstract

Metabolic reprogramming is considered one of the hallmarks of cancer in which cancer cells reprogram some of their metabolic cascades, mostly driven by the specific chemical microenvironment in cancer tissues. The altered metabolic pathways are increasingly being considered as potential targets for cancer therapy. In this view, Aldolase A (ALDOA), a key glycolytic enzyme, has been validated as a candidate oncogene in several cancers. The current study aimed to investigate the role of ALDOA in the initiation and development of colorectal cancer (CRC). In this study, we observed an elevated expression of ALDOA in human CRC tissues and a positive correlation of elevated ALDOA expression with tumor size, invasion depth, LNM, and TNM stage. Kaplan–Meier analysis revealed that elevated ALDOA levels correlated with a poor prognosis in CRC patients with stage I-III, whereas the prognosis tends to be favorable in patients with advanced CRC. In addition, loss of function and gain of function experiments showed that ALDOA promoted CRC cell proliferation and migration in vitro and in vivo. Mechanistically, high ALDOA expression inhibited AMP-activated protein kinase (AMPK) phosphorylation possibly through regulating cellular glycolysis or the formation of v-ATPase-regulator-AXIN/LKB1 complex, which led to Yes-associated protein (YAP) unphosphorylation and enhanced the proliferative and migratory potential of CRC cells. Finally, the positive correlation between ALDOA and YAP signaling was also confirmed in clinical CRC tissues and the public data. Herein, ALDOA was identified to be a new metabolic regulator of YAP that suppresses the activation of AMPK signaling. This could suggest a novel avenue for treating CRC by inhibiting both ALDOA and YAP signaling.

## Introduction

Colorectal cancer (CRC) was the fourth-leading cause of cancer death in both men and women younger than 50 years in the late 1990s but is now first in men and second in women [1–4]. As per cancer statistics for 2024, around 152,810 cases of CRC were recorded, with 53,010 deaths expected in the United States in 2024 [4]. Multiple genes and cellular signaling cascades are implicated in the initiation and progression of CRC, according to newly discovered data [5]. Hence, finding new treatment targets and understanding the underlying molecular signaling pathways may enhance the prognostic rate of CRC patients.

Metabolic reprogramming is considered one of the hallmarks of cancer in which cancer cells reprogram their metabolic cascades, mostly driven by the specific chemical microenvironment in cancer tissues [6, 7]. Cancer cells can generate ATP without oxygen, a process described as the Warburg effect [8], which was thought to be at the basis of carcinogenesis [9]. Therefore, high-throughput biochemical techniques have discovered various glycolytic enzymes or metabolic pathways as prospective therapeutic targets for cancer therapy [10, 11].

Fructose-bisphosphate aldolase catalyzes the reversible conversion of fructose-1,6-bisphosphate (FBP) to glyceraldehyde-3-phosphate (G3P) and dihydroxyacetone phosphate (DHAP) in the glycolytic pathway [12, 13]. Aldolase A (ALDOA) is one of three aldolase isozymes (A, B, and C) involved in cellular functions, such as ATP generation [14–16]. ALDOA has been revealed to be highly expressed in numerous malignancies, including human pancreatic [16, 17], prostate [18], hepatocellular [19, 20], cervical [21], bladder [22], gastric [23], and lung cancer [24–26]. In CRC, relative to healthy glandular epithelium tissues, ALDOA was found to be substantially expressed in tumor tissues and liver metastatic CRC [27], and an elevated level of ALDOA contributed to the poor survival outcomes in CRC patients [27, 28]. However, the exact role and mechanism of ALDOA in CRC have not been fully explored.

The reported studies have revealed that the Hippo cascade has a key contribution to tumor suppression and the alteration in this cascade leading to the initiation of malignancies [29, 30]. The mammalian STE20-like protein kinase (MST1/2) and the large tumor suppressor (LATS1/2) are two of the most important inhibitory kinases in mammalian systems. While Yes-associated protein (YAP) and PDZ-binding motif (TAZ) are transcriptional co-activators that are comprised of the transcriptional module. The phosphorylation of YAP/TAZ takes place when the upstream kinase module is stimulated, resulting in 14-3-3-mediated cytoplasmic retention and proteasomal destruction of YAP and TAZ mediated by ubiquitin [31]. To activate its target genes, the translocation of unphosphorylated YAP/TAZ occurs into the nucleus and then interacts with the TEAD family of transcription factors (such as *CYR61* and *AREG*). Aside from the Hippo/LATS cascade, cellular energy stress can also regulate YAP activity via AMP-activated protein kinase (AMPK) [32–34].

In this study, ALDOA expression was substantially elevated in CRC tissues and was correlated with prognosis of CRC patients. The loss and gain of function investigations revealed that ALDOA elevated the proliferative and migratory potential of CRC cells in vitro and in vivo. High ALDOA expression leads to AMPK inhibition and YAP unphosphorylation. Unphophorylated YAP translocates into the nucleus and triggers its target genes expression that promotes CRC cell proliferation and migration. Our findings reveal that ALDOA can function as a crucial regulator of YAP and may become a novel therapeutic target for CRC.

## Results

### ALDOA is upregulated in human patients with CRC

**Fig. 1:**
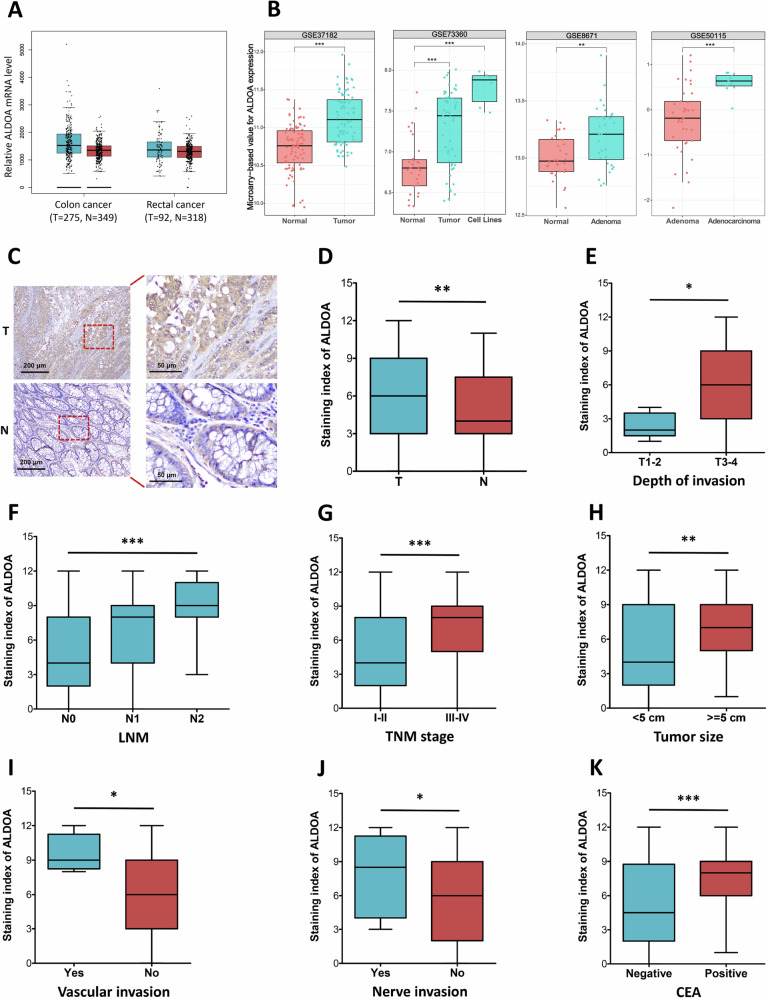
ALDOA expression in CRC tissues. **A** ALDOA gene expression in CRC derived from the GEPIA database. T: tumor tissues, N: normal tissues. **B** ALDOA gene expression in CRC tissues and cell lines derived from four published microarray datasets (GSE37182, GSE73360, GSE8671 and GSE50115). **C** Typical images of ALDOA IHC staining in CRC tumor tissues and surrounding normal tissues. **D** The IHC scores of ALDOA in CRC tissues (T) and normal tissues (N). IHC staining to analyze ALDOA expression in CRC tissues of different depth of invasion (**E**, T1-2 vs. T3-4), LNM (**F**, N1 vs. N2 vs. N3), TNM stage (**G**, I–II vs. III–IV), tumor size (**H**, < 5 cm vs. ≥ 5 cm), Vascular invasion (**I**, Yes vs. No), Nerve invasion (**J**, yes vs. no) and serum CEA level (**K**, negative vs. positive). *P < 0.05, **P < 0.01, ***P < 0.001.

To evaluate the role of ALDOA in CRC, the ALDOA expression level was analyzed via the public GEPIA database and GEO databases. According to the obtained results, the ALDOA mRNA level was found to be elevated in CRC tumor tissues and cancer cells, as compared to the normal tissues (Fig. 1A, B, Supplementary Fig. S1A). Additionally, the public data showed that ALDOA mRNA was upregulated in colorectal adenoma (*P* < 0.01, Fig. 1B), and was further elevated in colorectal adenocarcinoma (*P* < 0.001, Fig. 1B), suggesting the progressive elevation of ALDOA expression from adenoma to adenocarcinoma. Consistent with public data, IHC staining demonstrated an enhanced amount of ALDOA at the protein level in CRC tumor tissue samples (*P* < 0.01, Fig. 1C, D). Further analysis showed that ALDOA protein levels in CRC tumor tissues with deeper invasion (T3-4) and positive lymph node metastasis (LNM) were significantly higher compared with tissues with T1-2 and negative LNM, respectively (Fig. 1E, F). Since the tumor-node-metastasis (TNM) stage closely correlates to tumor malignancy, we investigated the correlation between the expression level of ALDOA and different stages of TNM (*i.e*., stage I, II, III, and IV). As expected, the results showed a positive correlation between ALDOA expression and the TNM stage (*P* < 0.001, Fig. 1G). Besides, our comparative analysis revealed that CRC tumor tissues with larger tumor size (*P* < 0.01, Fig. 1H), vascular invasion (*P* < 0.05, Fig. 1I), nerve invasion (*P* < 0.05, Fig. 1J) and positive serum CEA level (*P* < 0.001, Fig. 1K) showed higher ALDOA protein levels compared with their control counterparts, respectively. However, the expression of the ALDOA protein did not correlate with other factors, such as age, gender, tumor location, degree of differentiation, tumor gross classification, microsatellite stabilization or the serum CA199 level (*P* > 0.05, Supplementary Fig. S1B–H).

The correlation between ALDOA expression levels and clinicopathological characteristics was investigated using the Chi-square test (Supplementary Table S1). Notably, ALDOA expression was considerably correlated with tumor size, invasion depth, LNM, and TNM stage (*P* = 0.001, 0.009, 0.001, and < 0.001, respectively). However, no link was found between ALDOA expression and other clinicopathological characteristics, such as age, gender, tumor location, gross classification, degree of differentiation, vascular invasion, nerve invasion or microsatellite stabilization (*P* > 0.05, Supplementary Table S1).

### ALDOA expression is correlated with survival of CRC patients

**Fig. 2:**
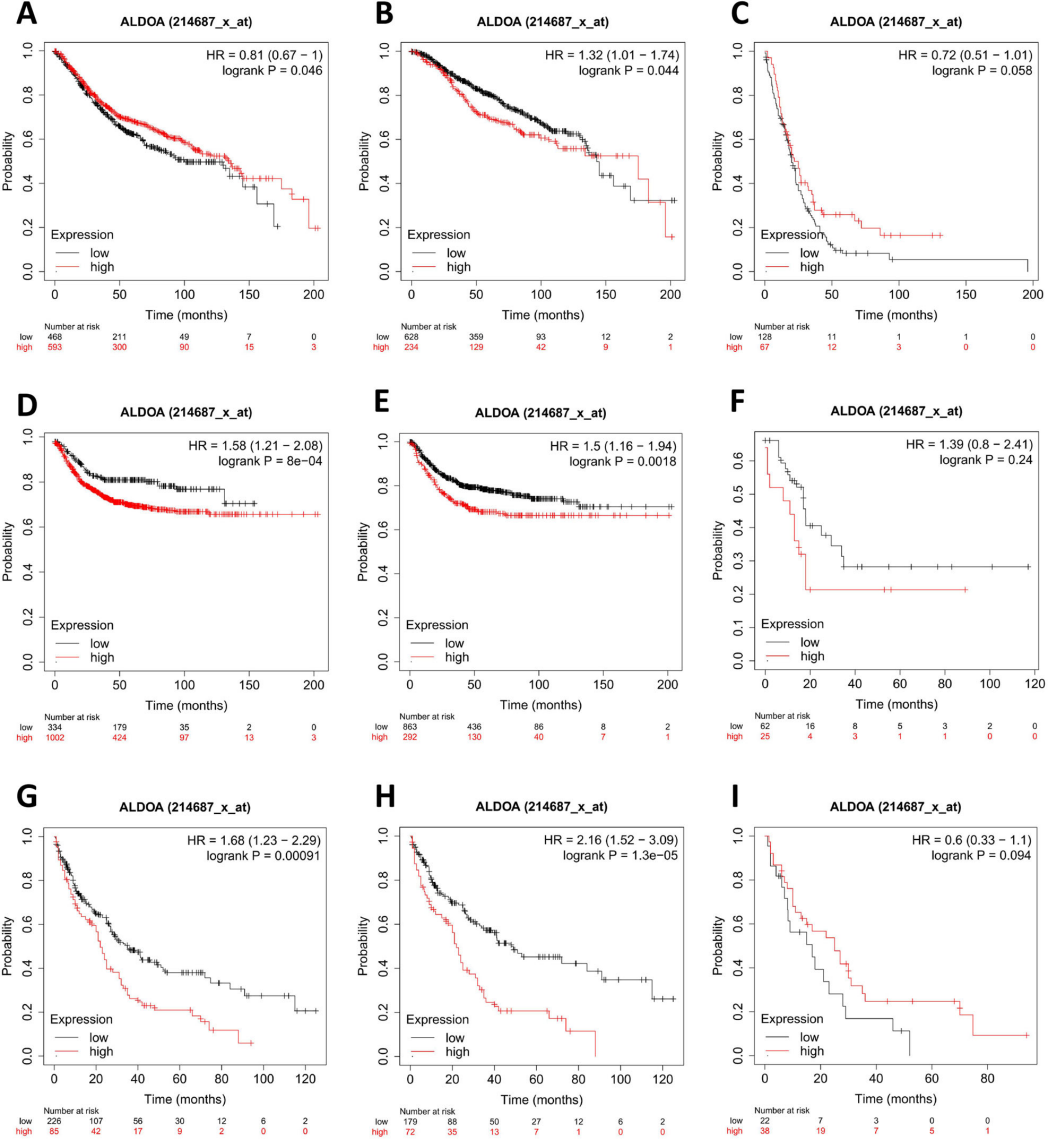
ALDOA mRNA expression is correlated with survival of CRC patients from the Kaplan–Meier plotter databases. **A** Kaplan–Meier curve for the overal survival (OS) of CRC patients. Kaplan–Meier curve for the OS of CRC patients with different tumor staging (stage I–III (**B**), stage IV (**C**)). **D** Kaplan–Meier curve for the relapse-free survival (RFS) of CRC patients. Kaplan–Meier curve for the RFS of CRC patients with different tumor staging (stage I-III (**E**), stage IV (**F**)). **G** Kaplan–Meier curve for the post-progression survival (PPS) of CRC patients. Kaplan–Meier curve for the PPS of CRC patients with different tumor staging (stage I–III (**H**), stage IV (**I**)).

To elucidate the correlation between ALDOA expression with CRC patient’s prognosis, Kaplan–Meier curves for the overal survival (OS), relapse-free survival (RFS) and post-progression survival (PPS) of CRC patients were obtained from Kaplan–Meier plotter databases. The results indicated that the high-expression cohort had a slightly better OS than the low-expression cohort in patients with all TNM stages (HR = 0.81, *P* = 0.046, Fig. 2A), whereas there was an opposite trend in stage I–III group (HR = 1.32, *P* = 0.044, Fig. 2B). In stage IV group, patients with high ALDOA expression showed a better OS than the low cohort (HR = 0.72, *P* = 0.058, not significant, but marginal, Fig. 2C), which may explain the above contradictory finding. Besides, patients with high ALDOA expression had a remarkably shorter RFS than the low group (HR = 1.58, *P* = 8e-04, Fig. 2D), and a same trend was observed both in patients with stage I–III (HR = 1.5, *P* = 0.0018, Fig. 2E) and stage IV (HR = 1.39, *P* = 0.24, Fig. 2F). Moreover, results showed that the high-expression cohort had shorter PPS than the low-expression cohort both in patients with all stages (HR = 1.68, *P* = 0.00091, Fig. 2G) and in patients with stage I–III (HR = 2.16, *P* = 1.3e-05, Fig. 2H). However, an opposite trend was observed in stage IV group (HR = 0.6, *P* = 0.094, Fig. 2I). In addition, Kaplan–Meier curves for the OS, RFS and PPS of CRC patients with different tumor staging were showed in the Supplementary data (Supplementary Figs. S2, S3 and S4). Collectively, these results indicate that elevated ALDOA levels correlate with a poor prognosis in CRC patients with stage I-III, while the prognosis tends to be favorable in patients with advanced CRC.

### ALDOA expression in human CRC cell lines

The ALDOA expression in five CRC cell lines (HT29, CaCo2, DLD-1, SW480, SW620) was assessed. The results revealed an elevated ALDOA expression in HT29, CaCo2, and DLD-1 cells at both transcriptional (mRNA), and translational (protein) levels relative to the expression level of ALDOA in SW480 and SW620 cells (Supplementary Fig. S5A, B). Using a lentivirus-based technique, we modified the CRC cell lines with either stably silenced ALDOA or stably overexpressed ALDOA. HT29 and DLD-1 cells, which express relatively high endogenous ALDOA, were utilized for gene silencing. According to the results, ALDOA-targeting shRNA (KD) transfected HT29 and DLD-1 cells exhibited a significantly lower expression of ALDOA (at both transcriptional and translational levels) when compared to the control-shRNA group (NC, Supplementary Fig. S5C, D). Similarly, SW480 and SW620 cells (with relatively lower endogenous ALDOA expression) were used for ectopic gene expression. The results showed that the expression of ALDOA (both transcriptional and translational levels) in Lenti-ALDOA (OE) transfected cells (i.e., SW480 and SW620) was significantly increased when compared to the empty vector group (VEC, Supplementary Fig. S5E, F).

### ALDOA promotes the proliferative and migratory capabilities of CRC cells in vitro and in vivo

**Fig. 3:**
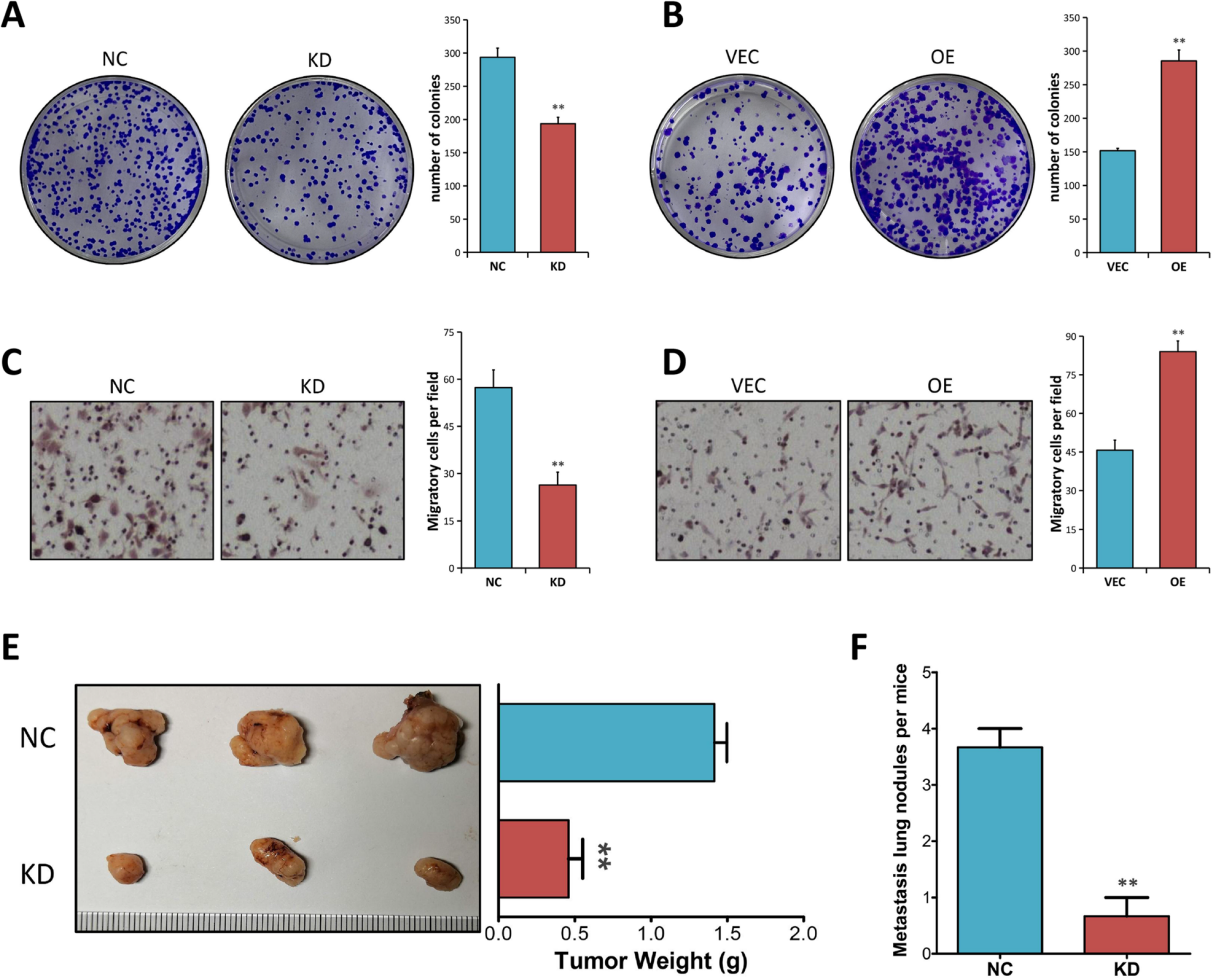
ALDOA promotes the proliferation and migration of CRC cells in vitro and in vivo. **A**, **B** The cell proliferation ability was examined by colony formation assays in HT-29 cells (NC vs. KD) and in SW480 cells (VEC vs. OE). **C**, **D** The migration capacity was evaluated by Transwell assays in HT-29 cells (NC vs. KD) and in SW480 cells (VEC vs. OE). **E** Representative images of xenograft tumors derived from HT-29 cells (NC vs. KD) in nude mice. The weight of tumors in different groups was presented (right). **F** Number of metastatic lung nodules was presented. **P < 0.01.

Considering the clinical importance of ALDOA in CRC, we next carried out the colony formation assay to study the biological roles of ALDOA in CRC. The results indicated that when cells were depleted of ALDOA, their clonogenicity was significantly reduced in HT29 and DLD-1 cells (*P* < 0.01, Fig. 3A and Supplementary Fig. S6A), whereas the ability of SW480 cells to form foci was significantly enhanced when ALDOA was overexpressed (*P* < 0.01, Fig. 3B). According to the transwell migration assays, ALDOA knockdown impaired the migratory capabilities of HT29 and DLD-1 cells (*P* < 0.01, Fig. 3C and Supplementary Fig. S6B). On the other hand, ectopic ALDOA promoted the migratory capabilities of SW480 cells (*P* < 0.01, Fig. 3D).

The in vivo effect of ALDOA was next investigated using two mouse models based on subcutaneous and tail intravenous injections of HT29 cells. Consistent with the in vitro data, xenografts with stable ALDOA knockdown grew at a slower rate and had a significantly lower tumor weight than the control group (*P* < 0.01, Fig. 3E). Furthermore, mice injected with ALDOA-suppressed HT29 cells had a reduced level of lung metastatic nodules than the control group (*P* < 0.01, Fig. 3F).

### ALDOA modulates YAP activity in CRC

**Fig. 4:**
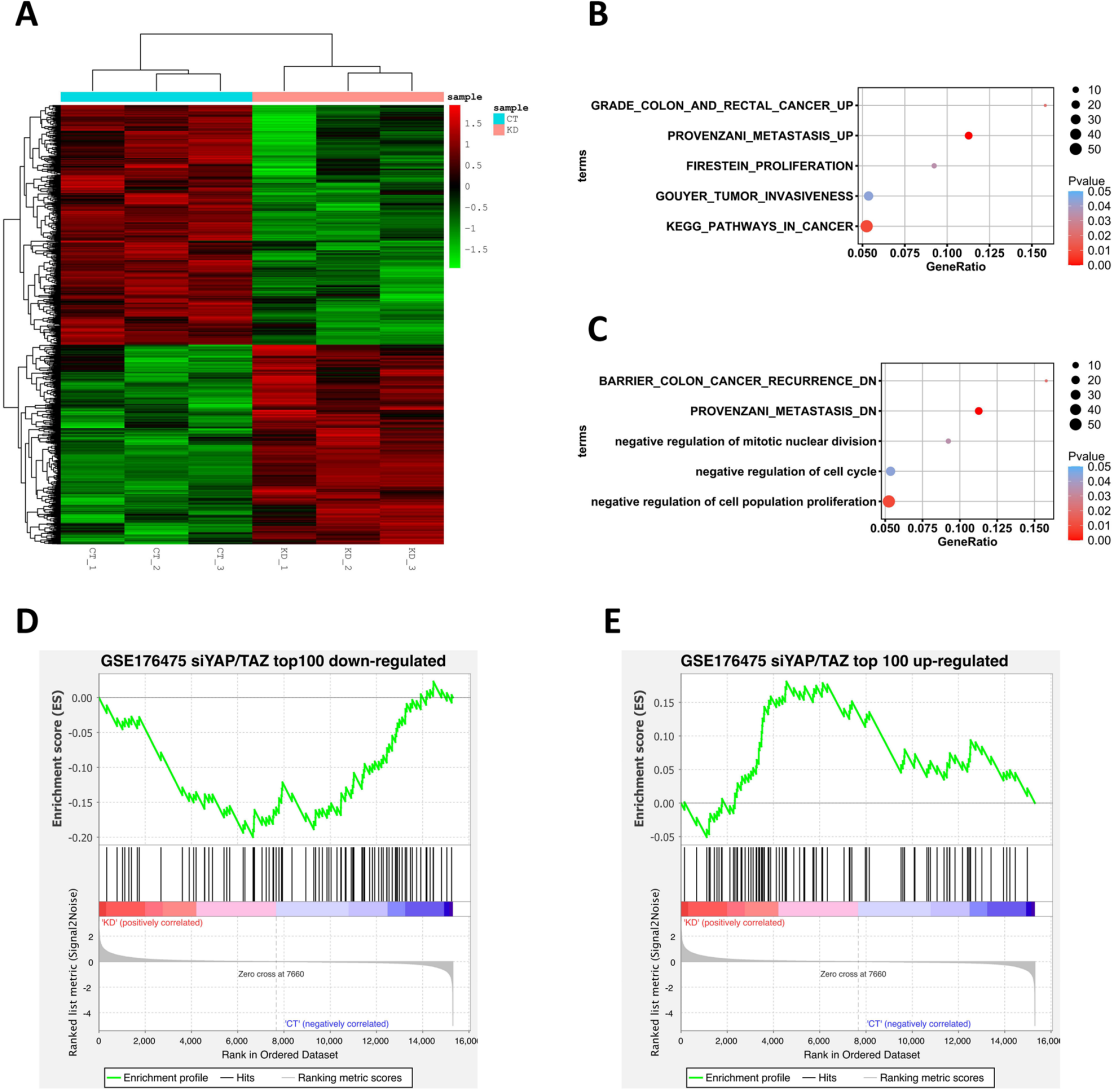
RNA-Seq experiment in HT29 cells. **A** Heatmap for differentially expressed genes (ALDOA KD vs Control) identified by the RNA-Seq assay. Functional enrichment for the up-regulated genes (**B**) and down-regulated genes (**C**). Gene set enrichment analysis (GSEA) for the RNA-Seq expression profile. The positive (**D**) and negative (**E**) signatures of the YAP signaling pathway in colon cancer cells were defined by the top 100 down-regulated genes and top up-regulated genes identified by the GSE176475 Transcriptomics dataset (siYAP/TAZ KD vs Control).

To decipher the molecular mechanism underlying ALDOA expression, we performed the RNA-Seq experiment on HT29 cells. Totally, 862 up-regulated genes and 1036 down-regulated genes were identified (Fig. 4A). Following functional enrichment analyses for the differentially expressed genes were performed to investigate the potential biological processes in colon cancer influenced by the ALDOA gene. Down-regulated genes (Fig. 4B) enriched genes highly expressed in colon and rectal cancers, pathways in cancer, and processes including metastasis, proliferation, and invasiveness in colon cancers. For the up-regulated counterpart (Fig. 4C), gene sets negatively associated with colon cancer recurrence and metastasis and negatively regulating mitotic nuclear division, cell cycle, and cell population proliferation were significantly overrepresented. Interestingly, further GSEA analysis revealed that the positive and negative signatures of YAP signaling cascade in colon cancer cells were significantly enriched (Fig. 4D, E, P were 0.002 and 0.006, respectively), which suggested the potential link between the ALDOA gene and this pathway.

**Fig. 5:**
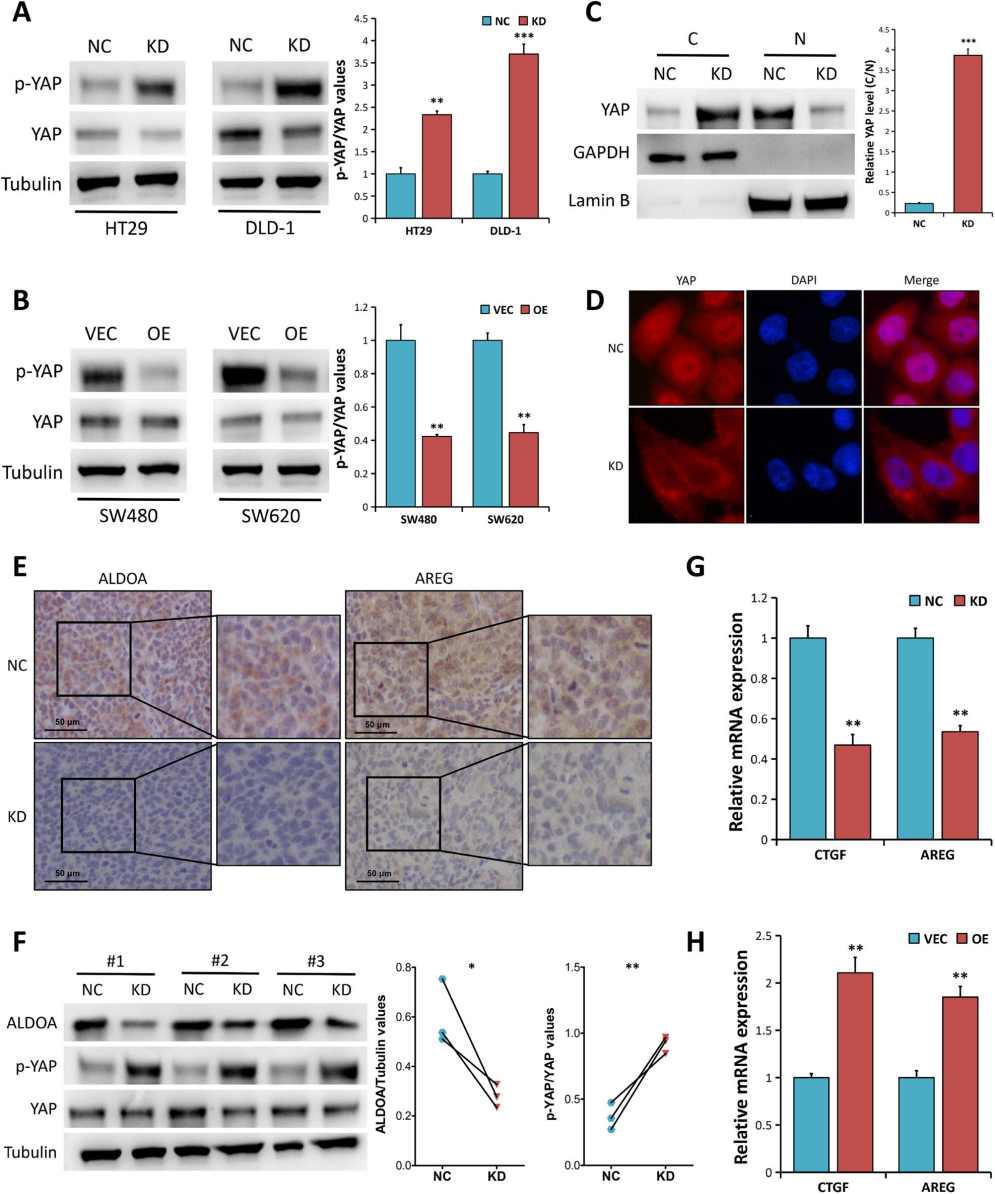
ALDOA modulates YAP activity. **A** Western blotting analysis of p-YAP and YAP expression in HT-29 and DLD-1 cells (NC vs. KD). **B** Western blotting analysis of p-YAP and YAP expression in SW480 and SW620 cells (VEC vs. OE). **C** Subcellular fractionation analysis of YAP expression in HT-29 cells (NC vs. KD). GAPDH served as control for the cytoplasmic (C) fraction, and Lamin B served as control for the nuclear (N) fractions. **D** IF staining of YAP (red) in HT-29 cells (NC vs. KD). Nuclei were stained with DAPI (blue). **E** Typical images of IHC staining of ALDOA and AREG in the xenograft tumors derived from HT-29 cells (NC vs. KD). **F** Western blotting analysis of the indicated proteins in the xenograft tumors derived from HT-29 cells (NC vs. KD). (**G** qRT-PCR analysis of CTGF and AREG mRNA levels in HT-29 cells (NC vs. KD). **H** qRT-PCR analysis of CTGF and AREG mRNA levels in SW480 cells (VEC vs. OE). *P < 0.05, **P < 0.01, ***P < 0.001.

Additionally, overexpression of YAP has been shown to promote CRC development [35, 36], and cellular energy metabolism is implicated in YAP activity regulation [32–34]. Therefore, we investigated whether ALDOA is involved in the regulation of YAP activity. Results from Western blot analysis (Fig. 5A) showed a significant increase of YAP phosphorylation in ALDOA-depleted HT29 (*P* < 0.01) and DLD-1 cells (*P* < 0.001). Moreover, ALDOA knockdown had no significant effect on the total YAP protein expression. In contrast, ALDOA elevation inhibited YAP phosphorylation in SW480 and SW620 cells, with no effect on the total YAP protein expression (*P* < 0.01, Fig. 5B). To investigate whether ALDOA regulates YAP through LATS1-dependent pathway, we used Western blot analysis to test LATS1 phosphorylation by ALDOA genetic manipulation in CRC cells. Our results revealed that ALDOA knockdown or overexpression had minimal effect on LATS1 and p-LATS1 protein levels (Supplementary Fig. S7), indicating there might be other molecules or signaling involved in the regulation of YAP expression by ALDOA in CRC cells. It has been validated that YAP protein in the nucleus exists in the active form [31], we aimed to examine the effect of ALDOA on the subcellular distribution of YAP. The results indicated that ALDOA-depleted HT29 cells had decreased nuclear and elevated cytoplasmic YAP levels (*P* < 0.001, Fig. 5C). Consistent with the Western blot analysis, Immunofluorescence staining showed that ALDOA depletion prevented YAP translocation from cytoplasm to nucleus (Fig. 5D).

To validate the impact of ALDOA on YAP activity, we subsequently examined the expression of AREG (a typical YAP target gene) protein in xenografted tumors using IHC staining. IHC staining revealed a significant reduction in AREG protein expression in tumors derived from ALDOA-depleted HT29 cells when compared to the control group (Fig. 5E), revealing a positive correlation between ALDOA and AREG in protein expression. Similarly, in vivo studies showed that ALDOA expression was inversely correlated with YAP phosphorylation in HT-29-derived xenograft tumors (Fig. 5F). Since nuclear YAP functions as a transcriptional co-activator, the mRNA expression levels of typical YAP target genes (*CTGF* and *AREG*) were detected by qRT-PCR. The results revealed that silencing ALDOA expression inhibited the expression of CYR61 and AREG mRNA in HT29 cells (*P* < 0.01, Fig. 5G), whereas, overexpression of ALDOA promoted their expression in SW480 cells (*P* < 0.01, Fig. 5H).

### CRC cell proliferative and migratory capabilities are promoted by ALDOA in a YAP-dependent manner

**Fig. 6:**
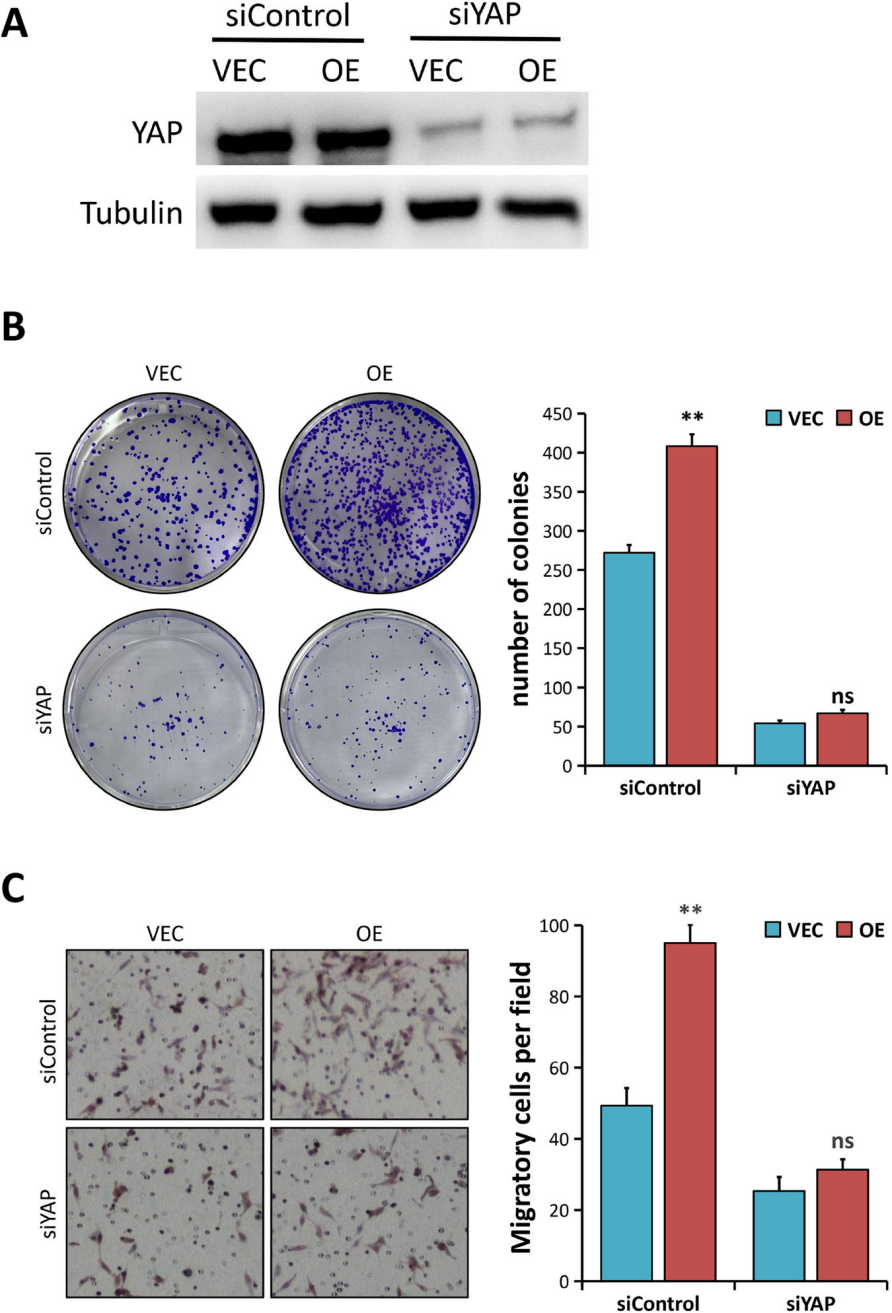
ALDOA promotes CRC cell proliferation and migration in a YAP-dependent manner. **A** Western blotting analysis of YAP expression in SW480 cells (VEC vs. OE) treated with siRNA against YAP (siYAP) and siControl. **B** The cell proliferation ability was examined by colony formation assays in SW480 cells (VEC vs. OE) treated with siYAP and siControl. **C** The migration capacity was evaluated by Transwell assays in SW480 cells (VEC vs. OE) treated with siYAP and siControl. ns, nonsignificant, **P < 0.01.

We used two siRNAs targeting YAP to knock down YAP expression in SW480 cells with or without ALDOA overexpression to detect whether YAP is involved in the oncogenesis of ALDOA in CRC. The results obtained from Western blotting showed that the YAP protein expression was suppressed in cells treated with siYAP, compared to the siControl group (Fig. 6A and Supplementary Fig. S8A). Ectopic ALDOA significantly enhanced the proliferative and migratory capabilities of SW480 cells in colony formation and transwell migration assays (*P* < 0.01, Fig. 6B, C, Supplementary Fig. S8B, C). However, ALDOA overexpression failed to enhance cell proliferation and migration in YAP-depleted SW480 cells (*P* > 0.05, Fig. 6B, C, Supplementary Fig. S8B, C). Given the underlined results, ALDOA’s oncogenic properties in CRC are YAP-dependent.

### ALDOA regulates YAP activity through the AMPK pathway in CRC cells

**Fig. 7:**
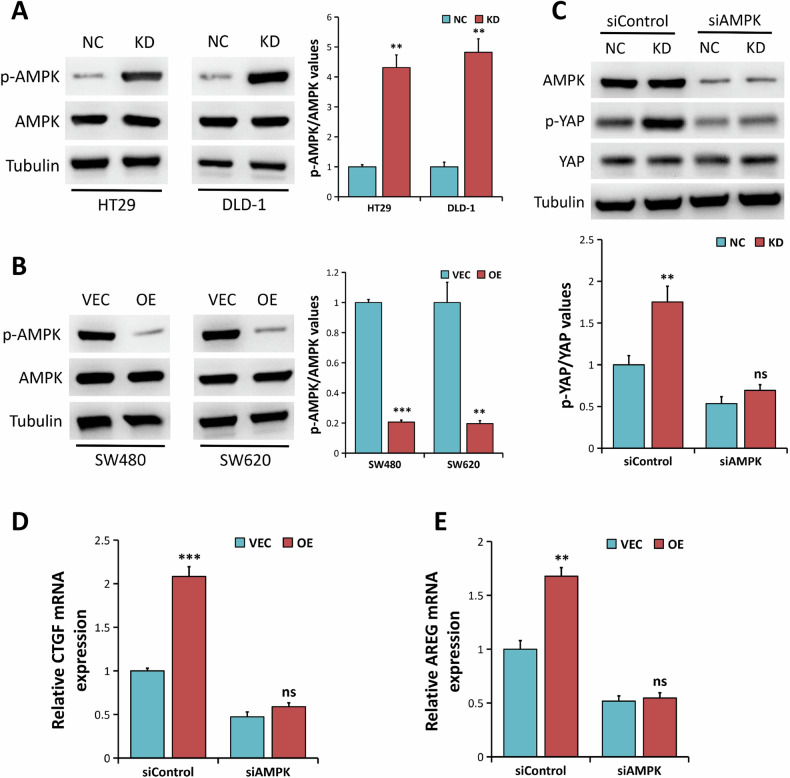
ALDOA regulates YAP activity through AMPK pathway. **A** Western blot analysis of p-AMPK and AMPK expression in HT-29 and DLD-1 cells (NC vs. KD). **B** Western blot analysis of p-AMPK and AMPK expression in SW480 and SW620 cells (VEC vs. OE). **C** Western blotting analysis of the indicated proteins in HT-29 cells (NC vs. KD) treated with siRNA against AMPK (siAMPK) and siControl. **D** qRT-PCR analysis of CTGF mRNA expression in SW480 cells (VEC vs. OE) treated with siAMPK and siControl. **E** qRT-PCR analysis of AREG mRNA expression in SW480 cells (VEC vs. OE) treated with siAMPK and siControl. ns, nonsignificant, **P < 0.01, ***P < 0.001.

ALDOA has been shown to inhibit AMPK activation in a variety of cell types [37–40], and cellular energy depletion induces AMPK-mediated regulation of YAP [32–34]. Therefore, we speculated that AMPK might be implicated in ALDOA-mediated YAP activation. Western blot analysis was used to investigate the effect of ALDOA on AMPK phosphorylation in CRC cells. The results showed a significant increase of AMPK phosphorylation in ALDOA-depleted HT29 and DLD-1 cells (*P* < 0.01, Fig. 7A). On the other hand, ectopic ALDOA inhibited AMPK phosphorylation in SW480 and SW620 cells while not affecting total AMPK protein expression (Fig. 7B), implying that ALDOA inhibited AMPK activation in CRC cells.

To investigate whether AMPK is involved in the ALDOA activation of YAP, we utilized two AMPK-specific siRNAs (siAMPK) to knock down AMPK expression in HT29 cells with or without ALDOA depletion. Western blot analysis revealed that siAMPK significantly inhibited AMPK protein expression in cells transfected with siAMPK, compared to the siControl group (Fig. 7C and Supplementary Fig. S9). Moreover, we found that ALDOA knockdown significantly promoted the phosphorylation of YAP in siControl-treated HT29 cells (*P* < 0.01, Fig. 7C and Supplementary Fig. S9). However, ALDOA depletion could not phosphorylate YAP when AMPK was silenced (*P* > 0.05, Fig. 7C and Supplementary Fig. S9). Hence, the underlined results suggest that AMPK plays a key role in the ALDOA associated regulation of YAP. We then used qRT-PCR to determine the mRNA expression levels of YAP target genes (*CTGF* and *AREG*). The results indicated that ectopic ALDOA expression increased the expression of CTGF and CYR61 mRNA in siControl-treated SW480 cells (Fig. 7D, E). However, when AMPK was depleted, the increased levels of CTGF and CYR61 mRNA expression induced by ALDOA overexpression were reversed (*P* > 0.05, Fig. 7D, E), indicating that AMPK is involved in ALDOA-mediated YAP activation.

### ALDOA expression is positively correlated with CTGF and AREG expression in clinical CRC tissues

**Fig. 8:**
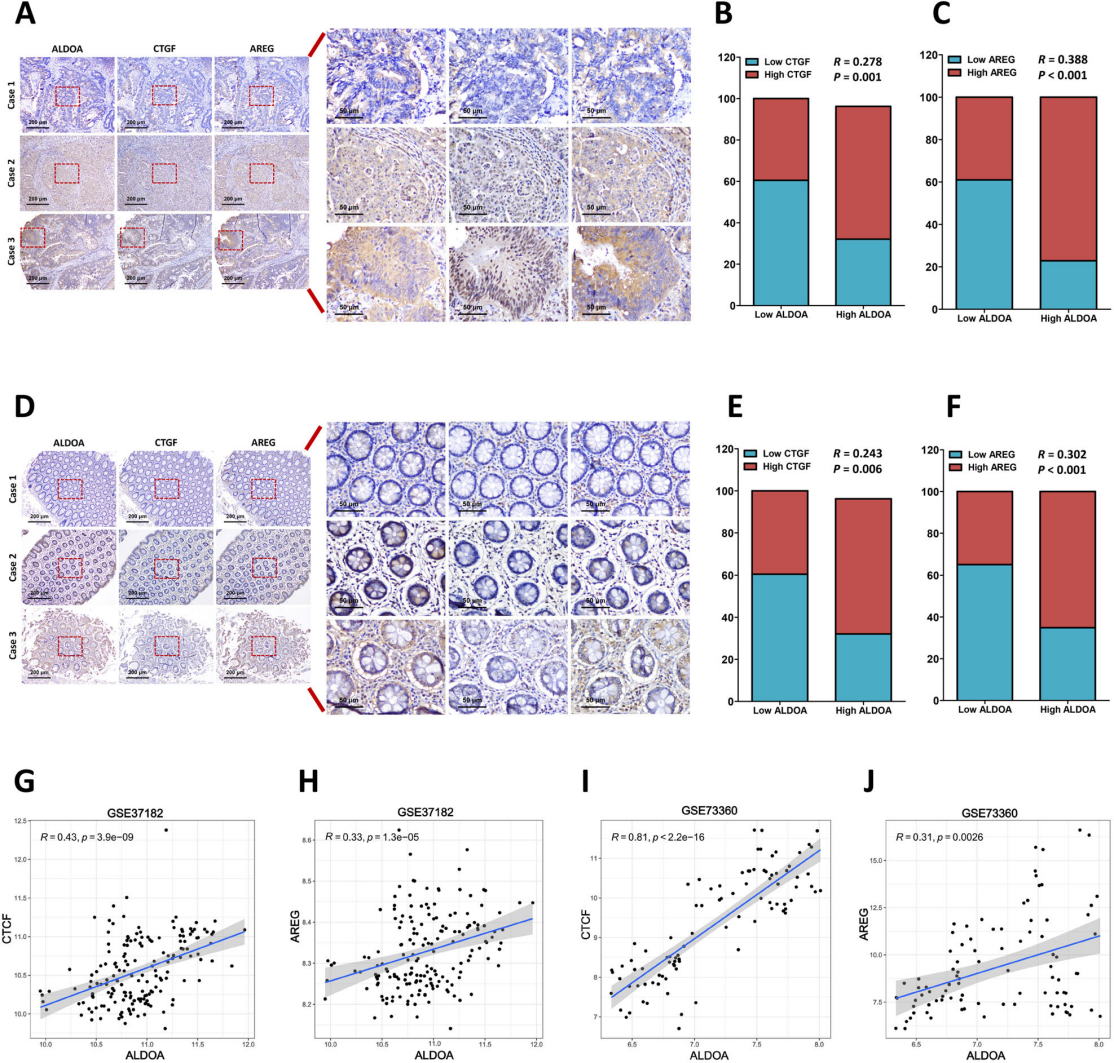
The expression levels of ALDOA, CTGF and AREG in clinical CRC tissues. **A** IHC staining of ALDOA, CTGF and AREG in CRC tumor tissues. Spearman correlation analysis between ALDOA and CTGF (**B**), and ALDOA and AREG (**C**) in CRC tumor tissues. **D** IHC staining of ALDOA, CTGF and AREG in normal tissues. Spearman correlation analysis between ALDOA and CTGF (**E**), and ALDOA and AREG (**F**) in normal tissues. **G** Correlation analysis of ALDOA and CTGF gene expression based on the published microarray dataset (GSE37182). **H** Correlation analysis of ALDOA and AREG gene expression based on the published microarray dataset (GSE37182). **I** Correlation analysis of ALDOA and CTGF gene expression based on the published microarray dataset (GSE73360). **J** Correlation analysis of ALDOA and AREG gene expression based on the published microarray dataset (GSE73360).

To further confirm ALDOA regulation of YAP, the correlation between ALDOA expression with the critical molecules downstream of YAP signaling (CTGF and AREG) expression was analyzed via clinical specimens and the public GEO databases. Our results from IHC analysis showed ALDOA expression was positively correlated with CTGF and AREG expression in CRC tumor tissues (Fig. 8A–C). Similarly, a positive correlation between ALDOA with CTGF and AREG was also observed in normal colorectal mucosal tissue (Fig. 8D–F). To further confirm the correlation, we detected the mRNA expression in the published GEO databases. Consistent with the IHC analysis, the public data further confirmed the positive correlation between ALDOA mRNA expression with CTGF and AREG mRNA expression in GSE37182 and GSE73360 datasets (Fig. 8G–J). These data indicate a potential role for ALDOA in regulating YAP activity in human CRC tissues.

### ALDOA regulates cellular glycolysis and ATP production in CRC cells

**Fig. 9:**
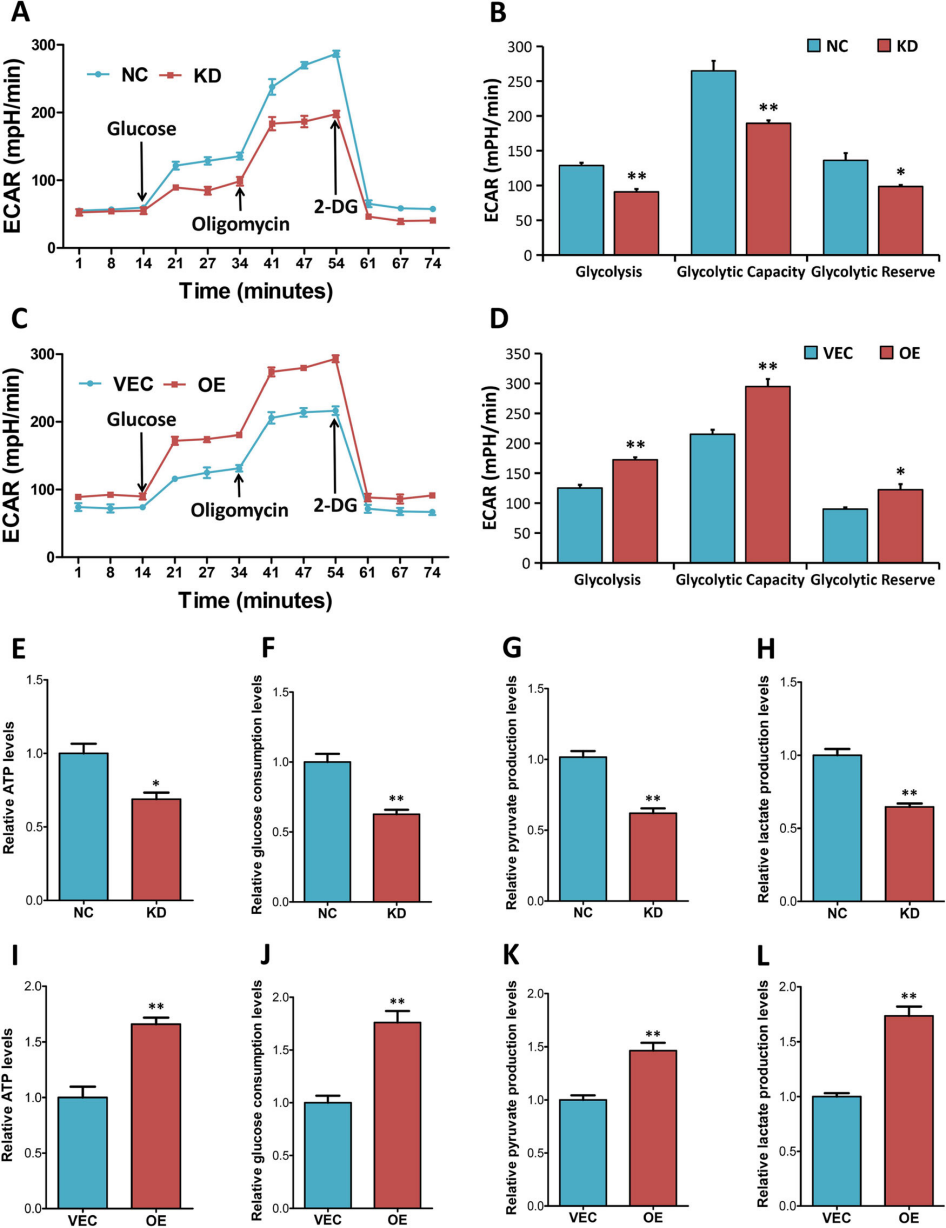
ALDOA regulates cellular glycolysis and ATP production in CRC cells. **A**, **B** Knockdown of ALDOA effectively decreased ECAR in HT29 cells. **C**, **D** ALDOA overexpression significantly increased ECAR in SW480 cells. Determination of ATP level (**E**, **F**), glucose consumption (**G**, **H**), pyruvate production (**I**, **J**) and lactate production (**K**, **L**) in HT-29 cells (NC vs. KD) and SW480 cells (VEC vs. OE). *P < 0.05, **P < 0.01.

**Fig. 10:**
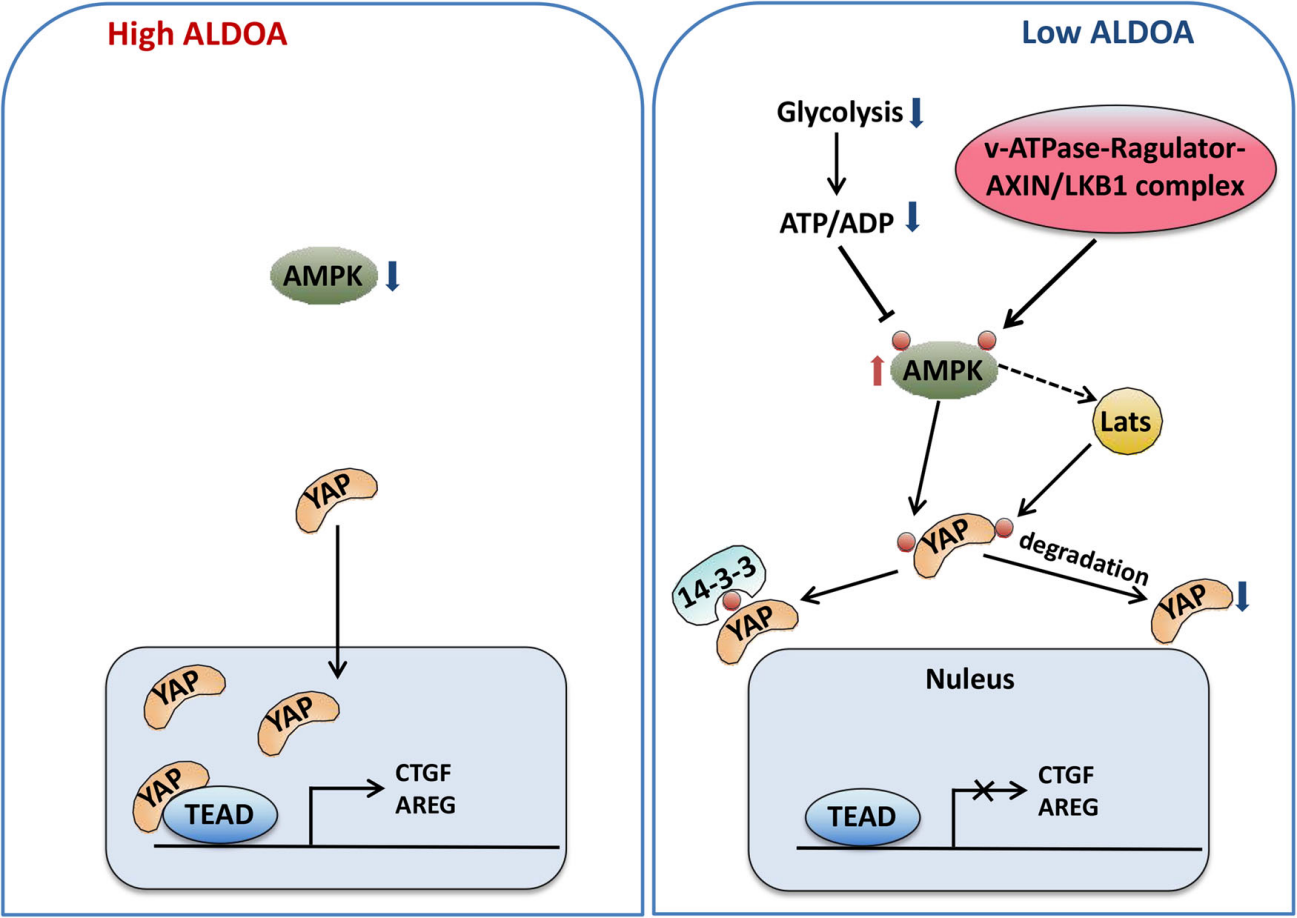
Schematic diagram of the molecular mechanism of ALDOA regulation of AMPK/YAP pathway. High ALDOA expression leads to AMPK inhibition and YAP unphosphorylation. Unphophorylated YAP translocates into the nucleus and functions as a transcriptional co-activator by binding to the TEAD family of transcription factors. The YAP-TEAD complex triggers its target genes (*CTGF* and *AREG*) expression that promote cell proliferation and migration. Under low expression of ALDOA, cellular ATP formation is decreased due to low glycolysis. A decreased ratio of ATP/ADP leads to phosphorylation of AMPK. Knockdown of ALDOA can also trigger the formation of v-ATPase-Ragulator-AXIN/LKB1 complex, which activates AMPK in an AMP/ADP-independent manner. Activated AMPK then phosphorylates YAP, promoting its binding to 14-3-3, thus resulting in its cytoplasmic localization and functional inactivation. Moreover, phosphorylated YAP is also targeted for ubiquitylation and degradation.

Since ALDOA is a key enzyme in the glucose metabolic pathway, we wondered whether ALDOA affected glycolysis in CRC cells. Our results from seahorse extracellular flux analysis showed that ALDOA depletion significantly inhibited basic glycolytic capacity (*P* < 0.01), maximum glycolytic capacity (*P* < 0.01) and glycolytic reserve (*P* < 0.05, Fig. 9A, B), while ectopic ALDOA did the opposite in SW480 cells (Fig. 9C, D). Metabolic product detection revealed that ALDOA silence reduced cellular ATP level (Fig. 9E), glucose uptake (Fig. 9F), pyruvate production (Fig. 9G) and lactate production (Fig. 9H) in HT29 cells, while ALDOA overexpression exerted the opposite effects in SW480 cells (Fig. 9I–L). Collectively, our results indicate that ALDOA regulates the glycolysis pathway in CRC cells. In summary, we reveal ALDOA as a novel metabolic regulator of YAP via inhibiting the AMPK signaling cascade (Fig. 10).

## Discussion

Tumor cells are actively involved in aerobic glycolysis to fuel their unrestrained proliferation and migration; as metabolic reprogramming is thought to be a distinguishing feature of carcinoma [6–9]. Therefore, metabolic manipulation, such as targeting some glycolytic enzymes or metabolic pathways, may provide a strategy for cancer treatment. FBP is converted to G3P and DHAP by ALDOA, which is considered to be a major enzyme in the glycolytic pathway [12, 13]. In addition to its glycolytic function, ALDOA has been validated as a candidate oncogenic protein in a variety of cancers [16–26]. In CRC tissues, ALDOA was found to be with elevated expression [27], which is positively correlated with the aggressiveness and low survival outcome of CRC patients [27, 28]. However, the precise function and underlying mechanisms of ALDOA in CRC are not fully explored and require further investigation.

In the current study, we demonstrated that ALDOA expression was increased in human CRC tissues. Additionally, clinical evaluation demonstrated a positive correlation between high ALDOA expression and tumor size, invasion depth, LNM, and TNM stage. K-M analysis revealed that elevated ALDOA levels correlated with a poor prognosis in CRC patients with stage I-III, whereas the prognosis tends to be favorable in patients with advanced CRC. Furthermore, the loss and gain of function assay suggested that ALDOA depletion inhibited CRC cell proliferation and migration while ectopic ALDOA showed the opposing results, indicating the oncogenic roles of ALDOA in CRC.

ALDOA exerts oncogenic activity in cancers via different signaling pathways. The function of ALDOA in pancreatic cancer [16] could be attributed to its regulatory role of HIF1α and c-Myc. In lung cancer, ALDOA exhibits a tumor-promoting effect through the EGFR/MAPK signaling cascade and modulation of HIF-1α signaling [24, 25]. Moreover, it may interact with γ-actin to promote lung cancer metastasis [26]. Finally, ALDOA increases malignant potentials by inducing the epithelial-mesenchymal transition (EMT) progress [22]. Herein, we demonstrated for the first time that ALDOA acts as a key regulator of YAP in CRC and that ALDOA’s carcinogenic properties are dependent on YAP.

YAP is usually regulated by the canonical Hippo/LATS cascade, but it can also be activated by other pathways, such as the AMPK signaling cascade [32–34]. Previous studies [32–34] indicated that AMPK-mediated regulation of YAP occurs in response to cellular energy stress and an oncogenic function of YAP activates the glycolytic pathway, thereby providing critical crosstalk between energy homeostasis and the Hippo cascade in the occurrence of carcinoma. In the present study, RNA-Seq experiment on HT29 cells (siALDOA and control) revealed that the positive and negative signatures of YAP signaling cascade in cells were significantly enriched (Fig. 4D, E, *P* were 0.002 and 0.006, respectively), suggesting the potential link between the ALDOA gene and YAP signaling pathway. Following multiple experiments confirmed the regulation of YAP by ALDOA. To investigate whether ALDOA regulates YAP through the canonical LATS1-dependent pathway, we examined LATS1 phosphorylation by ALDOA genetic manipulation in CRC cells. The results showed that ALDOA had minimal effect on LATS1 phosphorylation, indicating there might be other molecules involved in the regulation of YAP by ALDOA in CRC cells.

ALDOA, as a central enzyme in the glycolysis process, was identified as a metabolic regulator of cardiac hypertrophy by inhibiting AMPK activation [37]. ALDOA has also been shown to inhibit AMPK activation in cancer cells, possibly as a result of higher cellular ATP levels caused by enhanced glycolysis [38–40]. Our study further confirmed the inhibitory effect of ALDOA in AMPK activation in CRC cells and evidenced that ALDOA-induced YAP activation is dependent on AMPK. However, a recent study [41] demonstrated that ALDOA triggered the activation of AMPK, which was responsible for the promalignant activities in ALDOA-expressing cells. In this view, the underlined data suggest that ALDOA’s effect on various tumor tissues may be context-dependent, and the precise molecular pathways driving the discrepancy will require further investigation in the future.

The canonical activation of AMPK is dependent on a decreasing cellular energy state, which is shown by increasing AMP/ATP and ADP/ATP concentrations [42, 43]. The AMPK cascade can also be activated by glucose depletion via a non-canonical pathway involving a complex comprising the v-ATPase, regulators, AXIN, LKB1, and AMPK (v-ATPase-regulator-AXIN/LKB1 complex) [44, 45], which occurs regardless of the AMP/ATP or ADP/ATP concentrations. In low cellular glucose, the absence of FBP from aldolase induces conformation changes in its interaction with the AXIN-based AMPK-activation complex, which triggers AMPK phosphorylation [42, 43]. These results indicate that aldolase is a sensor of glucose availability and regulates AMPK in an AMP/ADP-independent manner [43]. Taken together, we suggest that two possible mechanisms are underlying the ALDOA regulation of AMPK. First, silencing ALDOA expression inhibits cellular glycolysis and ATP production, hence decreased levels of ATP/ADP leads to phosphorylation of AMPK. Second, knockdown of ALDOA triggers the formation of v-ATPase-regulator-AXIN/LKB1 complex, which activates AMPK in an AMP/ADP-independent manner.

Although extensive research is needed to elucidate the processes underlying ALDOA’s modulation of AMPK, our study revealed ALDOA as a new metabolic regulator of YAP via inhibiting the AMPK signaling cascade. The combination of ALDOA and YAP inhibition may provide a novel approach for CRC treatment.

## Materials and methods

### Clinical data

The appropriate clinical information was acquired from the GEPIA database (http://gepia.cancer-pku.cn/) and the public GEO (Gene Expression Omnibus) databases (https://www.ncbi.nlm.nih.gov/gds/). The Kaplan–Meier (K–M) curves involved were downloaded from the K–M Plotter (https://kmplot.com) database. Fresh tissue samples were obtained from the First Affiliated Hospital of Soochow University from May 2022 to December 2023. Before the surgical procedure, the patients have not received radiation or chemotherapy. All patients gave their written informed consent. The Institute Research Medical Ethics Committee at the First Affiliated Hospital of Soochow University approved this study. This research was conducted following the Helsinki Declaration. Supplementary Table S1 illustrates the clinical features of patients.

### Cell culture and transfection

The Chinese Academy of Sciences Committee Type Culture Collection cell bank (Shanghai, China) provided the five human CRC cell lines (CaCo2, HT29, SW480, DLD-1, and SW620). The culturing of cells was carried out in DMEM or RPMI media (at 37 °C and 5% CO_2_) enriched with Fetal Bovine Serum (FBS, 10%) and streptomycin/penicillin (1%), and all these reagents were obtained from Gibco, USA.

Genechem Company produced the Lentivirus vectors expressing ALDOA or short hairpin RNA (shRNA) targeting ALDOA, as well as their control vectors. The transfection was carried out following the suggestions provided by the manufacturer, as detailed in our previous study [46, 47]. Genepharma Company generated the small interfering RNAs (siRNAs). Lipofectamine RNAiMax (Invitrogen) was utilized to transfect siRNAs (at 20 nM concentration), based on the manufacturer’s recommendations. The human ALDOA shRNA sequence is 5′-CCA TGC TTG CAC TCA GAA GTT-3′. The human AMPK siRNA sequence is 5′-GCA GAA GUA UGU AGA GCA AUC-3′ and 5′-CGC TGA GTA CTT CGA AAT GTC-3′ (siAMPK#2). The human YAP siRNA sequence is 5′-GGA AUU GAG AAC AAU GAC GUU-3′ and 5′-GGU CAG AGA UAC UUC UUA AAU-3′ (siYAP#2). A scrambled siRNA 5′-UUC UCC GAA CGU GUC ACG UTT-3′ was used as a negative control.

### Cell viability and migration assay

Colony-forming and Transwell migration assays were conducted to evaluate the proliferative and migratory potential of the cells, accordingly as described earlier [46, 47].

### ATP measurement, glucose consumption, pyruvate production and lactate production

ATP production, glucose consumption, pyruvate production and lactate production were detected using ATP content assay kit (Solarbio), Glucose Uptake Colorimetric Assay Kit (BioVision), pyruvate content detection kit (Solarbio) and lactate content detection kit (Solarbio), respectively, according to the manufacturer’s protocol.

### Seahorse metabolic measurements

Cells were cultured in a conditioned medium and the glucose, oligomycin and 2-DG were added into the XF cell culture microplate. Then, the extracellular acidification rate (ECAR) was measured with the Seahorse XFe/XF analyzer.

### Immunohistochemistry (IHC)

The paraffin-embedded samples were stained with IHC as previously described [46, 47]. Briefly, 4-μm paraffin-embedded sections were deparaffinized and rehydrated, and then hydrogen peroxide (0.3%) was utilized to block these sections. Next, the incubation of these sections was carried out with the primary antibodies for 24 h at 4 °C, followed by washing with PBS. The incubation of these sections was then carried out with a Histostain Streptavidin-Peroxidase kit (SP9001), followed by observing through a staining kit, and these kits were obtained from Zhongshanjinqiao Biotechnology, China. In this study, the primary antibodies detecting human ALDOA, AREG and CTGF (dilution 1:100; Proteintech) were used.

### Immunofluorescence (IF) assay

The cells fixation was carried out with paraformaldehyde (4%) for 0.5 h, followed by extraction with 0.5% Triton X-100 solution (Sigma-Aldrich) at ~25 °C for 10 min. The cells were treated with the primary antibodies YAP (dilution 1:100; #14074, Cell Signaling Technology (CST)) for 60 min at ~25 °C after being blocked with goat serum (Zsbio, Beijing, China) for 0.5 h. at ~25 °C. PBS was used for washing purposes. The cells incubation was then carried out with a secondary antibody (Alexa Fluor-488 anti-rabbit) for 60 min at ~25 °C, followed by staining with DAPI at ~25 °C for 10 min. The anti-fade solution was being used to mount the coverslips onto glass slides, which were then examined using an epifluorescence microscope (Nikon).

### Western blotting

RIPA lysis buffer (Beyotime, China) was utilized to extract whole-cell lysates, which were then evaluated using a BCA protein assay kit (Pierce, USA). Proteins were separated using SDS-PAGE, followed by moving onto PVDF membranes. The membrane incubation was then carried out with the primary and secondary antibodies, accordingly, followed by visualizing through chemiluminescence. Antibodies used are provided in Supplementary Table S2.

### Quantitative real-time PCR (qRT-PCR)

TRIzol reagent (Thermo Fisher Scientific) was utilized to extract total RNAs, followed by reverse transcription using cDNA Synthesis SuperMix (TransGen Biotech), as suggested by the manufacturer. qPT-PCR was conducted with SYBR Green RT-PCR kits (ABI, USA) using an ABI 7500 Fast Real-Time PCR System (Applied Biosystems, UK), as suggested by the manufacturer. The sequencing information for the primers are provided in Supplementary Table S3.

### In vivo mouse model

Nanjing University’s Nanjing Biomedical Research Institute provided four-week-old male nude mice (BALB/c, SPF grade). For the tumorigenesis assay, the subcutaneous implantation of cells (5 × 10^6^ cells in 200 μL of PBS) was carried out into the groin of the mice (two groups, 3 mice/group). The mice were euthanized post 28 days, followed by weighing the subcutaneous tumors. For metastasis assay, cells (5 × 10^6^ cells/150 μL of PBS) were injected into the tail veins of the mice (two groups, 3 mice/group). Next, the mice were euthanized post 6 weeks, followed by measuring the metastatic nodules. The approval for in vivo experiments was provided by the Animal Ethics Committee of Soochow University (Suzhou, Jiangsu, PR China).

### RNA-Seq experiment

Total RNA was isolated from the HT29 cells (siALDOA and control) using Lipofectamine RNAiMax (Invitrogen; ThermoFisher) according to the manufacturer’s instructions. RNA-Seq libraries (with both siALDOA and control in triplicate) were constructed according to standard Illumina protocols. Novogene Biotech Co., Ltd (Beijing, China) generated Illumina sequencing reads (150 bp, paired-end) on the NovaSeq X Plus platform (Illumina). The raw data of this RNA-Seq experiment have been deposited on GEO with the accession number GSE277845.

### Bioinformatics analysis

Gene expression analysis and gene set enrichment analysis (GSEA) were performed as described in the Supplementary Materials and Methods.

### Statistical evaluations

All experiments were repeated for three times and data were indicated as means ± SEM. Student’s *t-test* or one way ANOVA was used to evaluate the variations between groups. The association between the expression of ALDOA and clinicopathological characteristics was identified using Fisher’s exact or *χ*^2^ test. Spearman rank correlation analysis was used to calculate the correlations between protein expression levels. *P*-value < 0.05 was regarded as statistically considerable.

## Supplementary information

ALDOA CRC Supplementary data

Original Western Blots

## Data Availability

The datasets used and/or analyzed during the current study are available from the corresponding author on reasonable request.
