# Supplementary material for: ALDOA contributes to colorectal tumorigenesis and metastasis by targeting YAP
Source: Cell Death Discov. 2025 Jan 5;10:489. doi: 10.1038/s41420-024-02249-z (PMC11700148; doi:10.1038/s41420-024-02249-z)
Supplement: Supplementary file 1 — ALDOA CRC Supplementary data [file 41420_2024_2249_MOESM1_ESM.docx]

**ALDOA contributes to colorectal tumorigenesis and metastasis by targeting YAP**

Liang Sun^1,#^, Ting Lu^2,#^, Linhua Jiang^1,#^, Huihui Yao^1^, Qixuan Xu^1^, Jie Sun^1^, Xiaoqin Yang^3^, Songbing He^1^ and Xinguo Zhu^1^

^1^ Department of General Surgery, the First Affiliated Hospital of Soochow University, Suzhou, 215006, China

^2^ Department of Ultrasound, the First Affiliated Hospital of Soochow University, Suzhou 215006, China.

^3^School of Basic Medical Sciences, Suzhou Medical College of Soochow University, Suzhou 215006, China.

^#^ Co-first author

**Corresponding Author**:

Xinguo Zhu, Department of General Surgery, the First Affiliated Hospital of Soochow University, Suzhou, 215006, China. E-mail: [zhuxinguo1970@163.com](mailto:zhuxinguo1970@163.com).

Songbing He, Department of General Surgery, the First Affiliated Hospital of Soochow University, Suzhou, 215006, China. E-mail: [captain_hsb@163.com](mailto:captain_hsb@163.com).

Xiaoqin Yang, School of Basic Medical Sciences, Suzhou Medical College of Soochow University, Suzhou 215006, China. E-mail: [yangxiaoqin@suda.edu.cn](mailto:yangxiaoqin@suda.edu.cn).

**Materials and Methods**

**Bioinformatics analysis**

Raw reads were aligned back to the human reference genome (hg38) by tophat2 software (version 2.1.1). [1] Read counts for each gene were calculated by gfold software (version 1.1.4). [2] Further differentially expressed genes were identified by the R language workflow of “limma” (version 3.60.4) [3] and “edgeR” (version 4.2.1) [4] packages. A P < 0.01 indicated a significantly differentially expressed gene. Function enrichment analyses for the differential genes were conducted by Toppgene webserver (https://toppgene.cchmc.org/enrichment.jsp). [5] For the published RNA-Seq dataset for YAP/TAZ Knockdown in the HCT116 cell line (GSE176475)[6], the top 100 up-regulated and top 100 down-regulated genes ranked according to the P-values were defined as the signatures for YAP signaling pathways in colon cancer. Further gene set enrichment analysis (GSEA) was performed to determine the functional character difference between the ALDOA knockdown and control treatments (Scoring scheme: classic model; Permute: gene set). [7] A P < 0.05 indicated a significant enrichment.

[1] Kim, D.; Pertea, G.; Trapnell, C.; Pimentel, H.; Kelley, R.; Salzberg, S. L., TopHat2: accurate alignment of transcriptomes in the presence of insertions, deletions and gene fusions. Genome Biology 2013, 14 (4), R36.

[2] Feng, J.; Meyer, C. A.; Wang, Q.; Liu, J. S.; Shirley Liu, X.; Zhang, Y., GFOLD: a generalized fold change for ranking differentially expressed genes from RNA-seq data. Bioinformatics 2012, 28 (21), 2782-8.

[3] Ritchie, M. E.; Phipson, B.; Wu, D.; Hu, Y.; Law, C. W.; Shi, W.; Smyth, G. K., limma powers differential expression analyses for RNA-sequencing and microarray studies. Nucleic Acids Res 2015, 43 (7), e47.

[4] Robinson, M. D.; McCarthy, D. J.; Smyth, G. K., edgeR: a Bioconductor package for differential expression analysis of digital gene expression data. Bioinformatics 2010, 26 (1), 139-40.

[5] Chen, J.; Bardes, E. E.; Aronow, B. J.; Jegga, A. G., ToppGene Suite for gene list enrichment analysis and candidate gene prioritization. Nucleic Acids Res 2009, 37 (Web Server issue), W305-11.

[6] Guo, Y.; Zhu, Z.; Huang, Z.; Cui, L.; Yu, W.; Hong, W.; Zhou, Z.; Du, P.; Liu, C. Y., CK2-induced cooperation of HHEX with the YAP-TEAD4 complex promotes colorectal tumorigenesis. Nat Commun 2022, 13 (1), 4995.

[7] Subramanian, A.; Tamayo, P.; Mootha, V. K.; Mukherjee, S.; Ebert, B. L.; Gillette, M. A.; Paulovich, A.; Pomeroy, S. L.; Golub, T. R.; Lander, E. S.; Mesirov, J. P., Gene set enrichment analysis: a knowledge-based approach for interpreting genome-wide expression profiles. Proc Natl Acad Sci U S A 2005, 102 (43), 15545-50.

**
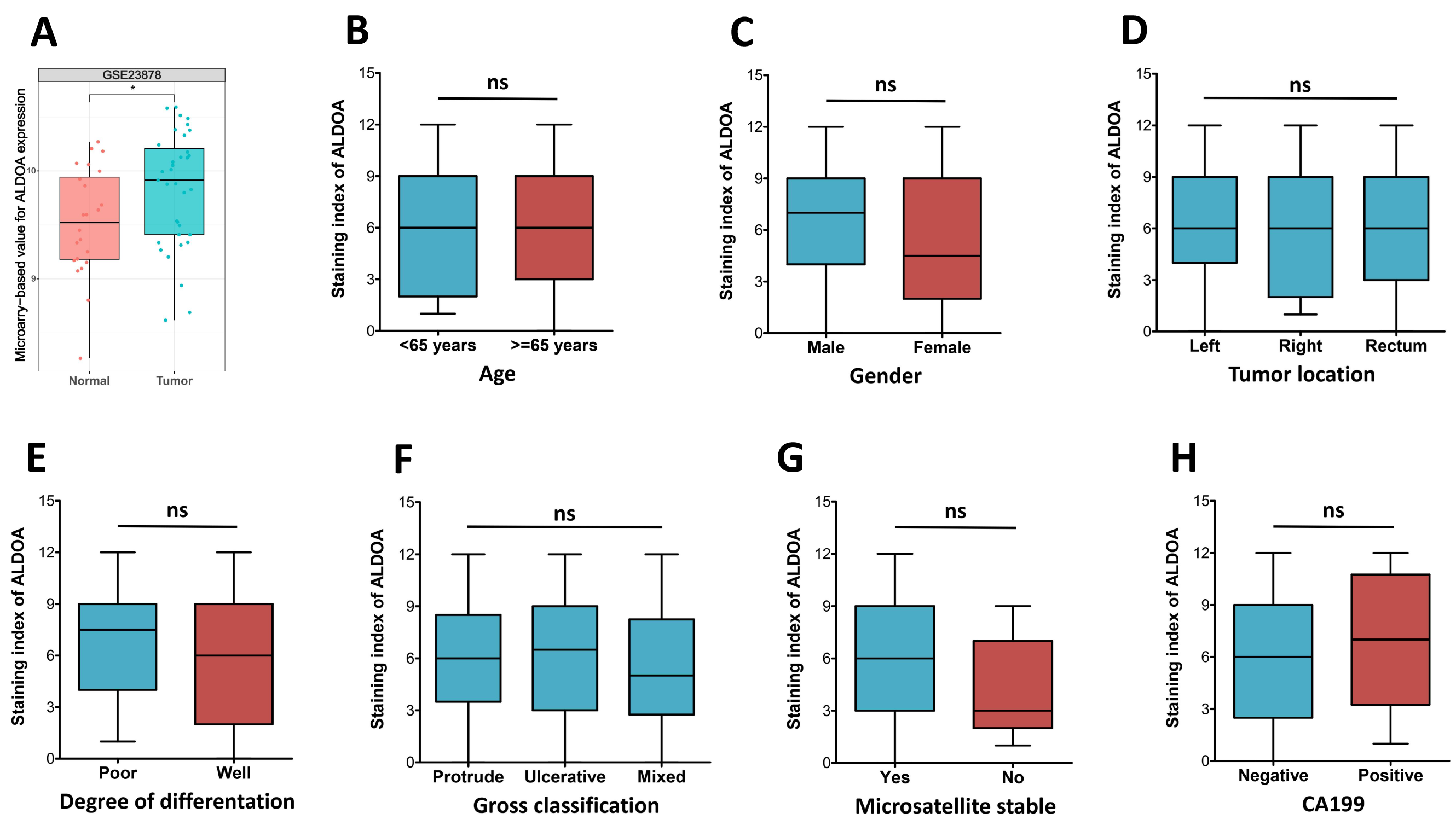
**

**Supplementary Figure S1**. ALDOA expression in CRC tissues. (A) ALDOA gene expression in CRC tissues and cell lines derived from the GSE23878 dataset. (B-H) IHC staining to analyze ALDOA expression in CRC tissues of different age (B, < 65 years vs. >= 65 years), gender (C, Male vs. Female), Tumor location (D, Left vs. Right vs. Rectum), Degree of differentiation (E, Poor vs. Well), Gross classification (F, Protrude type vs. Ulcerative type vs. Mixed type), Microsatellite stable (G, Yes vs. No) and serum CA199 level (H, Negative vs. Positive). ns, nonsignificant, **P* < 0.05.

**
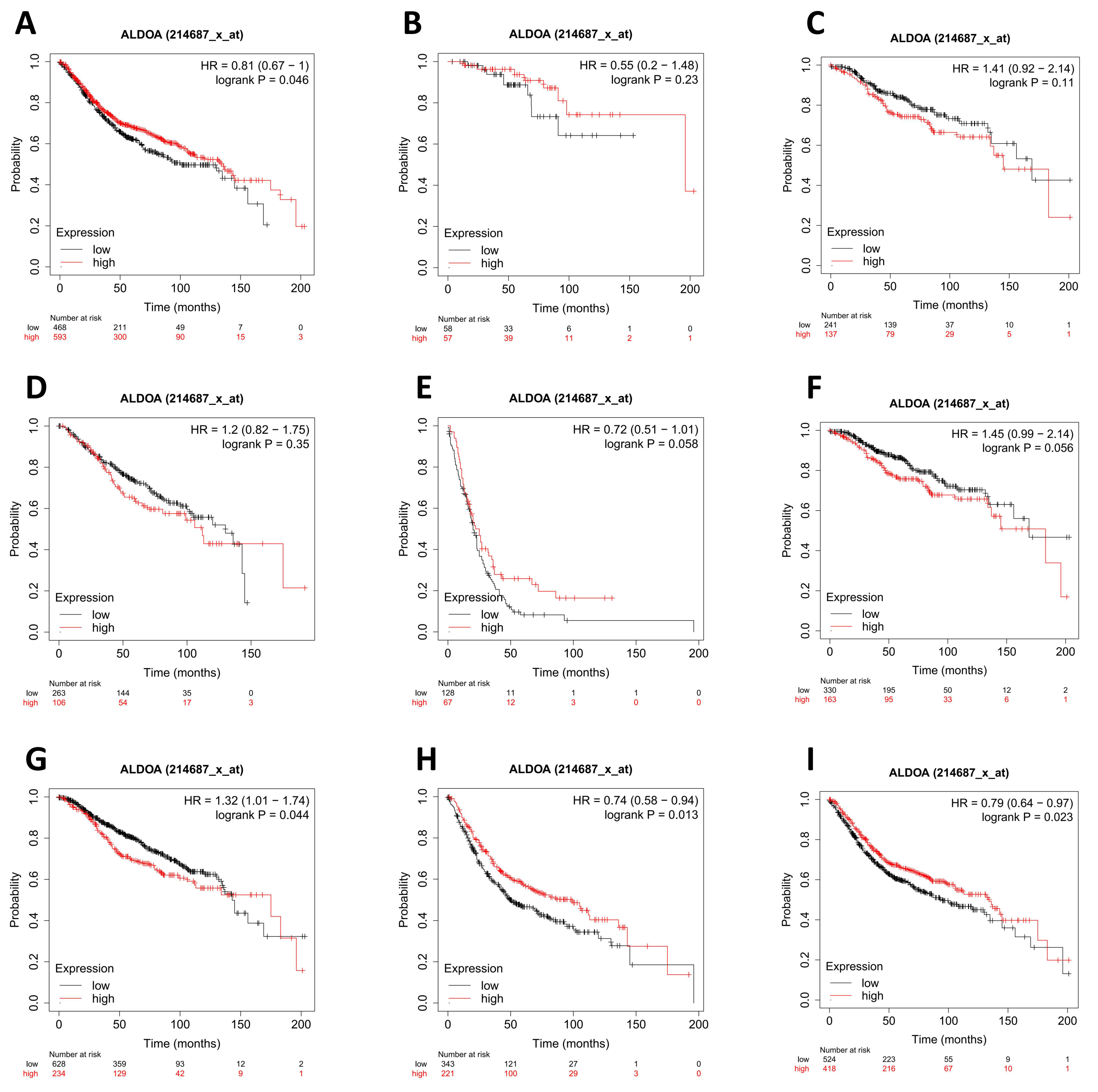
**

**Supplementary Figure S2**. ALDOA mRNA expression in different stages of CRC correlates with overal survival (OS) of CRC patients from the Kaplan-Meier plotter databases. (A) Kaplan-Meier curve for the OS of CRC patients. (B-E) Kaplan-Meier curve for the OS of CRC patients with different tumor staging (stage I (B), stage II (C), stage III (D), stage IV (E)). (F-I) Kaplan-Meier curve for the OS of CRC patients with different tumor staging (stage I-II (F), stage I-III (G), stage III-IV (H), stage II-IV (I)).

**
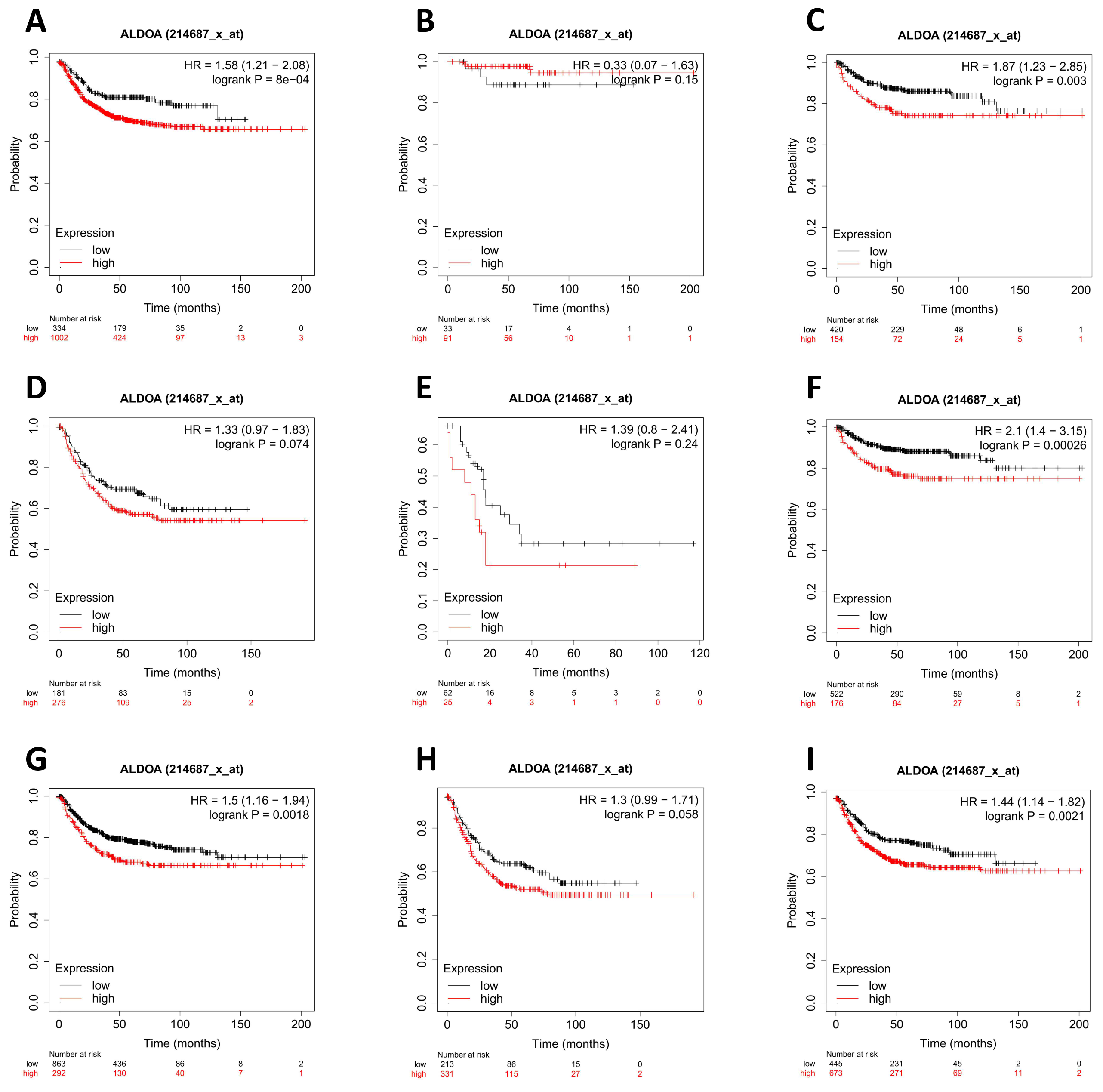
**

**Supplementary Figure S3**. ALDOA mRNA expression in different stages of CRC correlates with relapse-free survival (RFS) of CRC patients from the Kaplan-Meier plotter databases. (A) Kaplan-Meier curve for the RFS of CRC patients. (B-E) Kaplan-Meier curve for the RFS of CRC patients with different tumor staging (stage I (B), stage II (C), stage III (D), stage IV (E)). (F-I) Kaplan-Meier curve for the RFS of CRC patients with different tumor staging (stage I-II (F), stage I-III (G), stage III-IV (H), stage II-IV (I)).

**
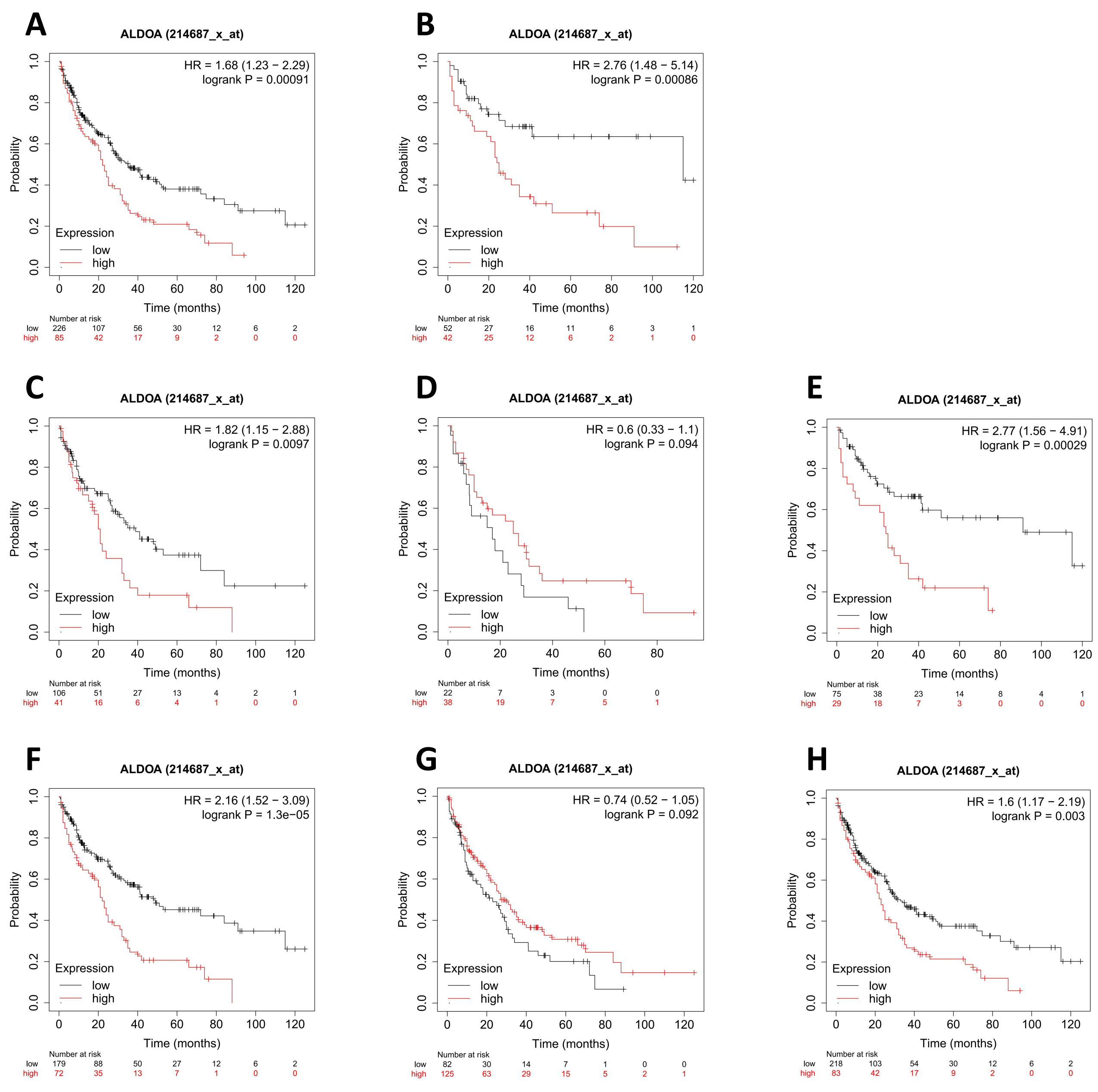
**

**Supplementary Figure S4**. ALDOA mRNA expression in different stages of CRC correlates with post-progression survival (PPS) of CRC patients from the Kaplan-Meier plotter databases. (A) Kaplan-Meier curve for the PPS of CRC patients. (B-D) Kaplan-Meier curve for the PPS of CRC patients with different tumor staging (stage I (B), stage III (C), stage IV (D)). (E-H) Kaplan-Meier curve for the PPS of CRC patients with different tumor staging (stage I-II (E), stage I-III (F), stage III-IV (G), stage II-IV (H)).

**
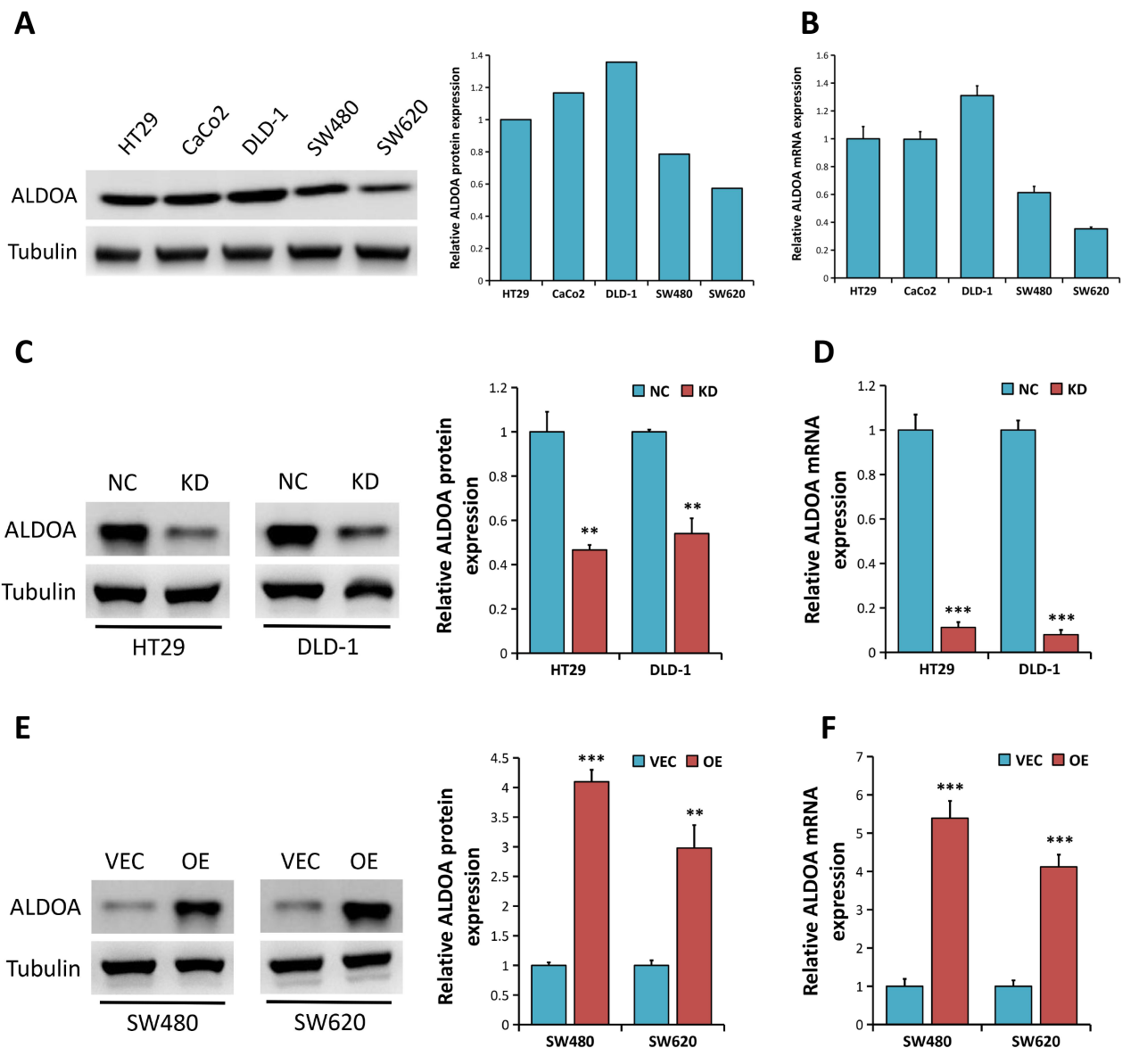
**

**Supplementary Figure S5.** ALDOA expression in CRC cell lines. (A) Western blotting analysis of ALDOA protein level in five CRC cell lines (HT29, CaCo2, DLD-1, SW480, SW620). (B) qRT-PCR analysis of ALDOA mRNA level in five CRC cell lines (HT29, CaCo2, DLD-1, SW480, SW620). (C-D) Western blotting (C) and qRT-PCR (D) analysis of ALDOA expression in control (NC) and shALDOA-infected (KD) HT29 and DLD-1 cells. (E-F) Western blotting (E) and qRT-PCR (F) analysis of ALDOA expression in SW480 and SW620 cells transfected with empty vector (VEC) and Lenti-ALDOA (OE). ***P* < 0.01, ****P* < 0.001.


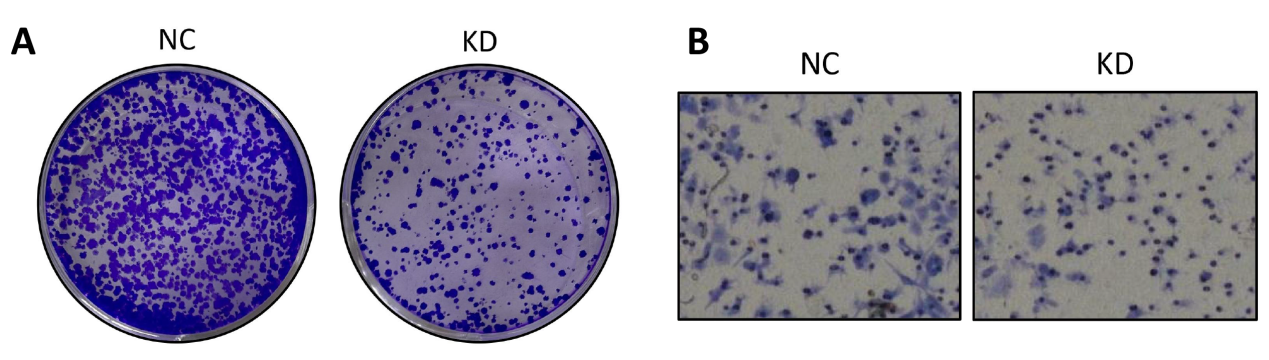


**Supplementary Figure S6.** ALDOA knockdown inhibits the proliferation and migration of DLD-1 cells. (A) The cell proliferation ability was examined by colony formation assays in DLD-1 cells (NC vs. KD). (B) The migration capacity was evaluated by Transwell assays in DLD-1 cells (NC vs. KD).


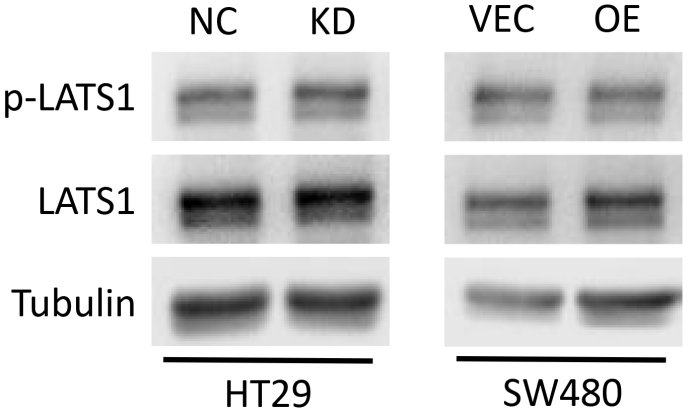


**Supplementary Figure S7.** Western blotting analysis of p-LATS1 and LATS1 expression in HT-29 cells (NC vs. KD) SW480 cells (VEC vs. OE).


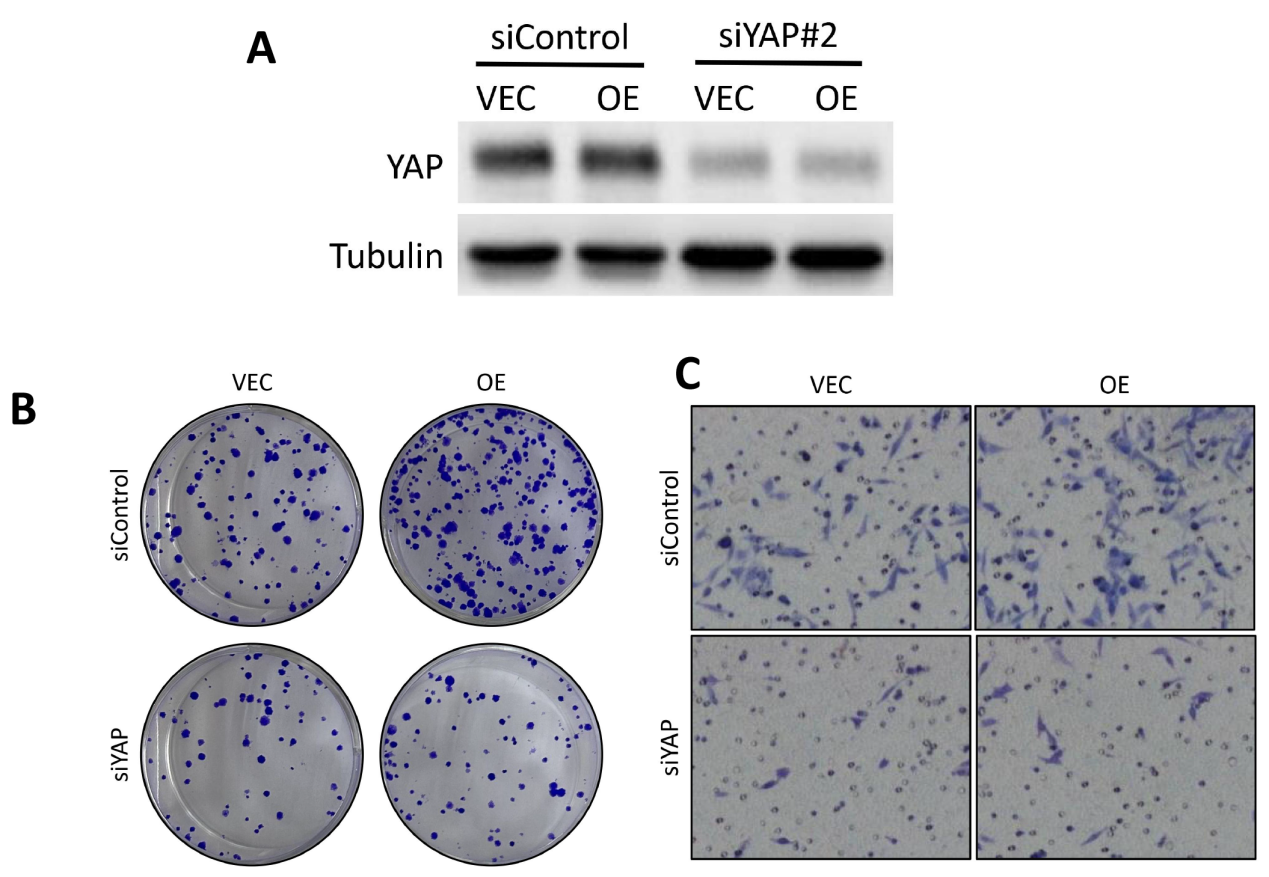


**Supplementary Figure S8.** ALDOA promotes CRC cell proliferation and migration in a YAP-dependent manner. (A) Western blotting analysis of YAP expression in SW480 cells (VEC vs. OE) treated with siRNA against YAP (siYAP#2) and siControl. (B) The cell proliferation ability was examined by colony formation assays in SW480 cells (VEC vs. OE) treated with siYAP#2 and siControl. (C) The migration capacity was evaluated by Transwell assays in SW480 cells (VEC vs. OE) treated with siYAP#2 and siControl.


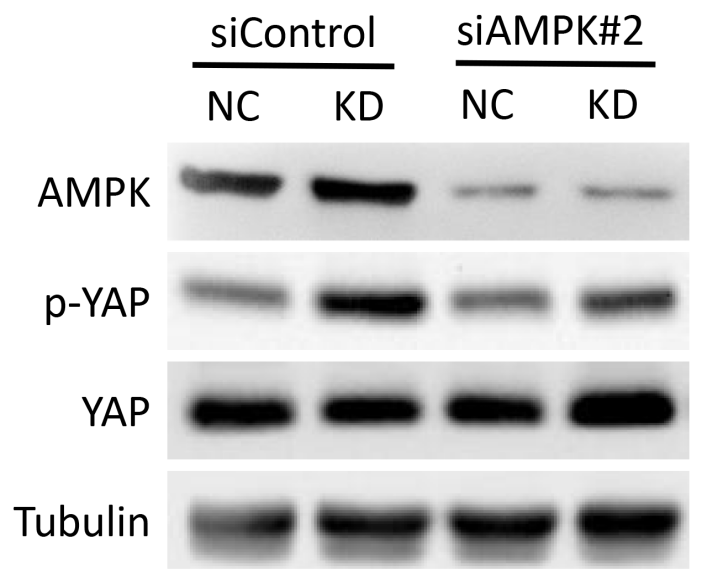


**Supplementary Figure S9.** Western blotting analysis of the indicated proteins in HT-29 cells (NC vs. KD) treated with siRNA against AMPK (siAMPK#2) and siControl.

**Supplementary Table S1.** Relationship between ALDOA and clinico-pathological features of CRC patients.

|  | **No.** | **ALDOA expression** | | ***χ*^2^** | ***P* value** |
| --- | --- | --- | --- | --- | --- |
|  |  | **None or low** | **High** |  |  |
| **Total** | 129 | 52 (40.3%) | 77 (59.7%) |  |  |
| **Age (years)** |  |  |  |  |  |
| <65 | 46 | 18 (39.1%) | 28 (60.9%) | 0.041 | 0.839 |
| >=65 | 83 | 34 (41.0%) | 49 (59.0%) |  |  |
| **Gender** |  |  |  |  |  |
| Male | 83 | 29 (34.9%) | 54 (65.1%) | 2.790 | 0.095 |
| Female | 46 | 23 (50.0%) | 23 (50.0%) |  |  |
| **Tumor size** |  |  |  |  |  |
| <5cm | 76 | 40 (52.6%) | 36 (47.4%) | 11.672 | 0.001** |
| >=5cm | 53 | 12 (22.6%) | 41 (77.4%) |  |  |
| **Tumor location** |  |  |  |  |  |
| Left-sided colon | 36 | 13 (36.1%) | 23 (63.9%) | 1.949 | 0.377 |
| Right-sided colon | 43 | 21 (48.8%) | 22 (51.2%) |  |  |
| Rectum | 50 | 18 (36.0%) | 32 (64.0%) |  |  |
| **Gross classification** |  |  |  |  |  |
| Protrude type | 37 | 15 (40.5%) | 22 (59.5%) | 0.049 | 0.976 |
| Ulcerative type | 88 | 31 (35.2%) | 47 (64.8%) |  |  |
| Mixed type | 14 | 6 (42.9%) | 8 (57.1%) |  |  |
| **Degree of differentiation** |  |  |  |  |  |
| Poor | 46 | 16 (34.8%) | 30 (65.2%) | 0.908 | 0.341 |
| Well | 83 | 36 (43.4%) | 47 (56.6%) |  |  |
| **Vascular invasion** |  |  |  |  |  |
| Yes | 4 | 0 (0.0%) | 4 (100.0%) | - | 0.148 |
| No | 125 | 52 (41.6%) | 73 (58.4%) |  |  |
| **Nerve invasion** |  |  |  |  |  |
| Yes | 18 | 5 (27.8%) | 13 (72.2%) | 1.365 | 0.243 |
| No | 111 | 47 (42.3%) | 64 (57.7%) |  |  |
| **Microsatellite stable** |  |  |  |  |  |
| Yes | 120 | 47 (39.2%) | 73 (60.8%) | - | 0.483 |
| No | 9 | 5 (55.6%) | 4 (44.0%) |  |  |
| **Depth of invasion** |  |  |  |  |  |
| T1-2 | 5 | 5 (100.0%) | 0 (0.0%) | - | 0.009** |
| T3-4 | 124 | 47 (37.9%) | 77 (62.1%) |  |  |
| **Lymph node metastasis** |  |  |  |  |  |
| N0 | 71 | 39 (54.9%) | 32 (45.1%) | 13.851 | 0.001** |
| N1 | 43 | 11 (25.6%) | 32 (74.4%) |  |  |
| N2 | 15 | 2 (13.3%) | 13 (86.7%) |  |  |
| **TNM stage** |  |  |  |  |  |
| I-II | 70 | 39 (55.7%) | 31 (44.3%) | 15.094 | 0.000*** |
| III-IV | 59 | 13 (22.0%) | 46 (78.0%) |  |  |

** *P* < 0.01, *** *P* < 0.001

**Supplementary Table S2.** Antibody information

| **Name** | **Company** | **Catalog Number** |
| --- | --- | --- |
| ALDOA | Proteintech | 11217-1-AP |
| CTGF | Proteintech | 23936-1-AP |
| AREG | Proteintech | 16036-1-AP |
| Tubulin | Proteintech | 11224-1-AP |
| Lamin B | Proteintech | 12987-1-AP |
| GAPDH | Beyotime | AG019 |
| Phospho-YAP (Ser127) | Cell Signaling Technology | 57706 |
| YAP | Cell Signaling Technology | 14074 |
| Phospho-AMPKα (Thr172) | Cell Signaling Technology | 2535 |
| AMPKα | Cell Signaling Technology | 2532 |
| Phospho-LATS1 | Cell Signaling Technology | 8654 |
| LATS1 | Cell Signaling Technology | 3477 |

**Supplementary Table S3.** Primers used for qRT-PCR analysis.

| **Gene** | **Forward primer (5’-3’)** | **Reverse primer (5’-3’)** |
| --- | --- | --- |
| *ALDOA* | ATGCCCTACCAATATCCAGCA | GCTCCCAGTGGACTCATCTG |
| *CTGF* | CCTGGTCCAGACCACAGAGT | TGTCTTCATGCTGGTGCAG |
| *AREG* | GCTCTTGATACTCGGCTCAG | CCCGAGGACGGTTCACTAC |
| *18s* | GTAACCCGTTGAACCCCATT | CCATCCAATCGGTAGTAGCG |
