# Supplementary material for: ALDOA contributes to colorectal tumorigenesis and metastasis by targeting YAP
Source: Cell Death Discov. 2025 Jan 5;10:489. doi: 10.1038/s41420-024-02249-z (PMC11700148; doi:10.1038/s41420-024-02249-z)

Figure 5A p-YAP

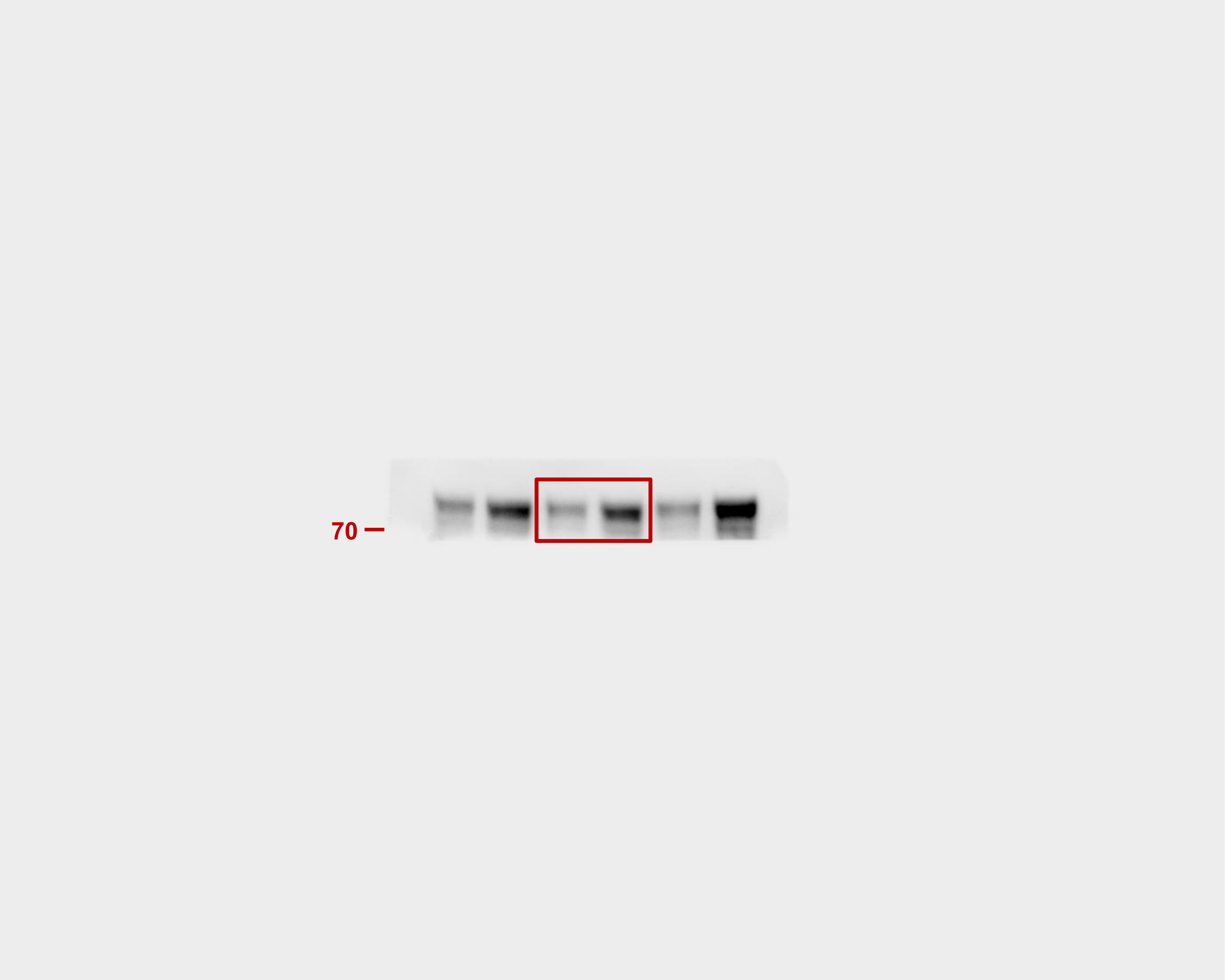

Figure 5A p-YAP

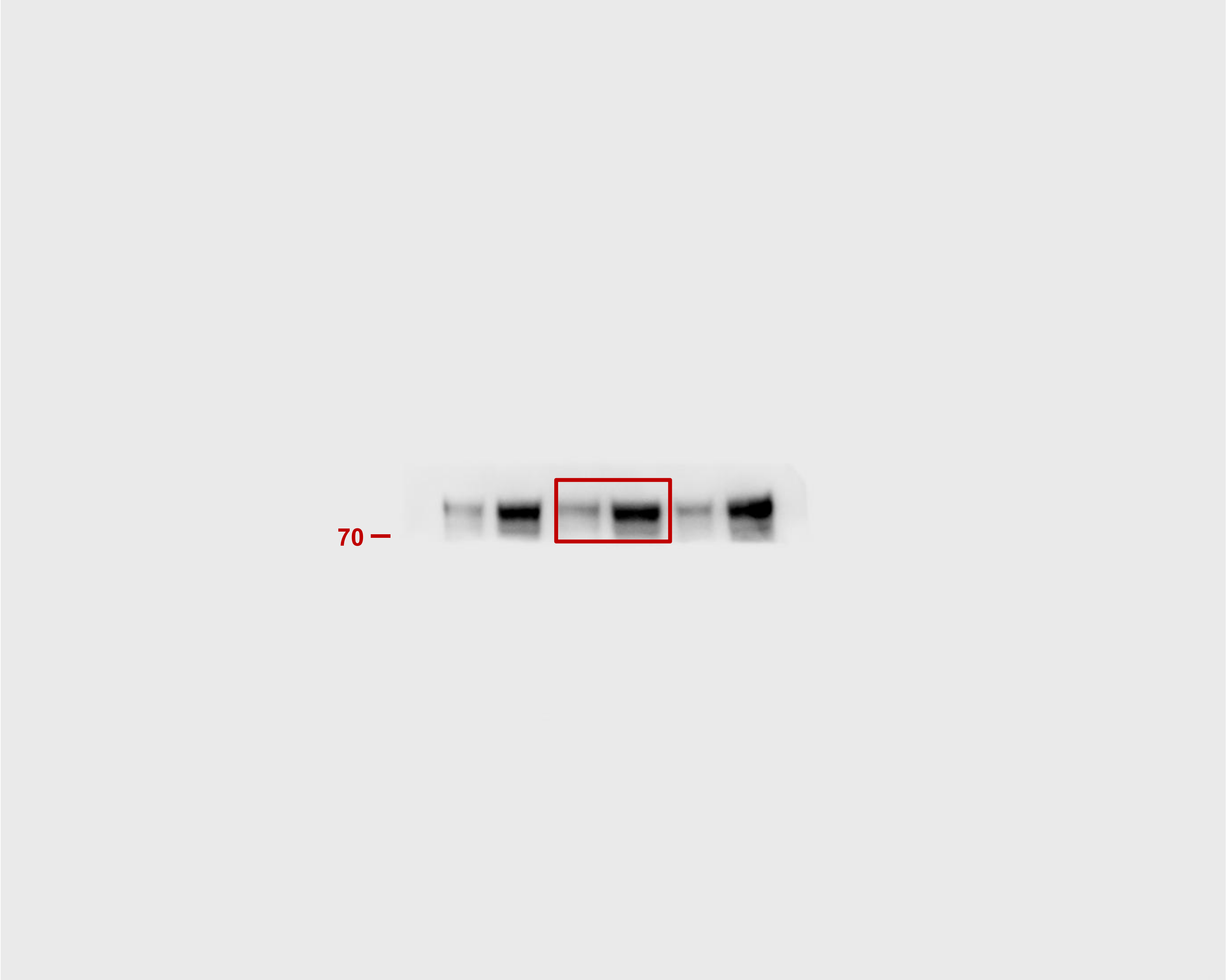

Figure 5A Tubulin

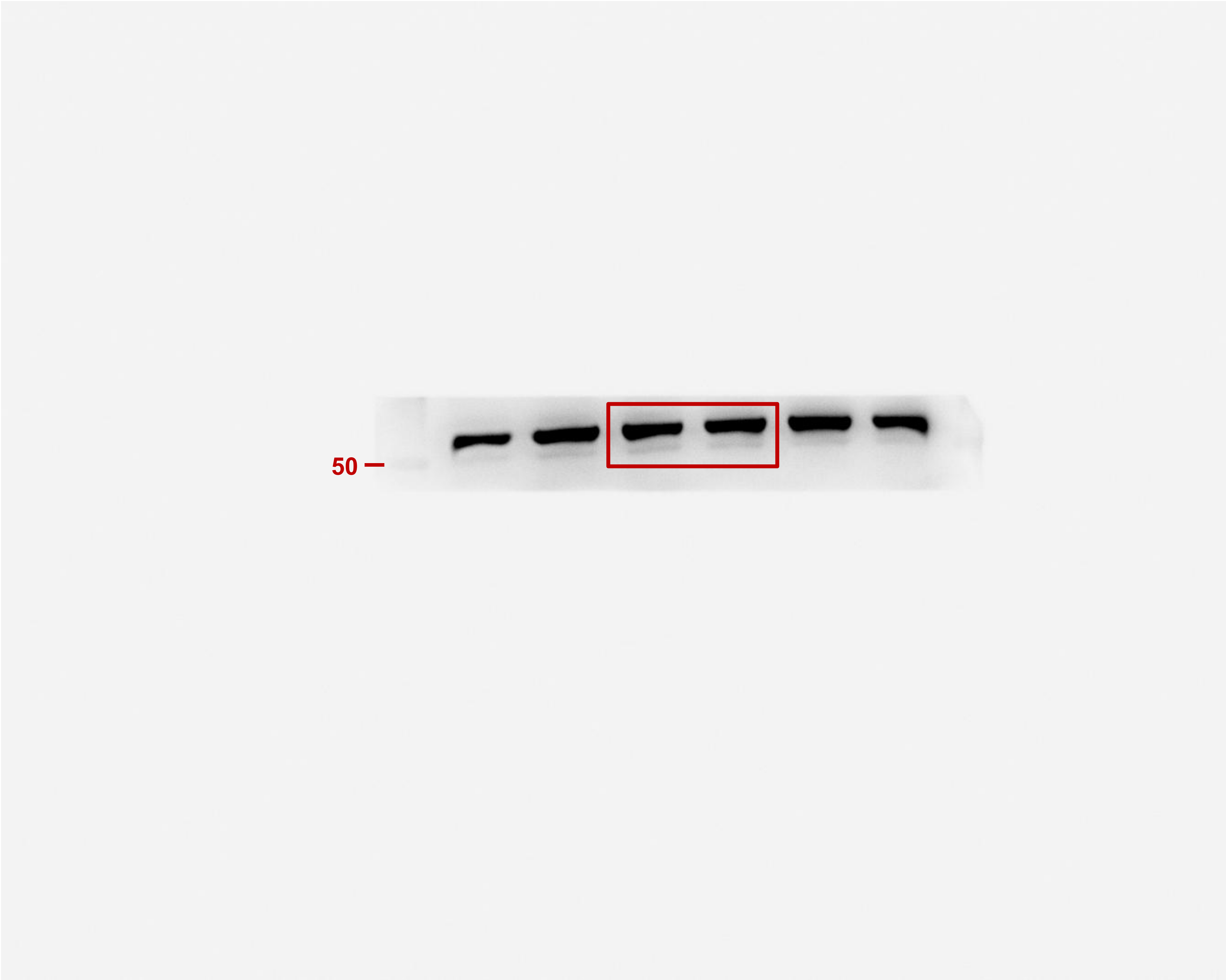

Figure 5A Tubulin

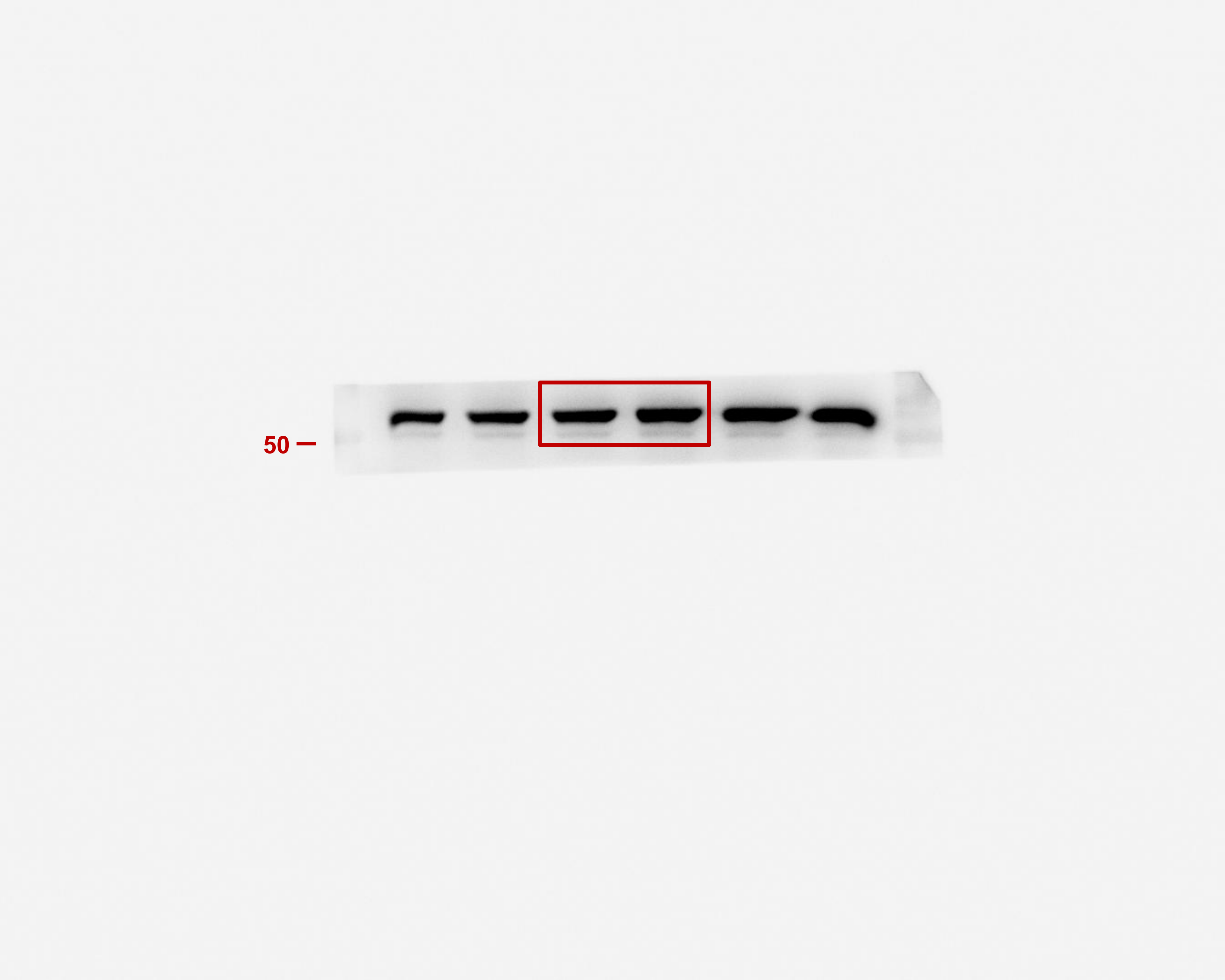

Figure 5B p-YAP

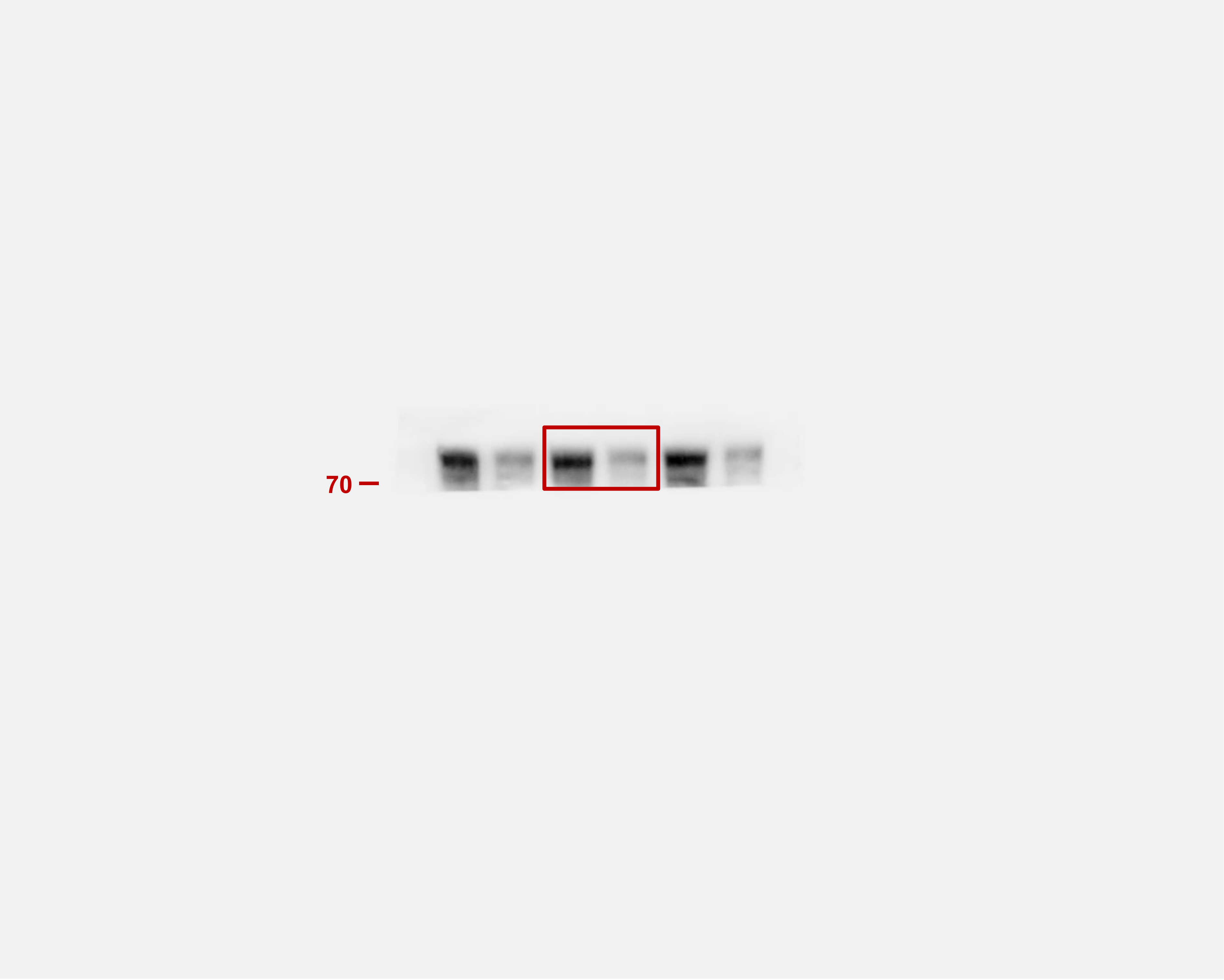

Figure 5B p-YAP

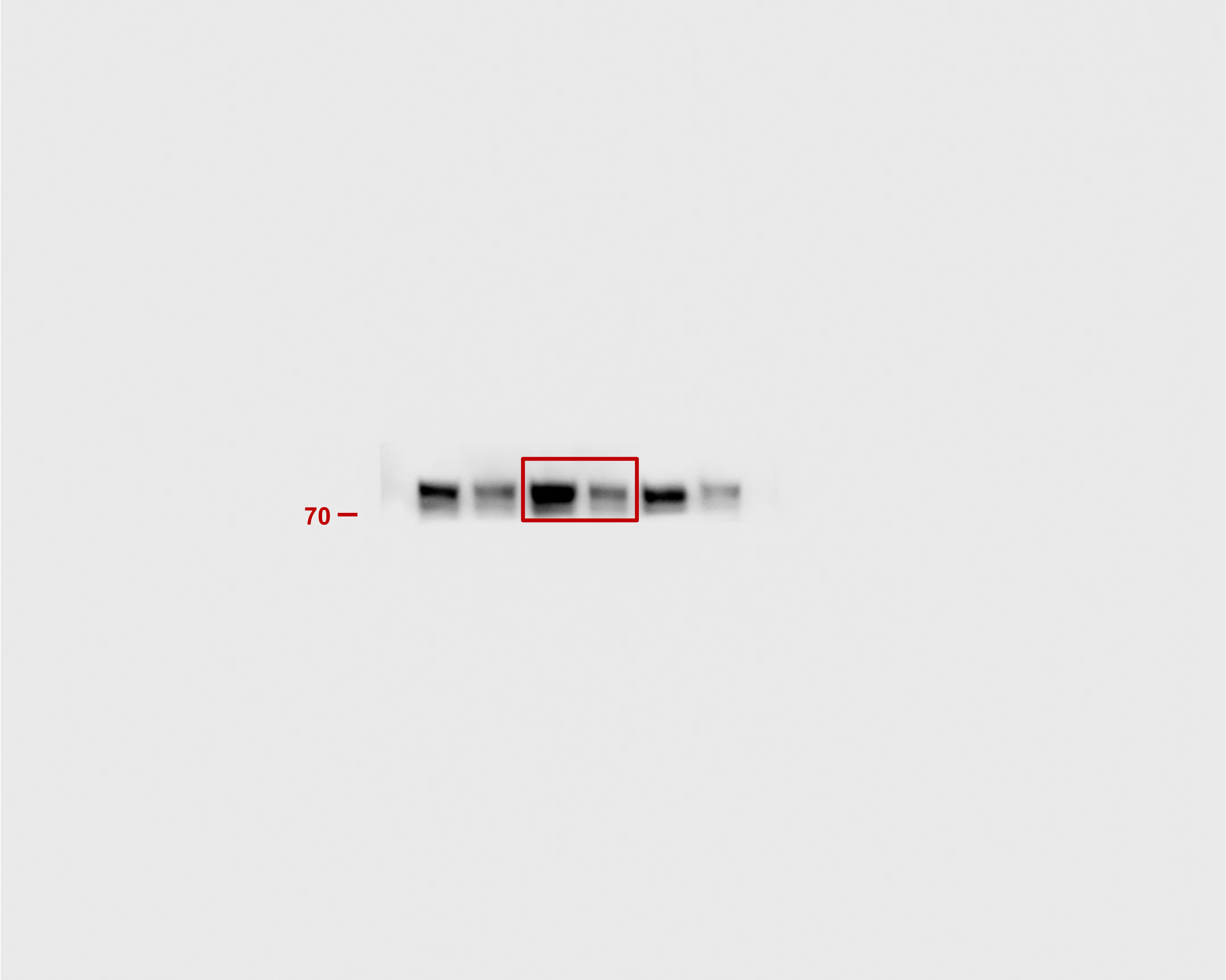

Figure 5A and 5B YAP

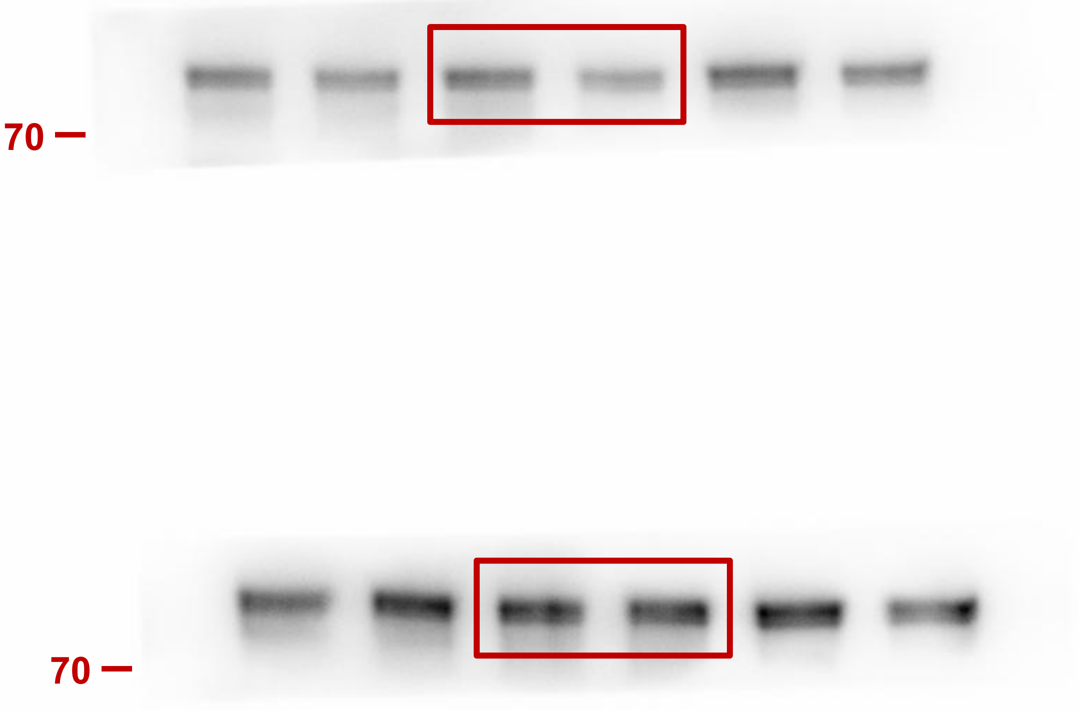

Figure 5A and 5B YAP

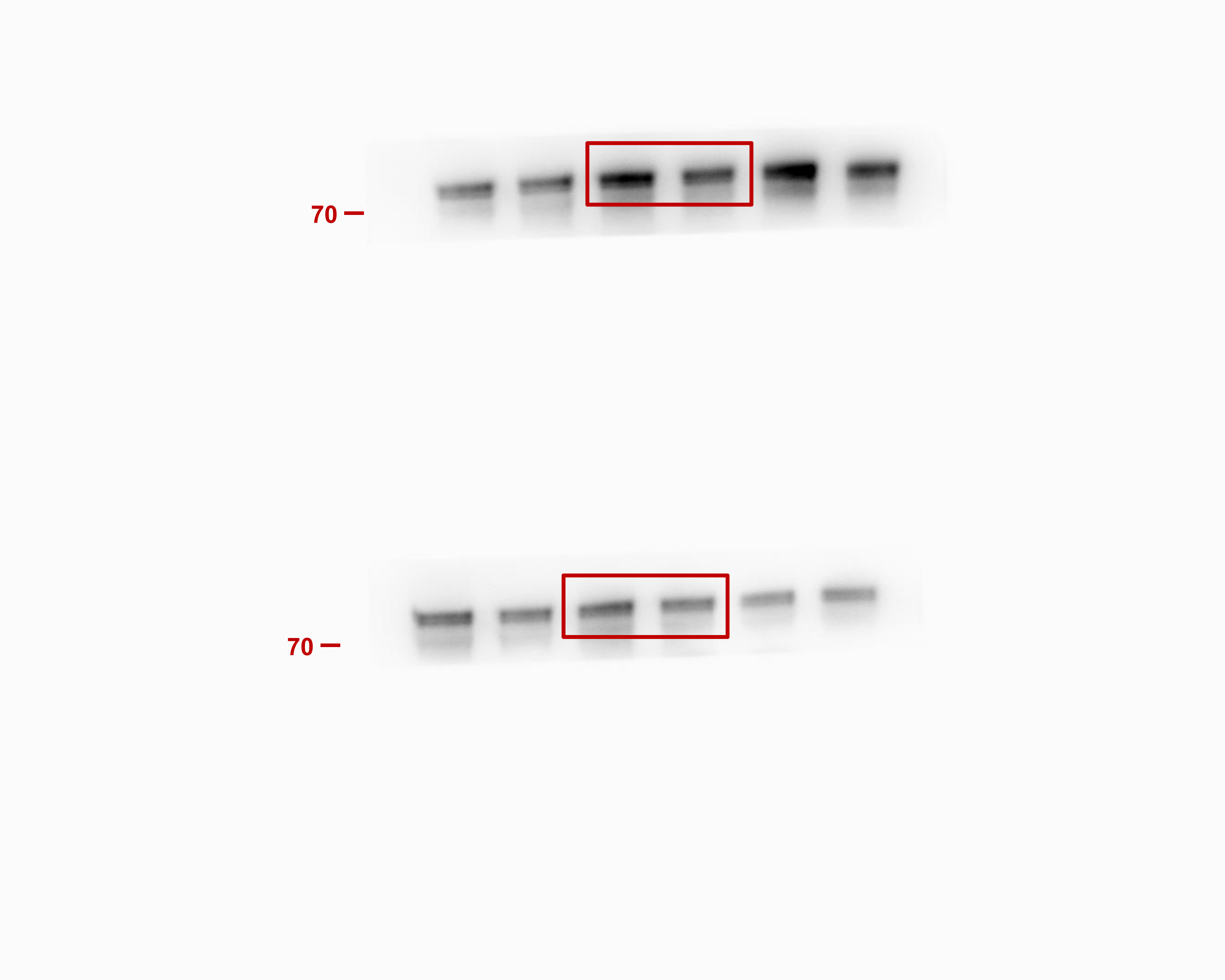

Figure 5B Tubulin

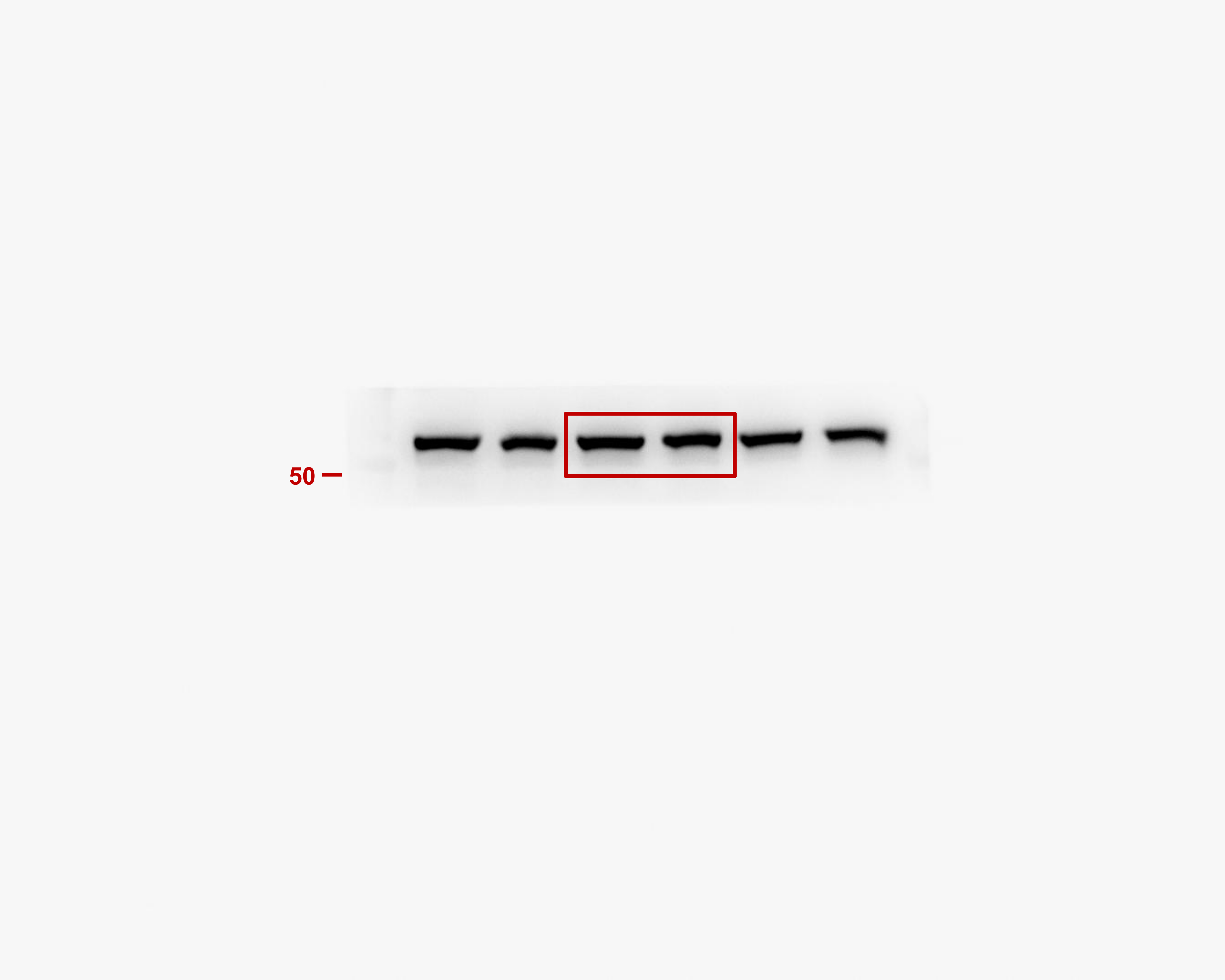

Figure 5B Tubulin

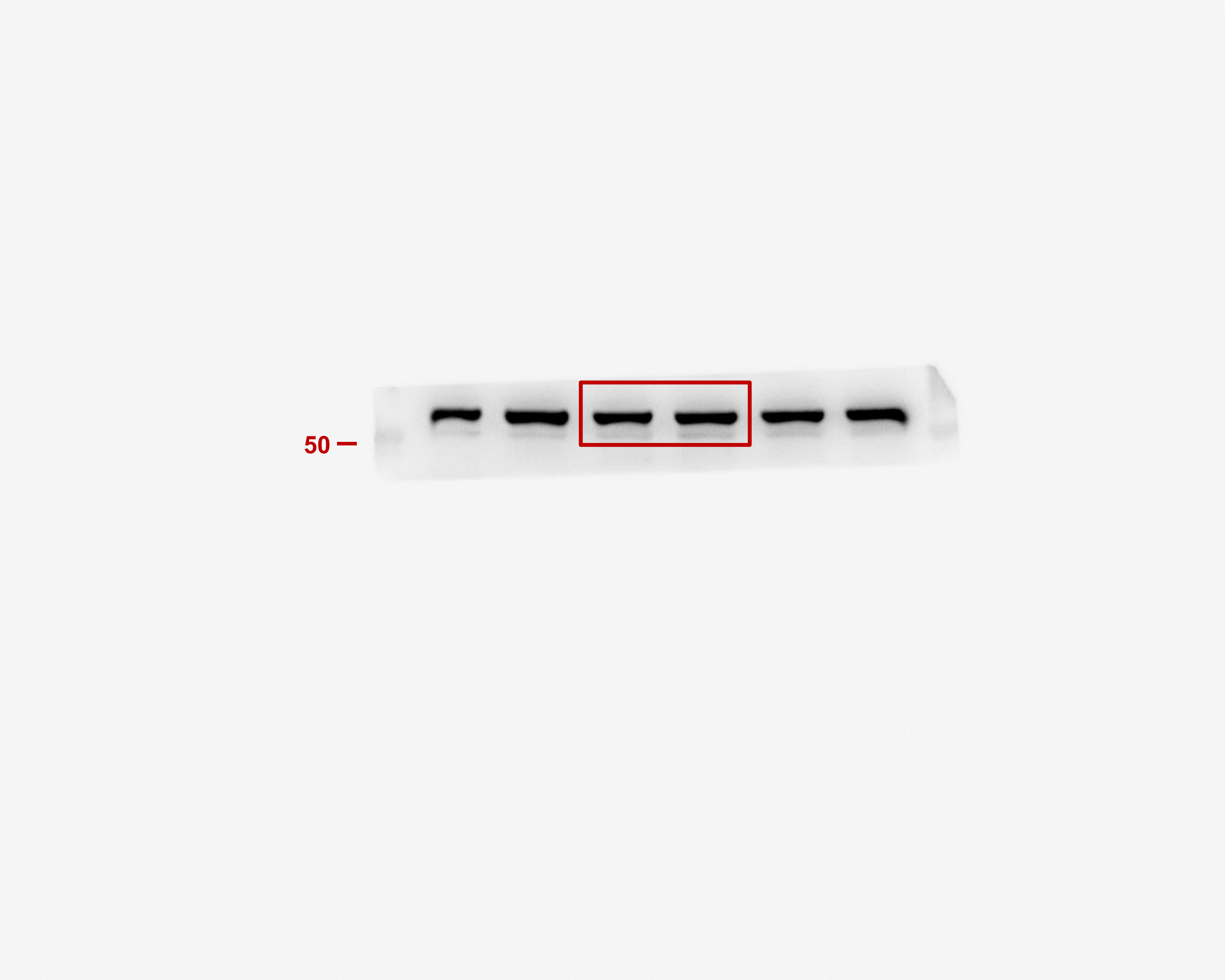

Figure 5C YAP

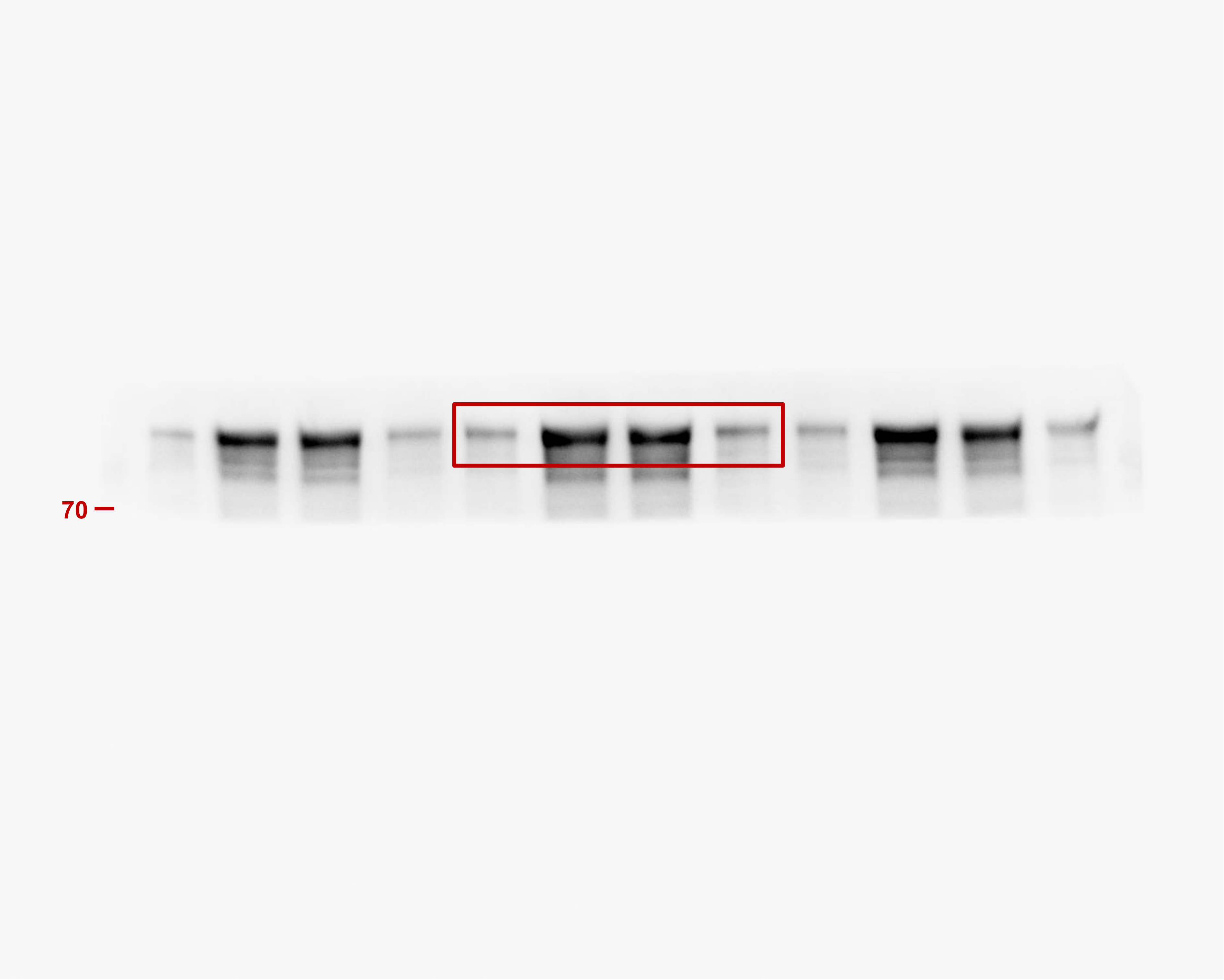

Figure 5C GAPDH

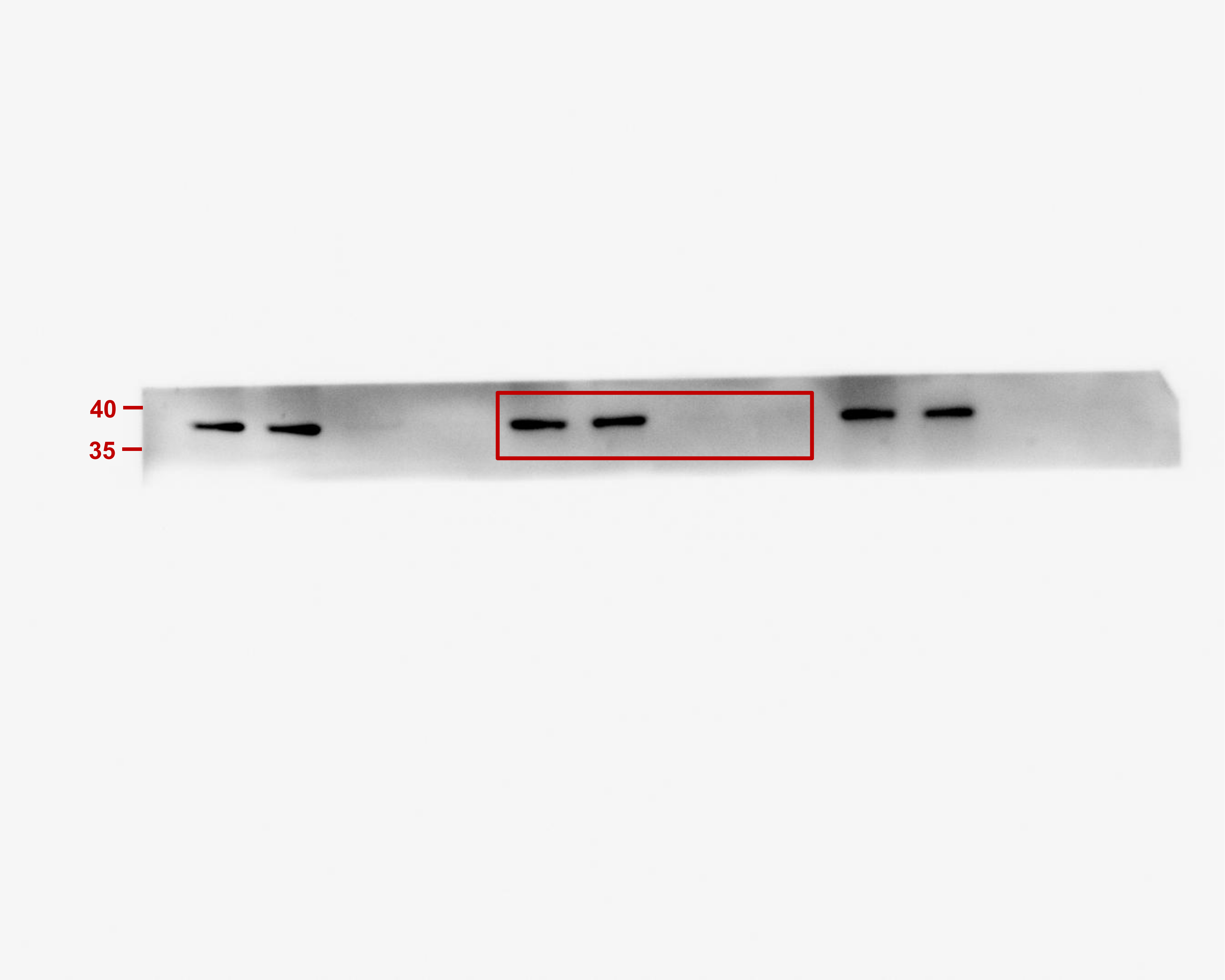

Figure 5C Lamin B

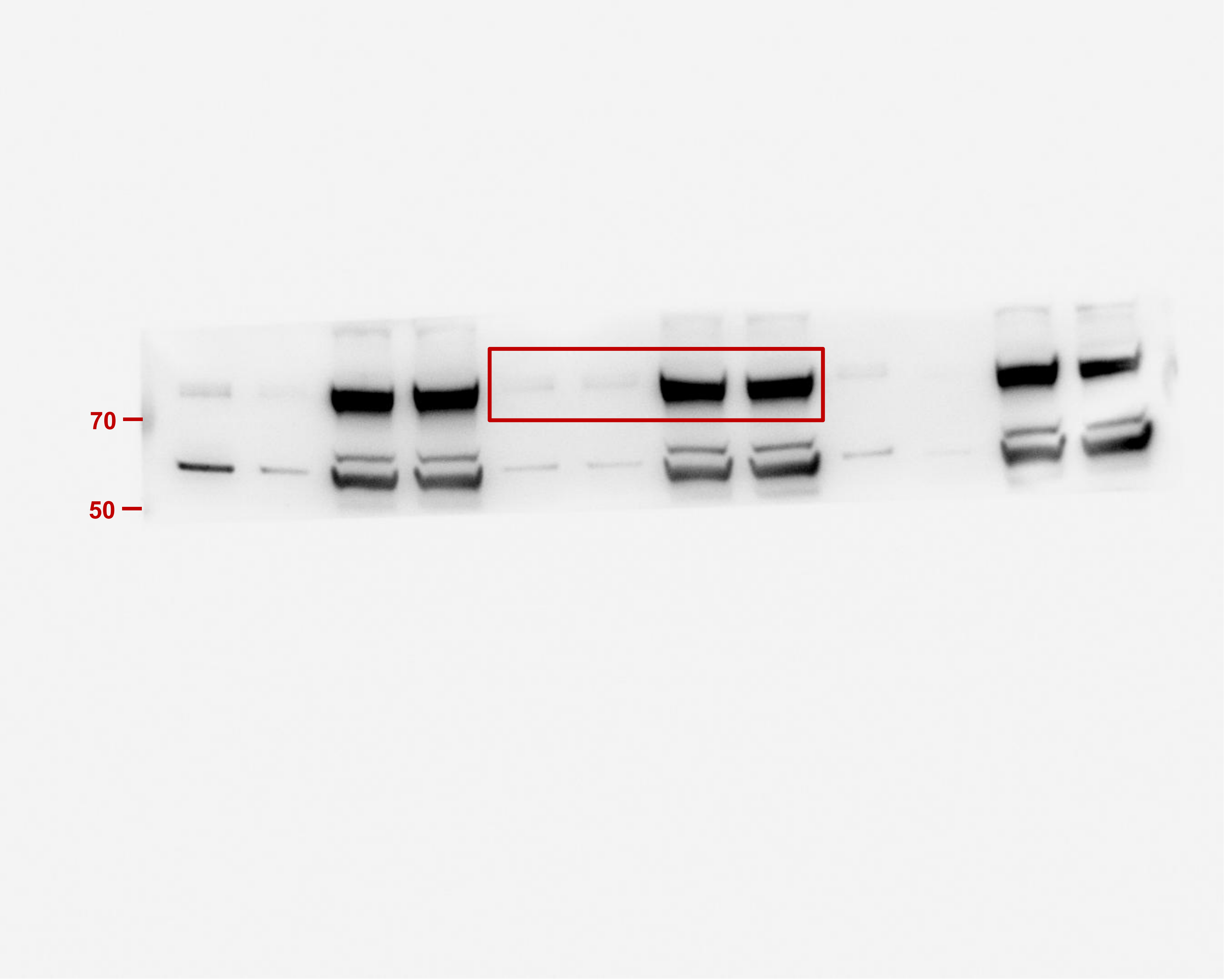

Figure 5F ALDOA

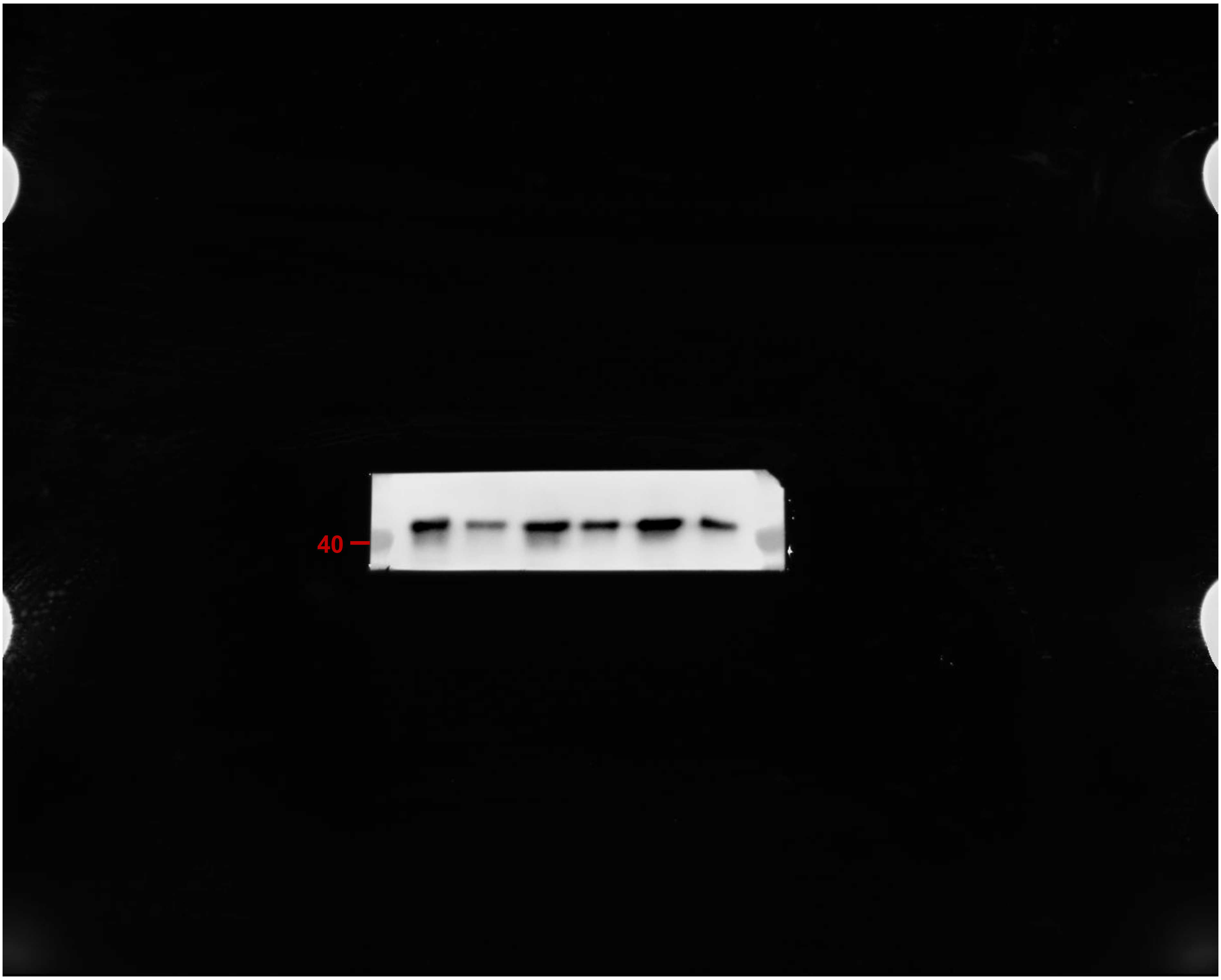

Figure 5F p-YAP

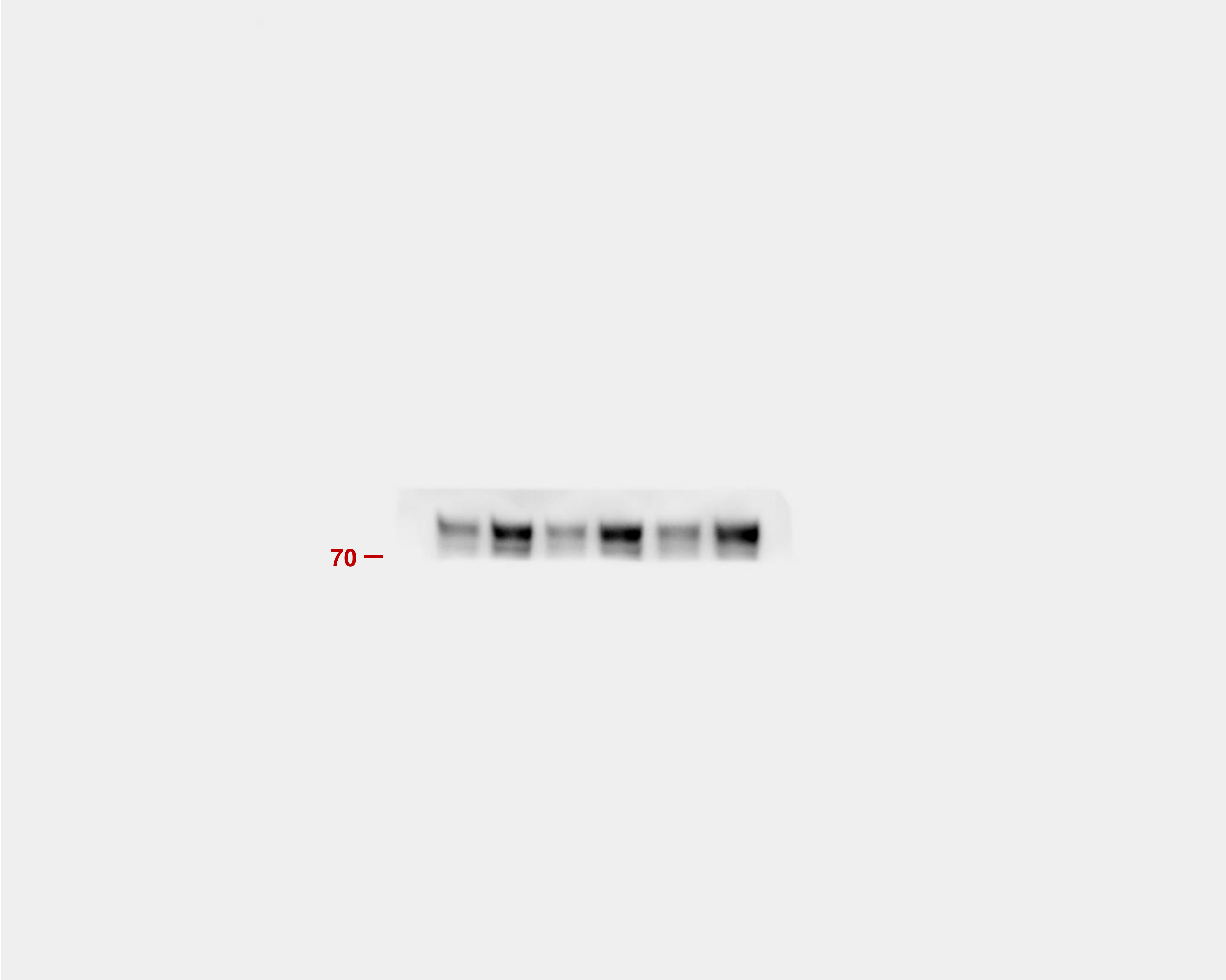

Figure 5F YAP

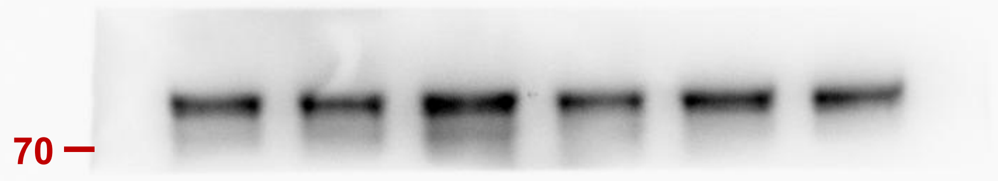

Figure 5F Tubulin

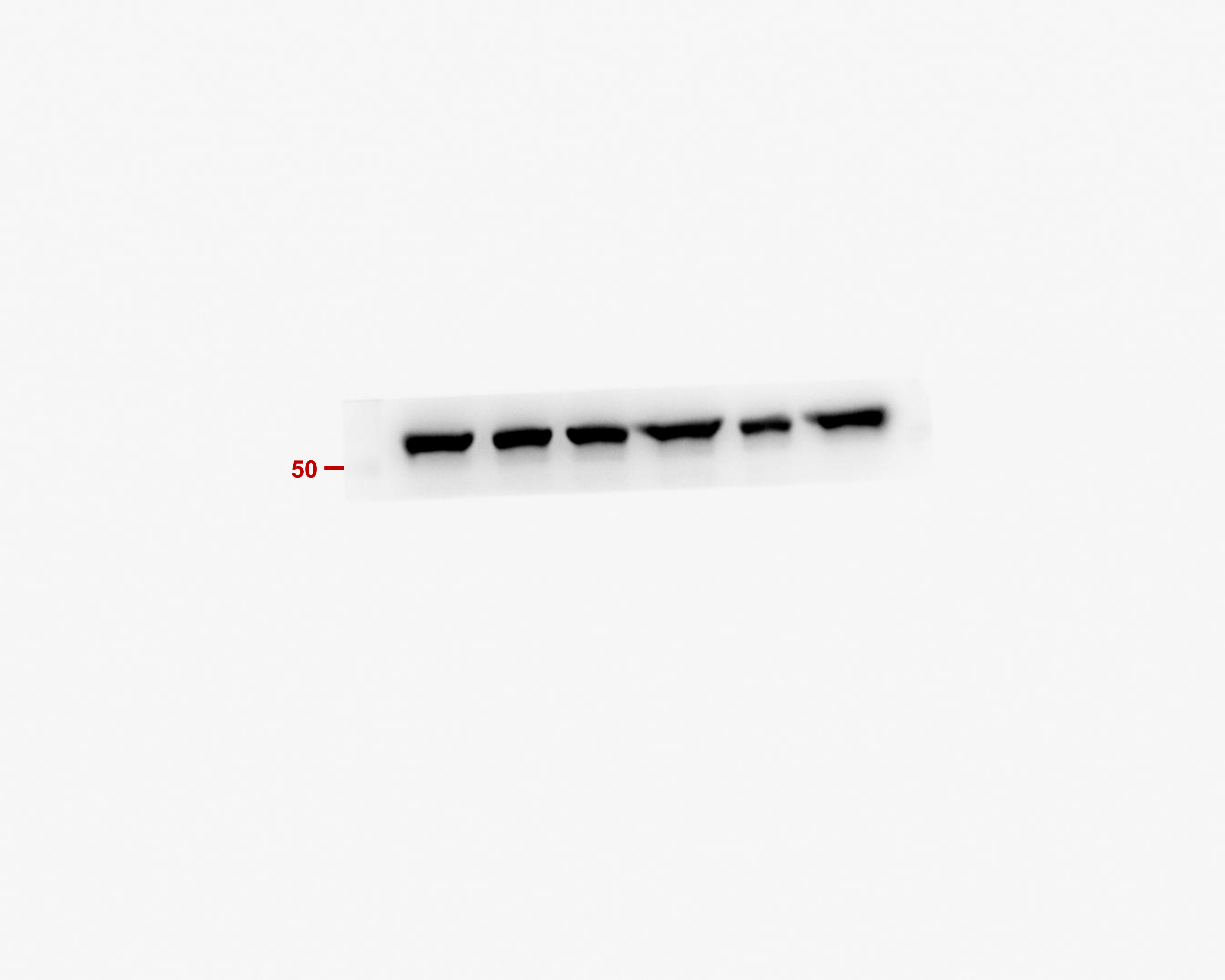

Figure 6A YAP

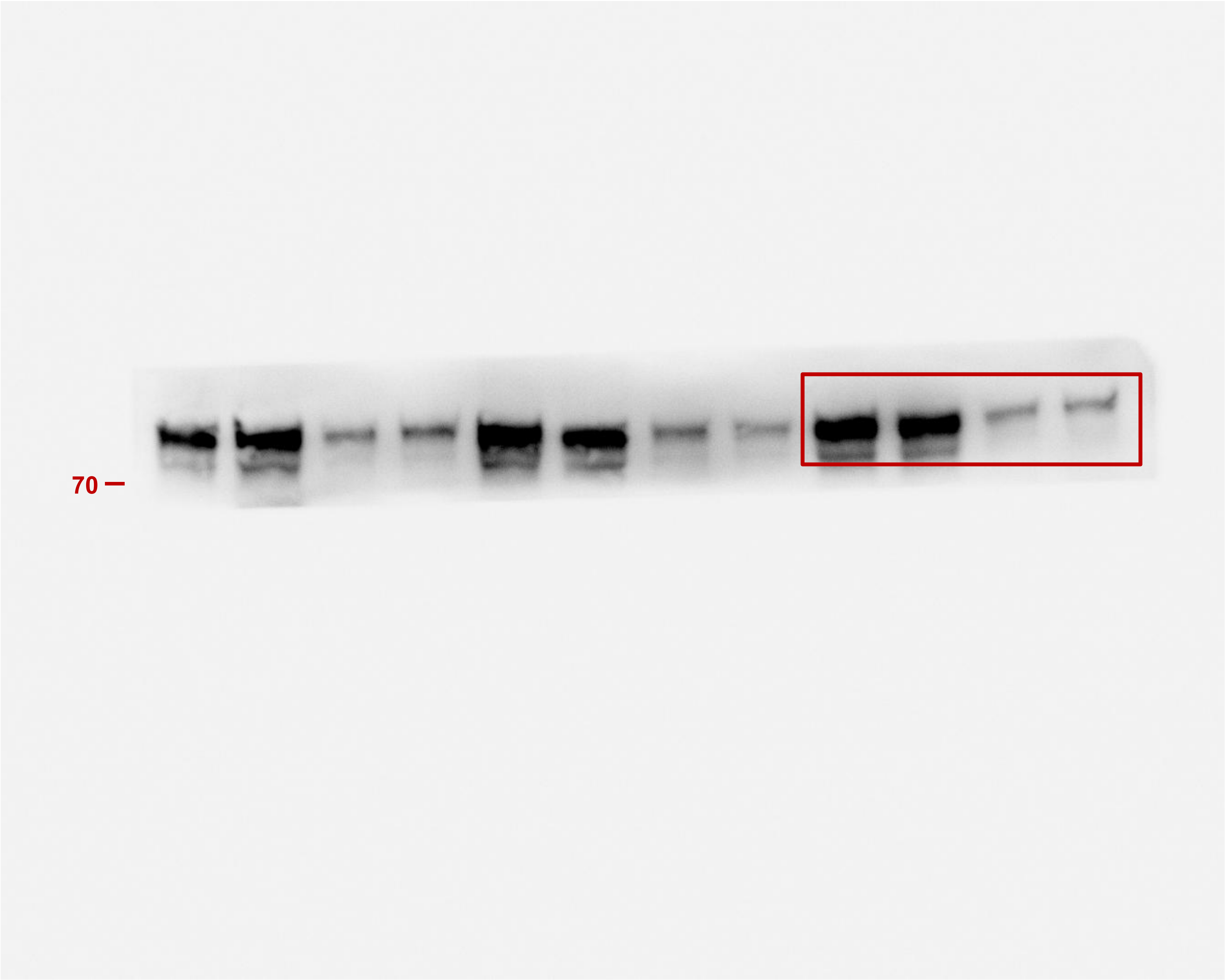

Figure 6A Tubulin

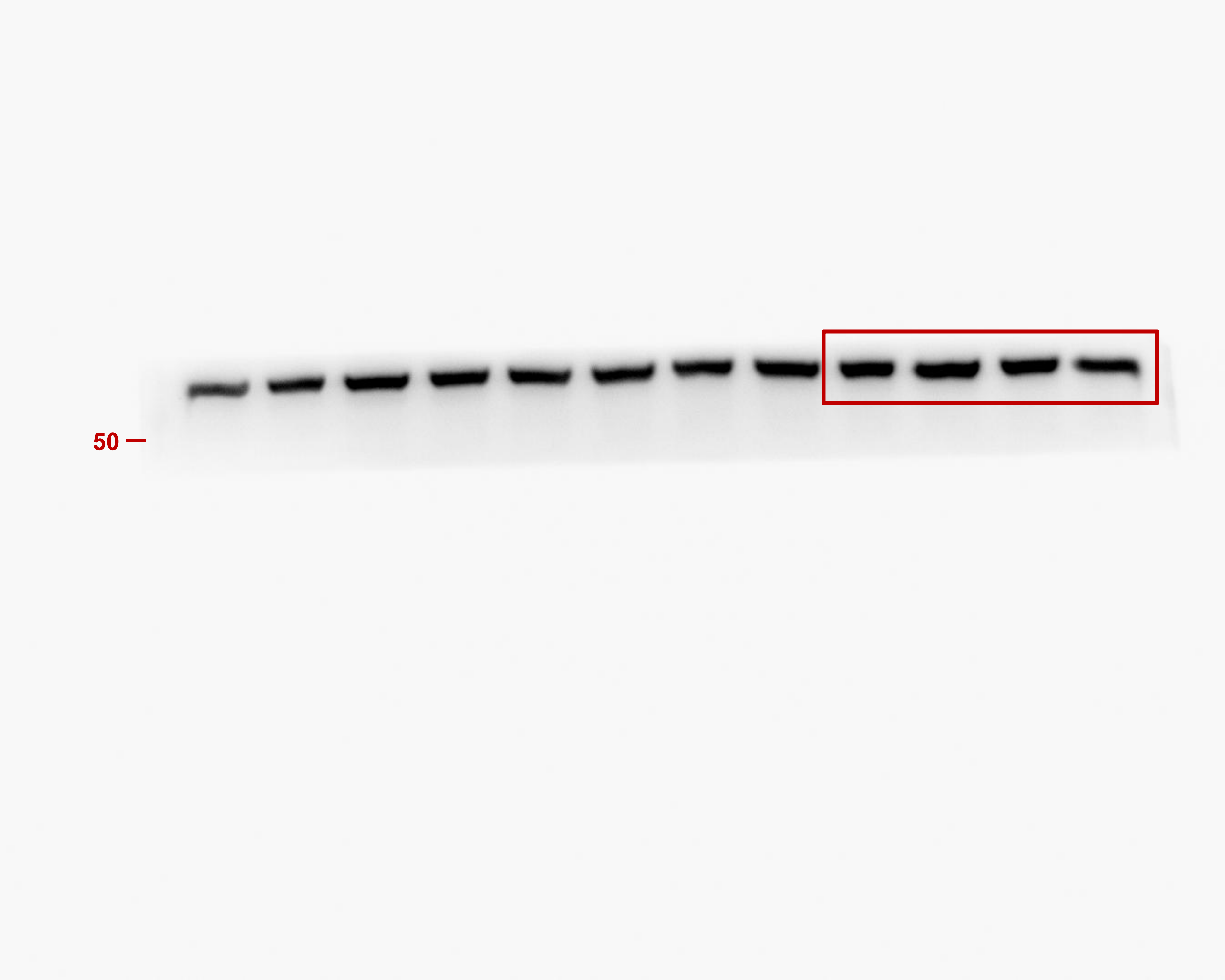

Figure 7A p-AMPK

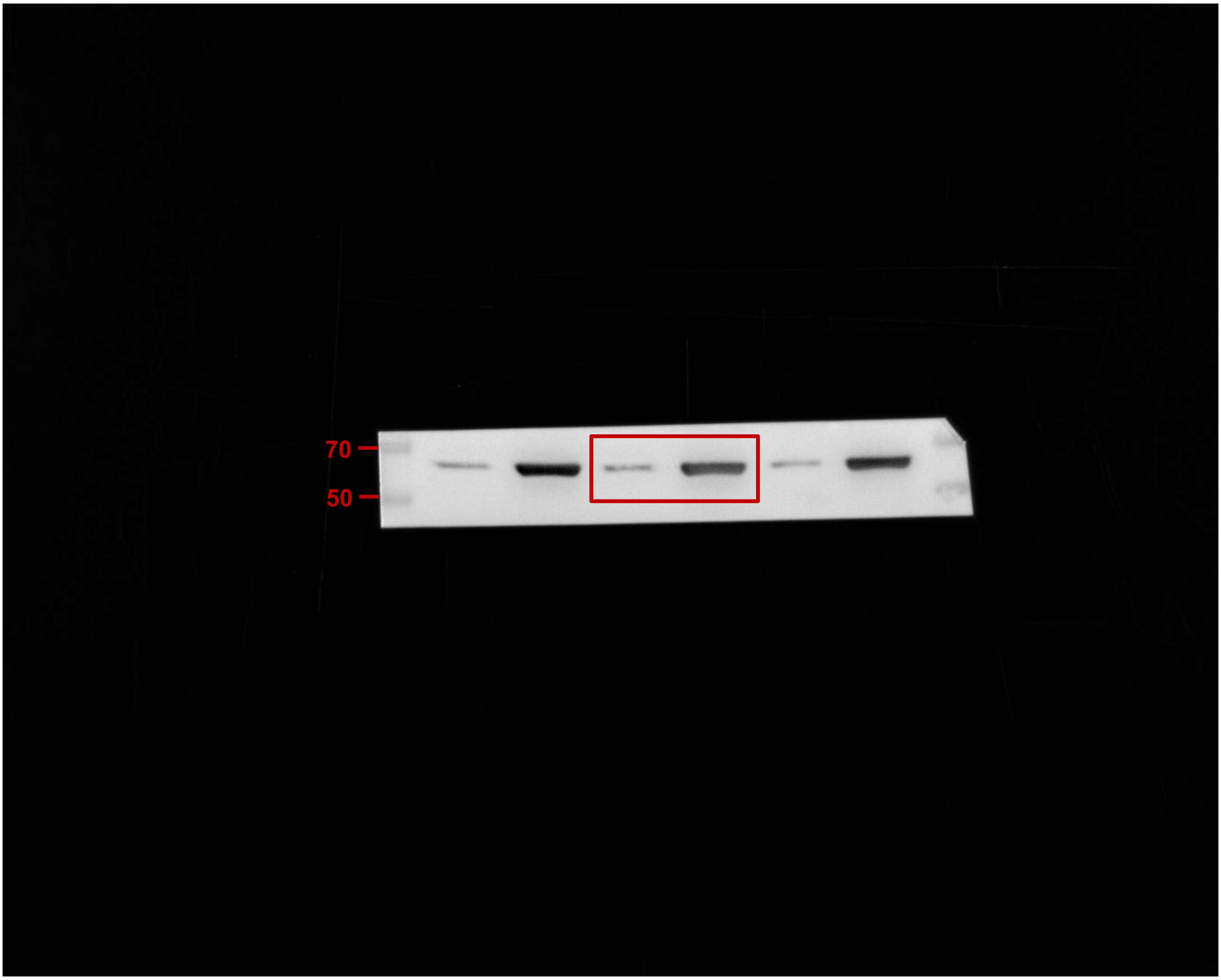

Figure 7A AMPK

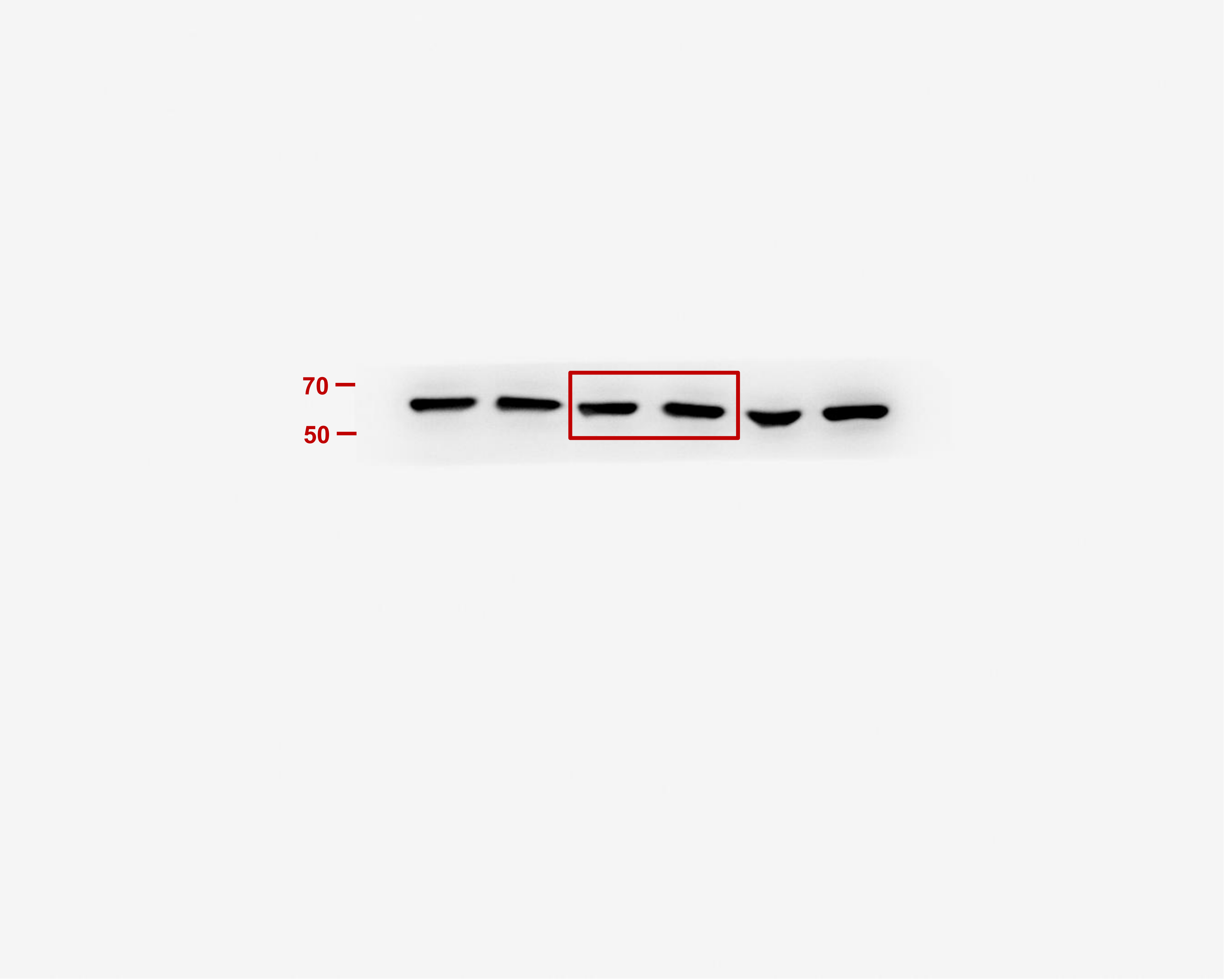

Figure 7A p-AMPK

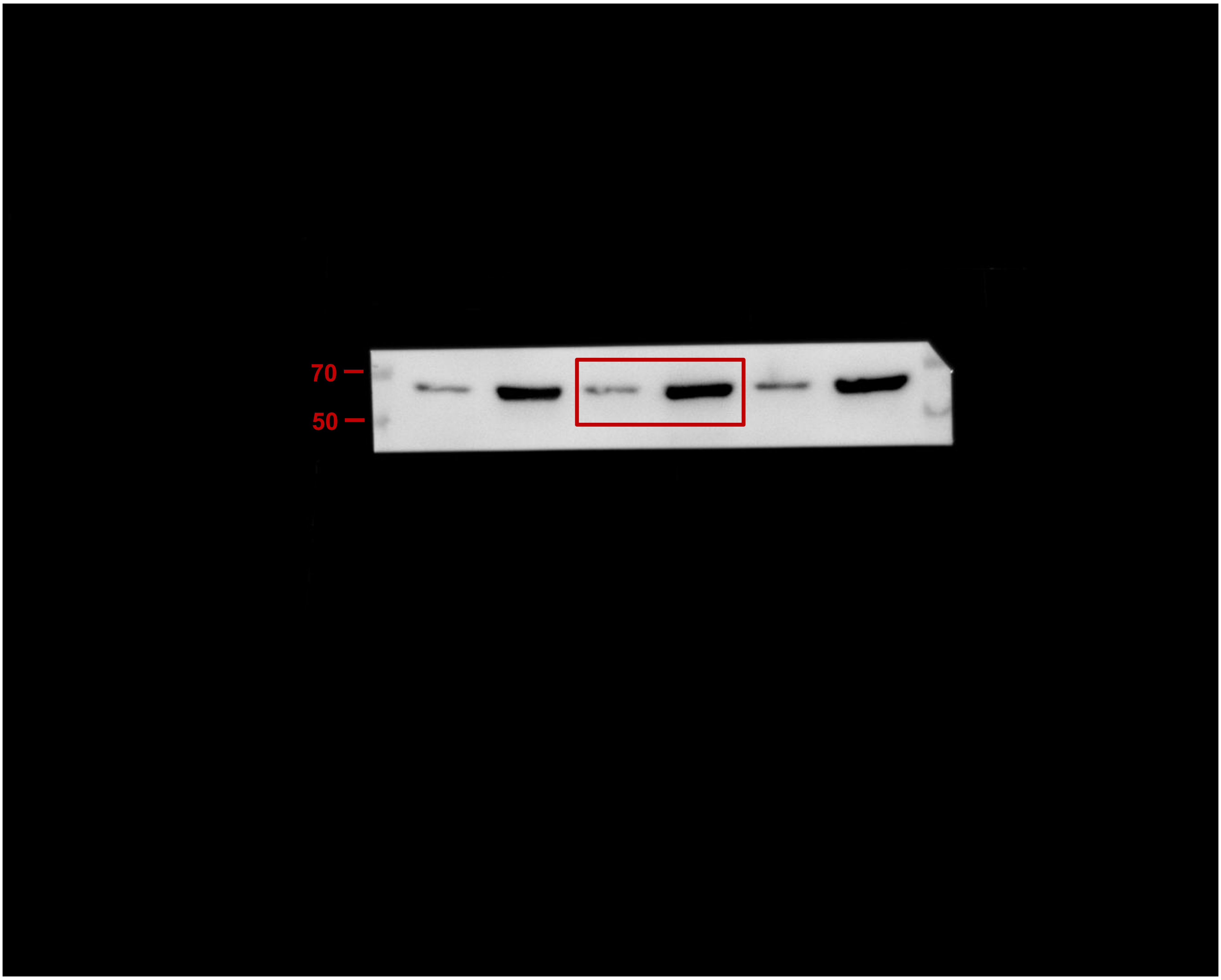

Figure 7A AMPK

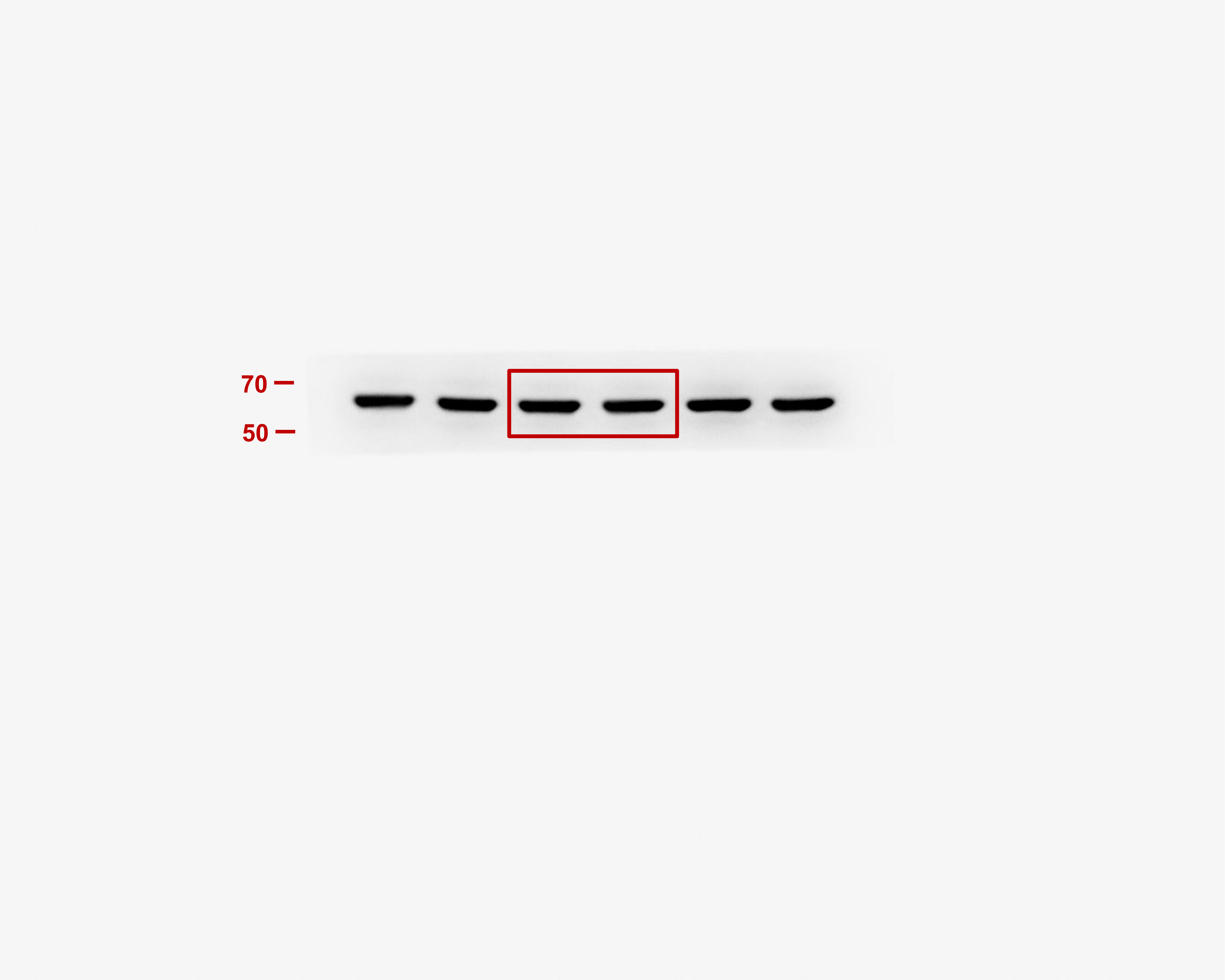

Figure 7B p-AMPK

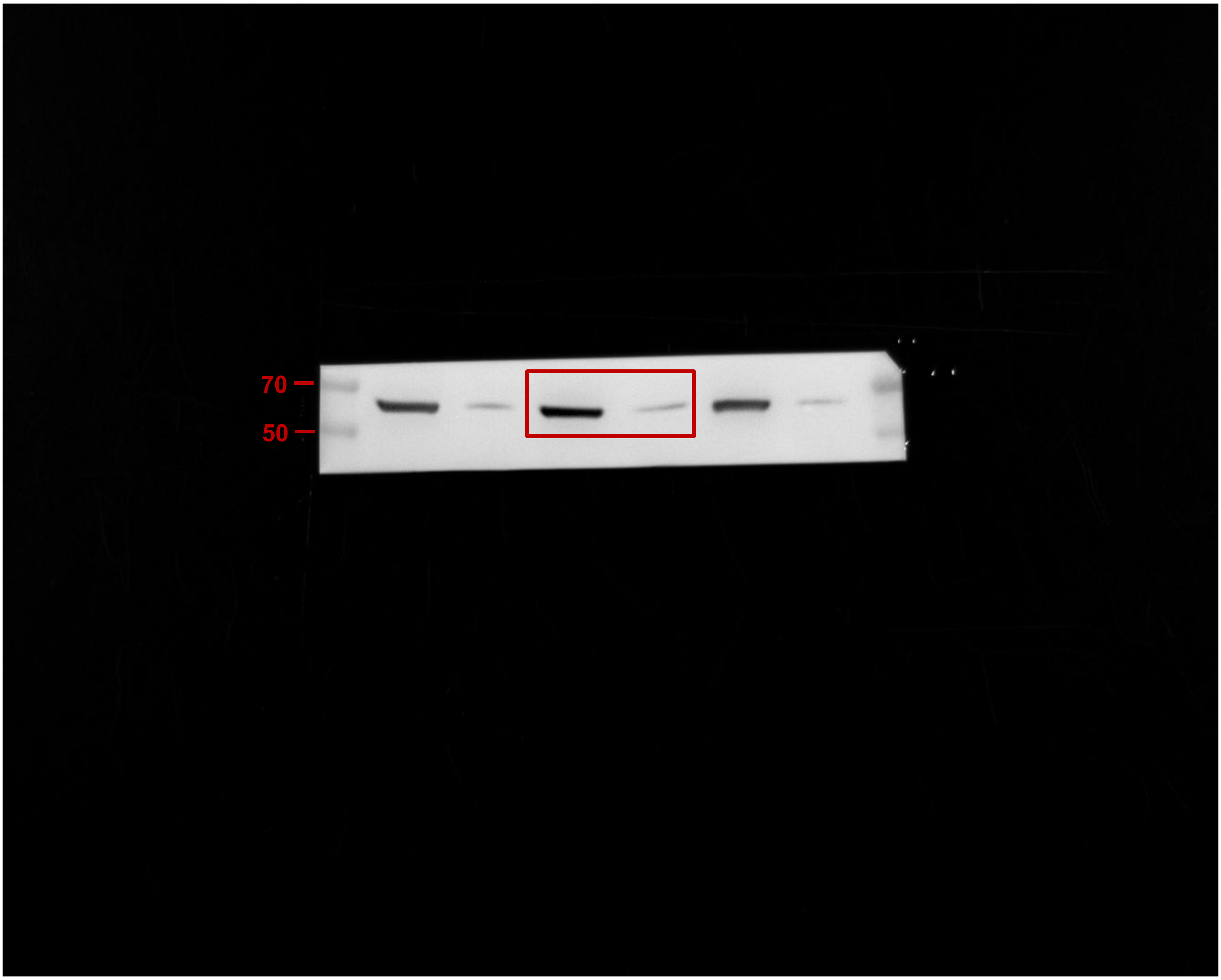

Figure 7B AMPK

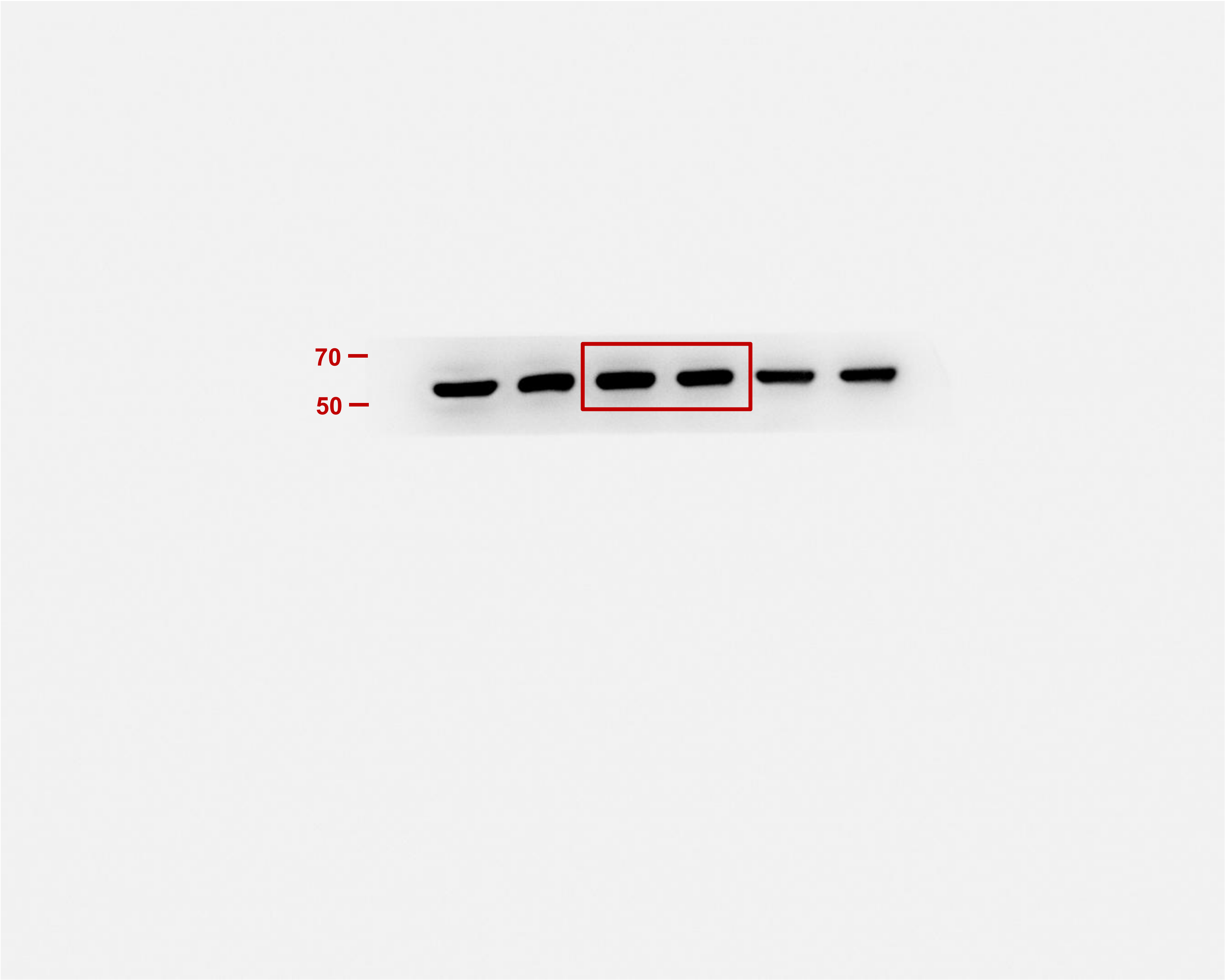

Figure 7B p-AMPK

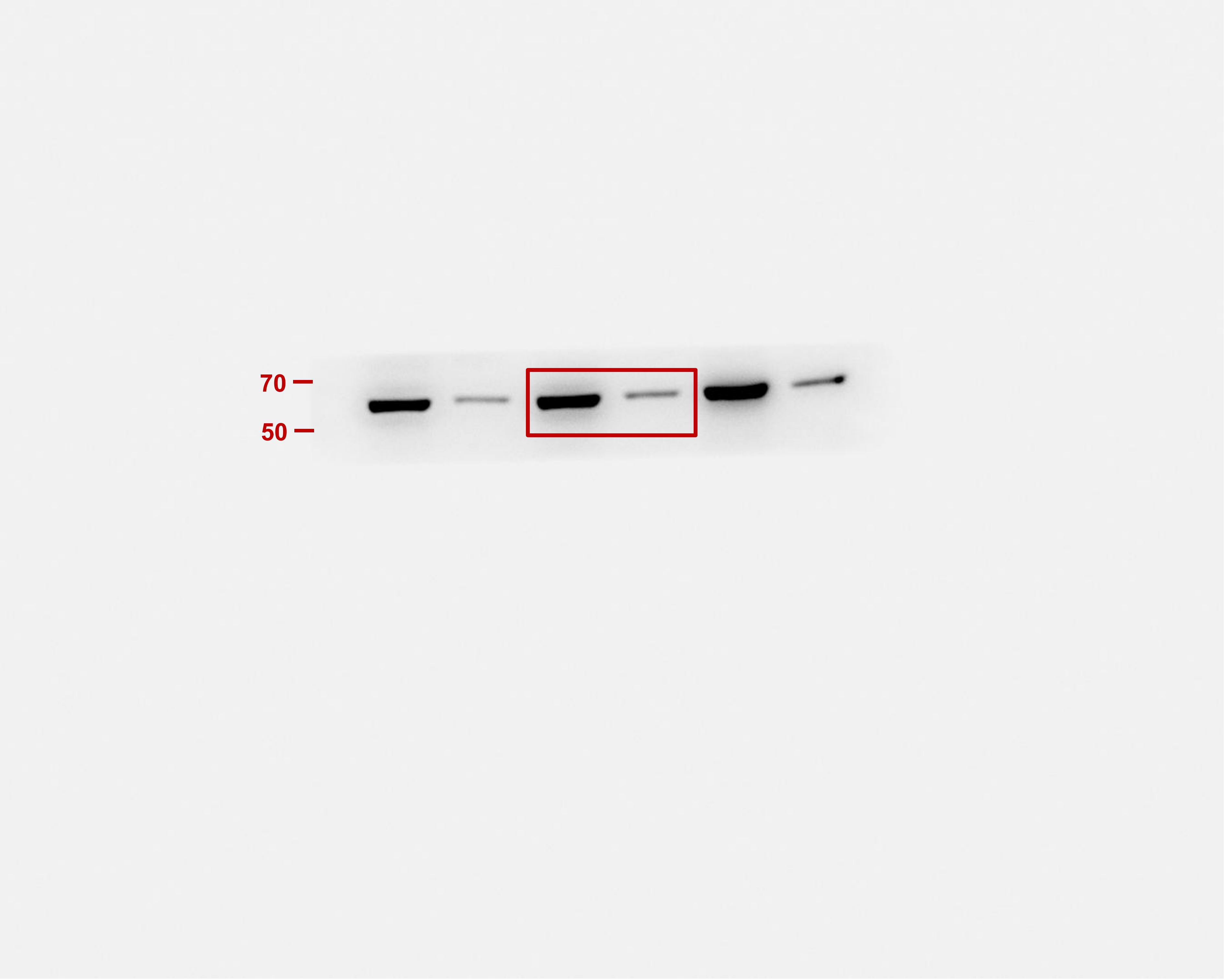

Figure 7B AMPK

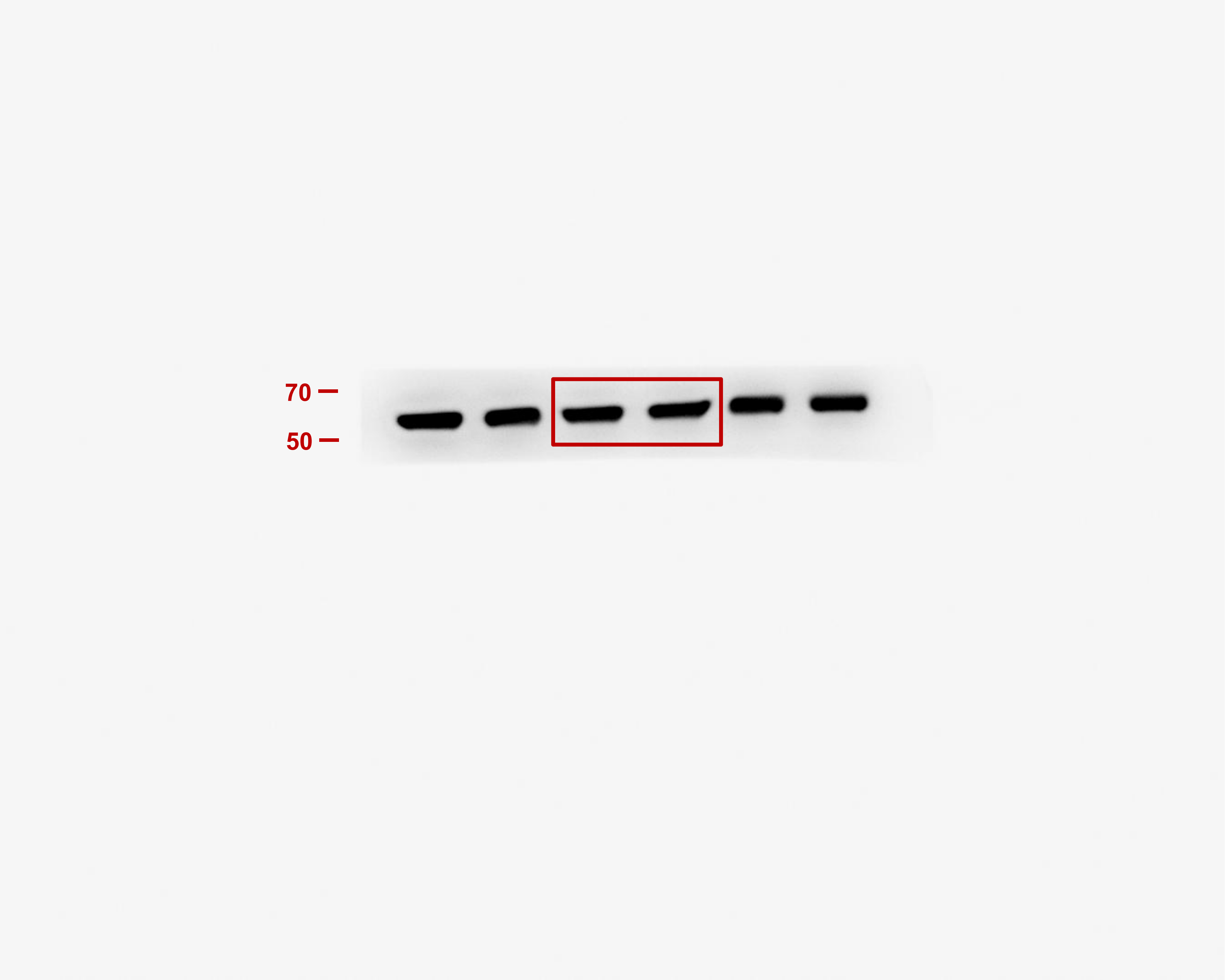

Figure 7A and 7B Tubulin

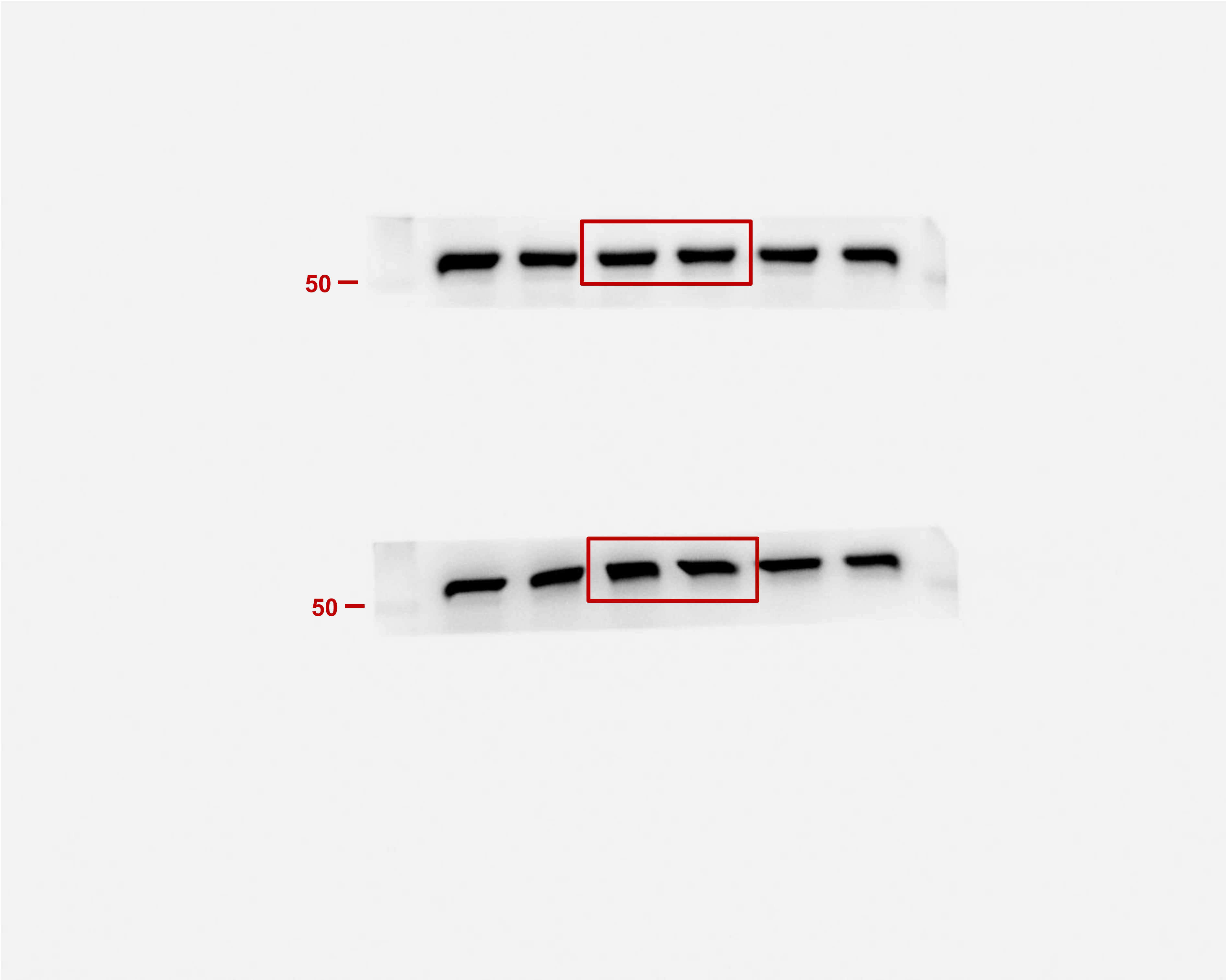

Figure 7A and 7B Tubulin

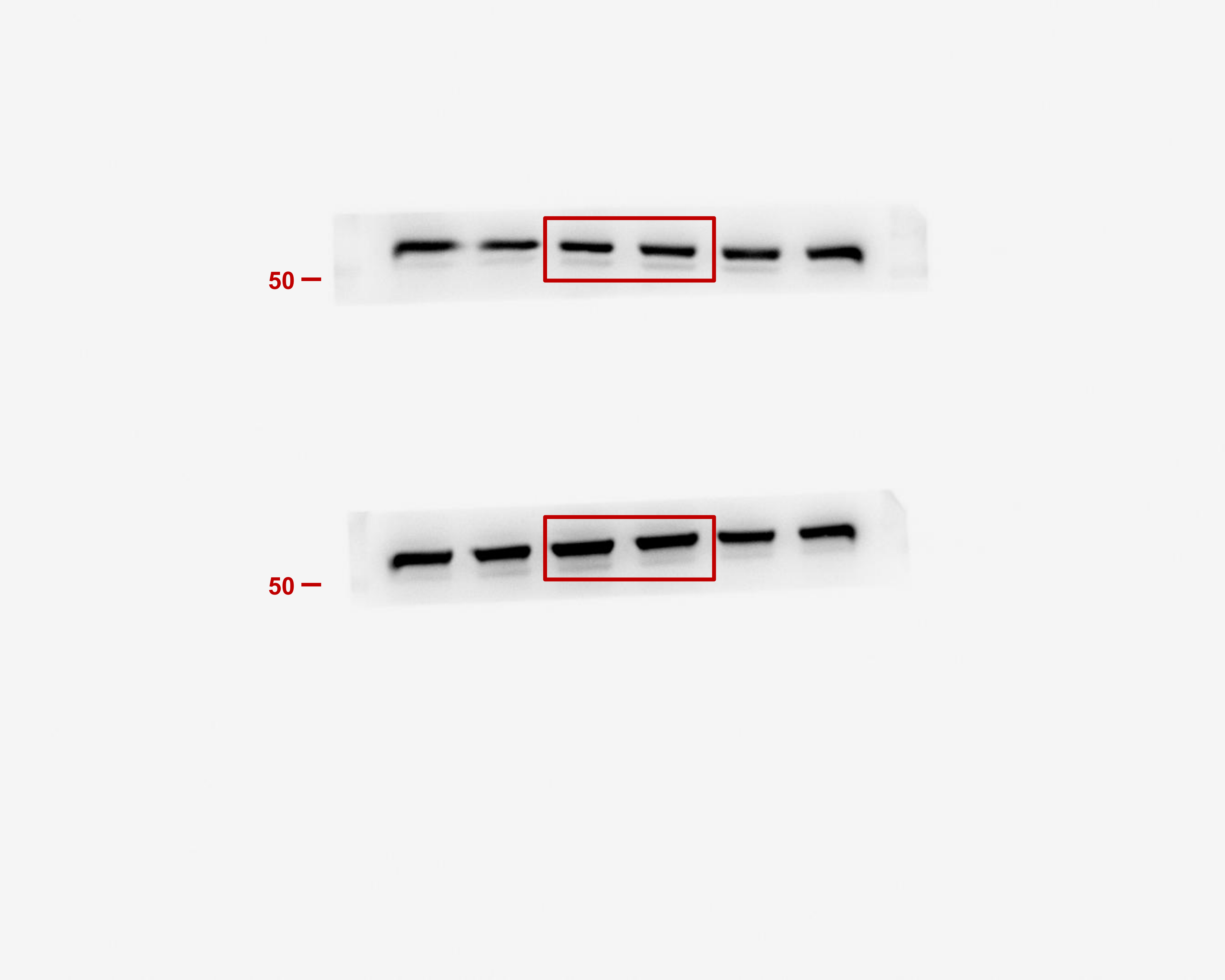

Figure 7C AMPK

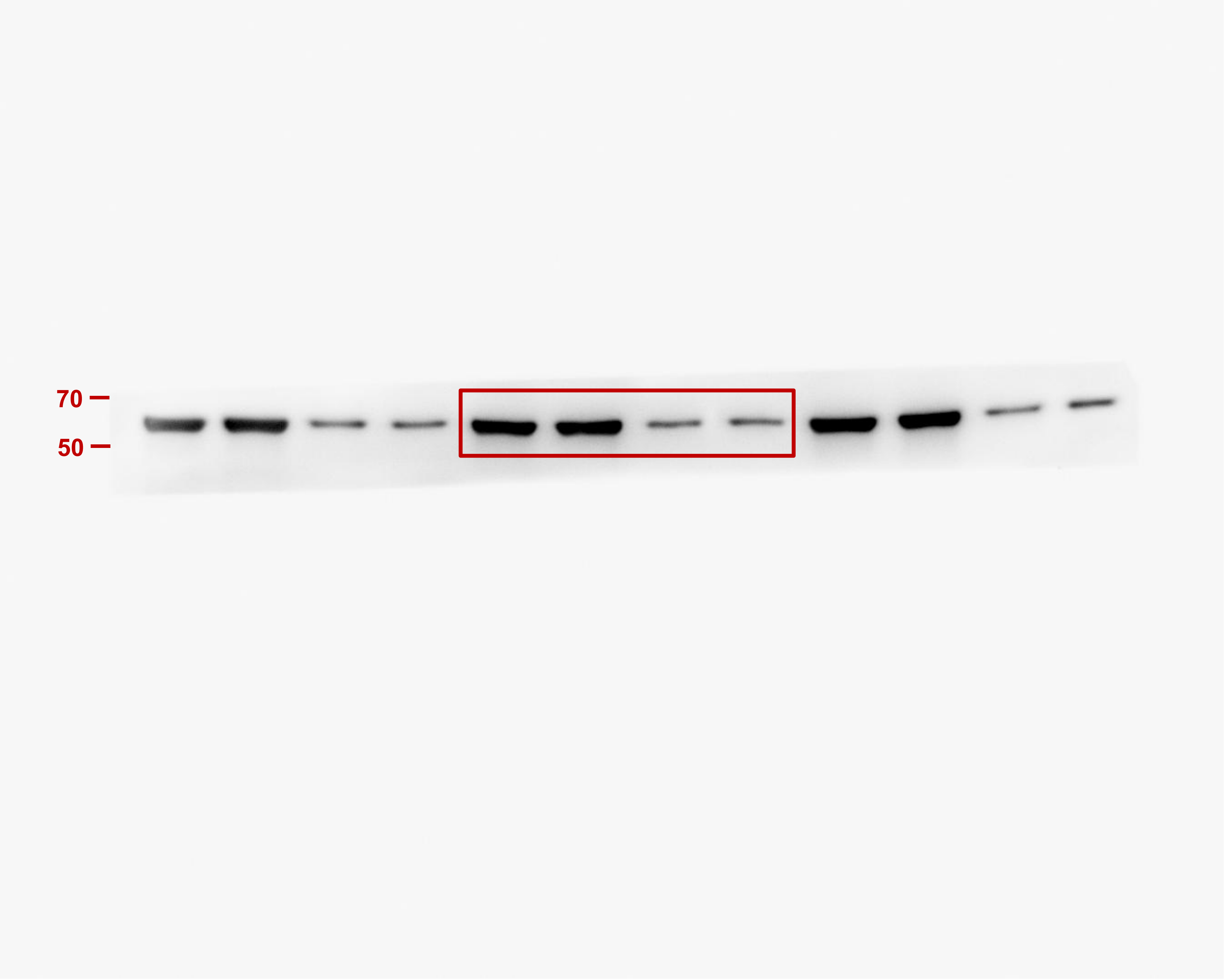

Figure 7C p-YAP

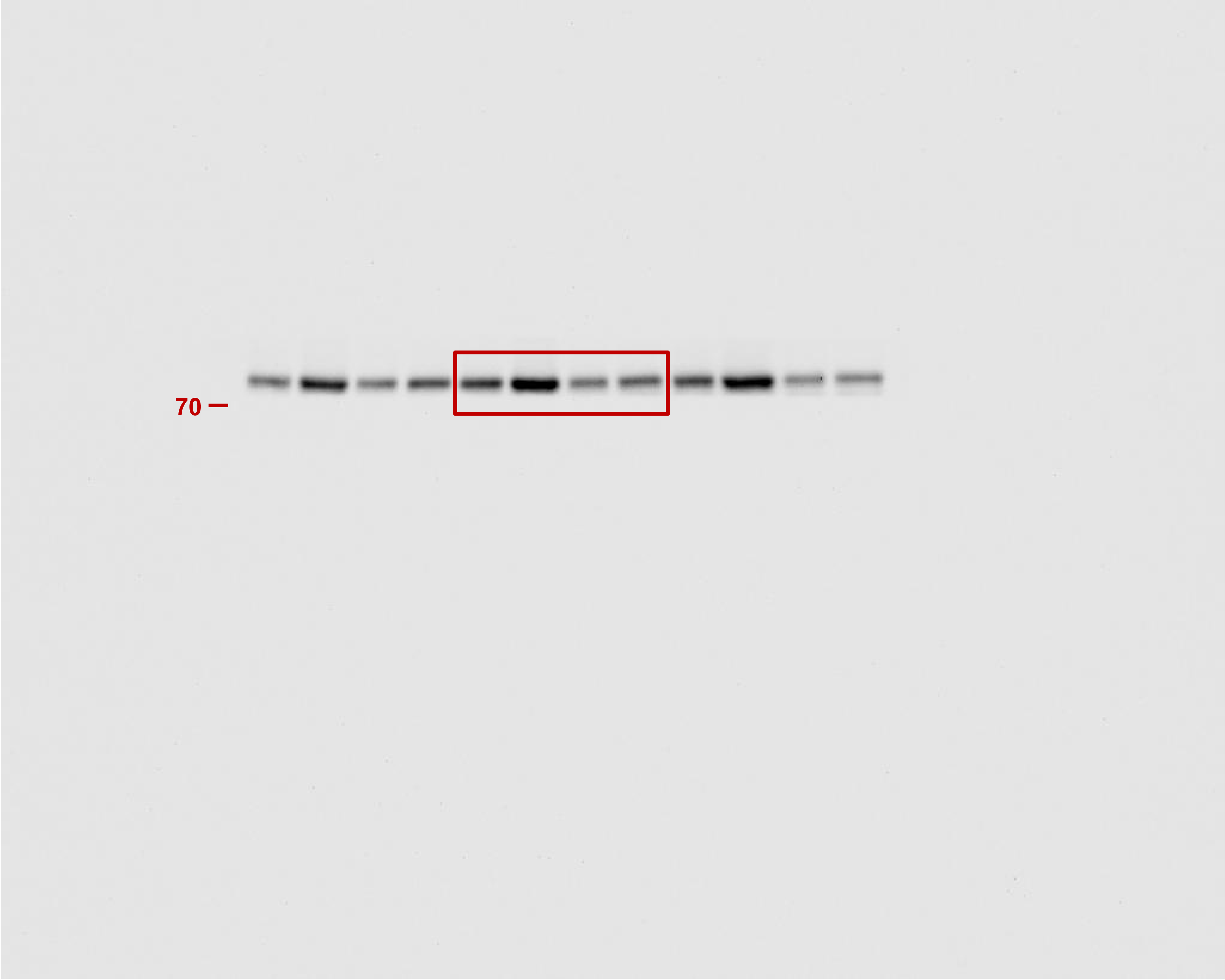

Figure 7C YAP

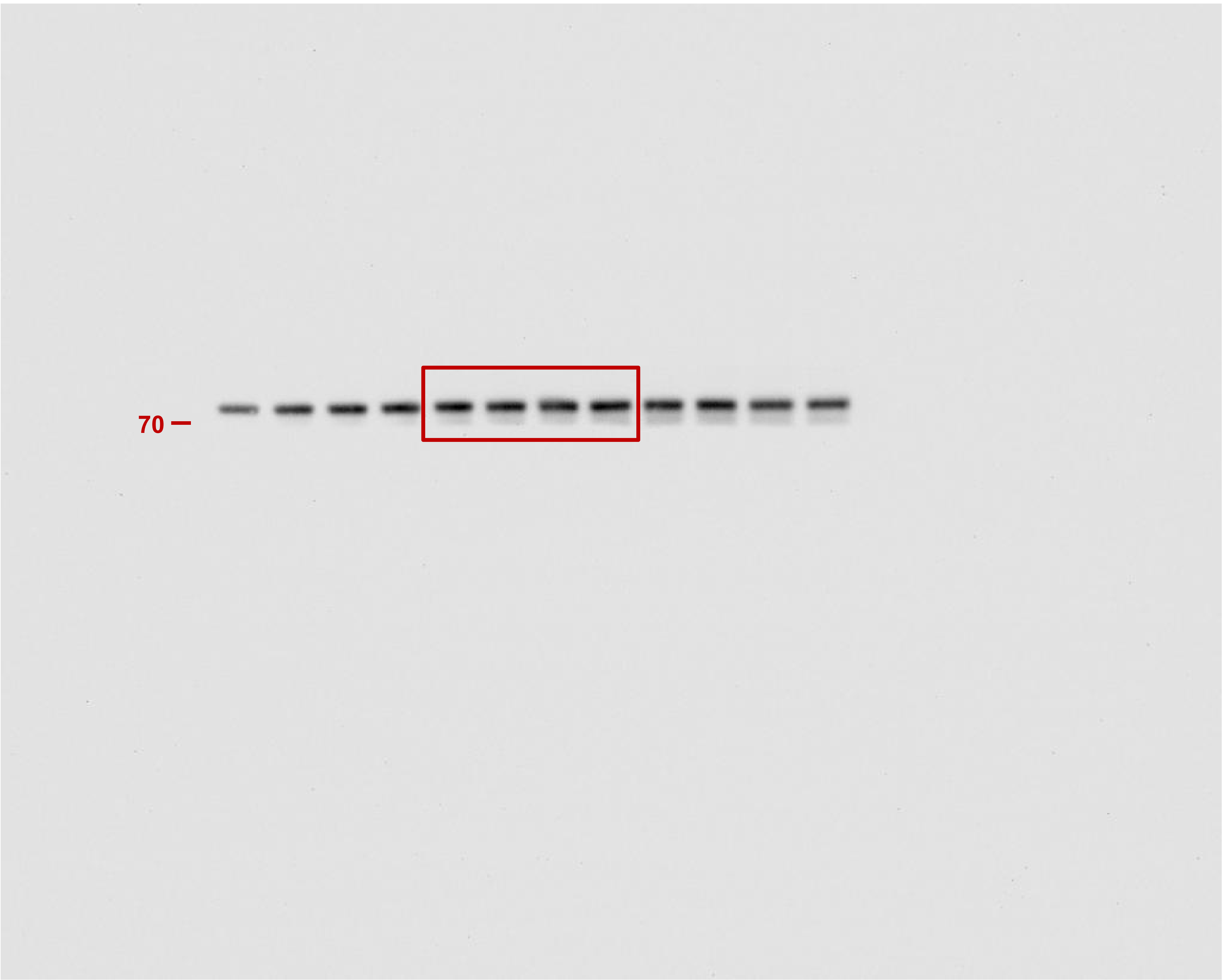

Figure 7C Tubulin

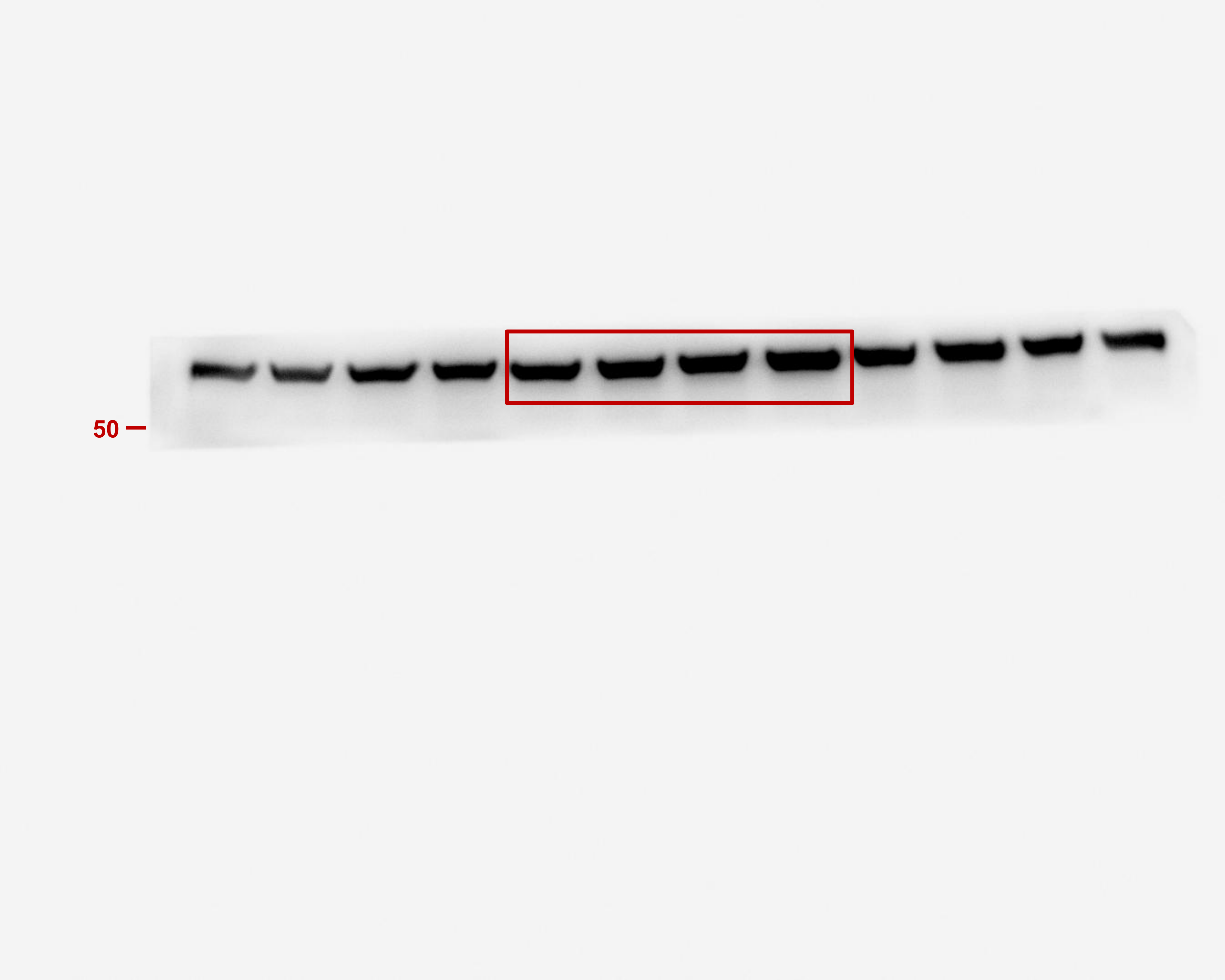

Figure S5A ALDOA

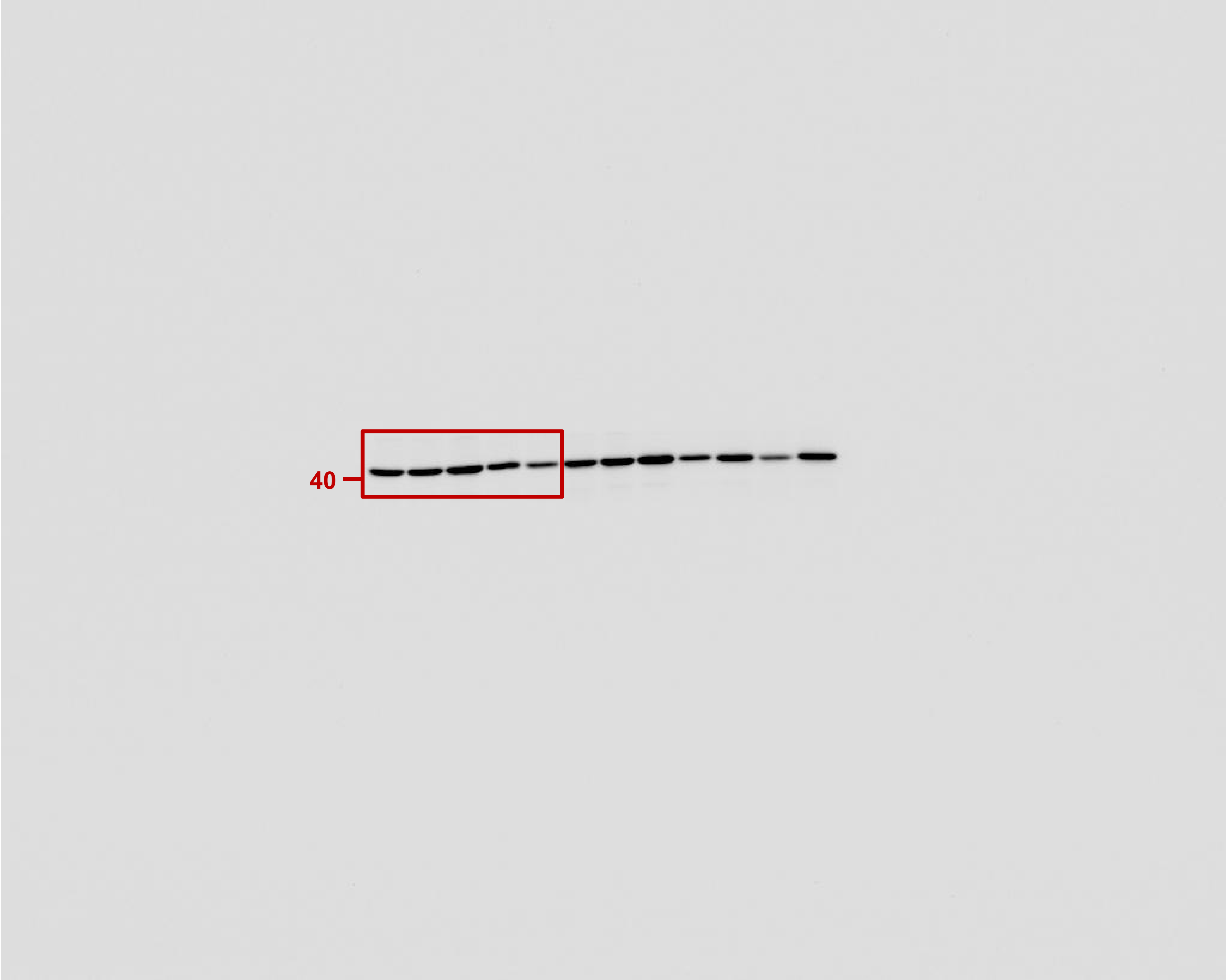

Figure S5A Tubulin

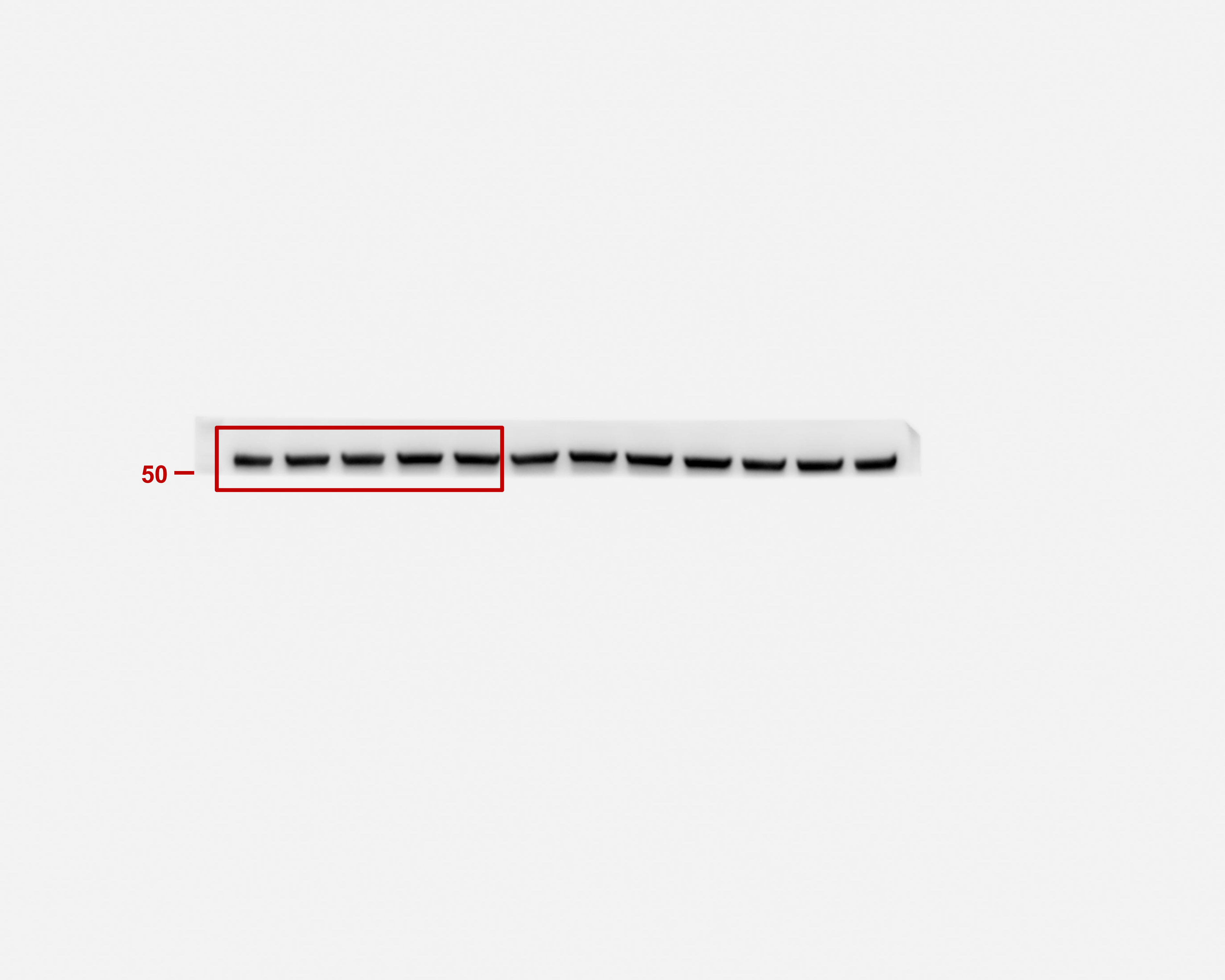

Figure S5C ALDOA

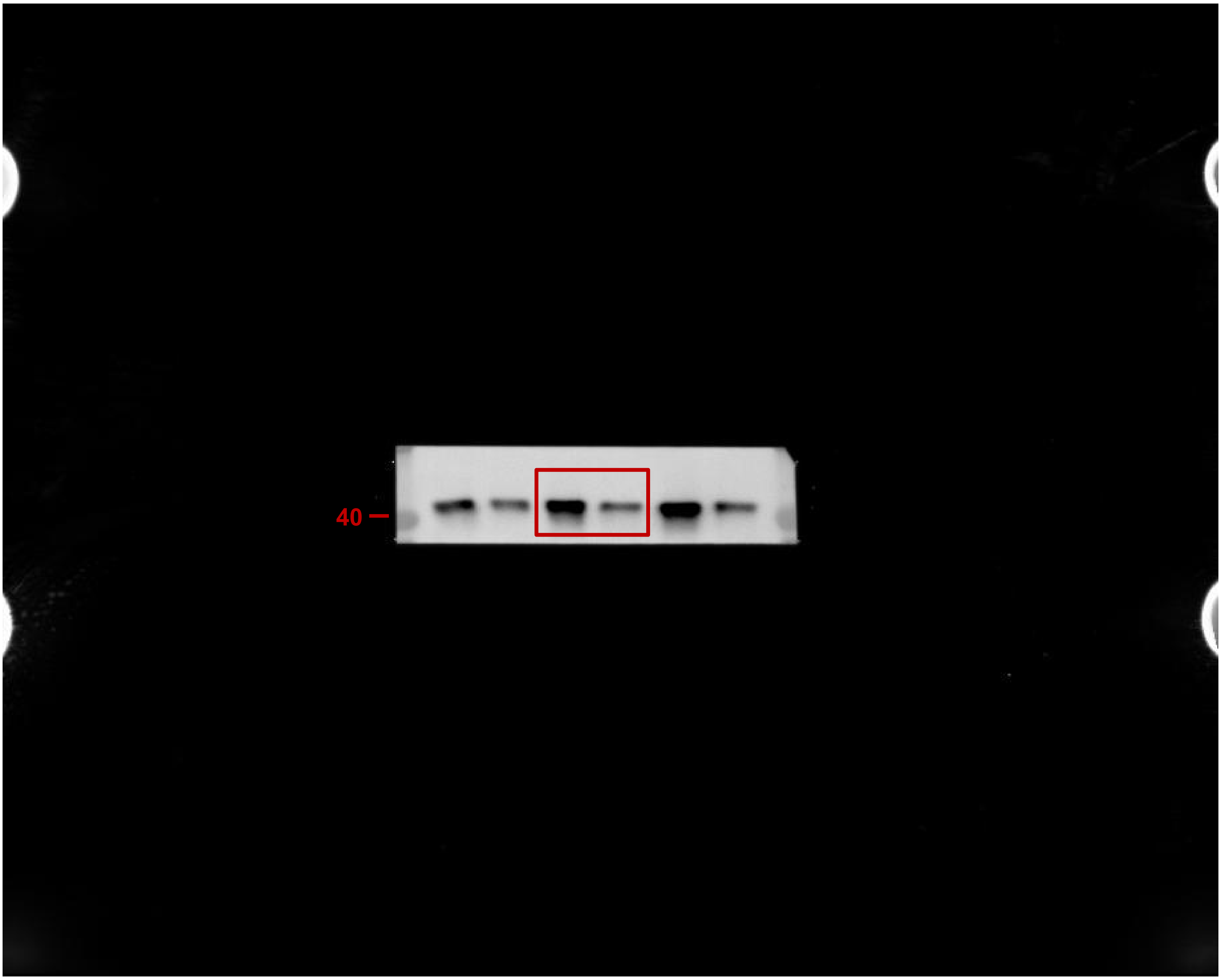

Figure S5C ALDOA

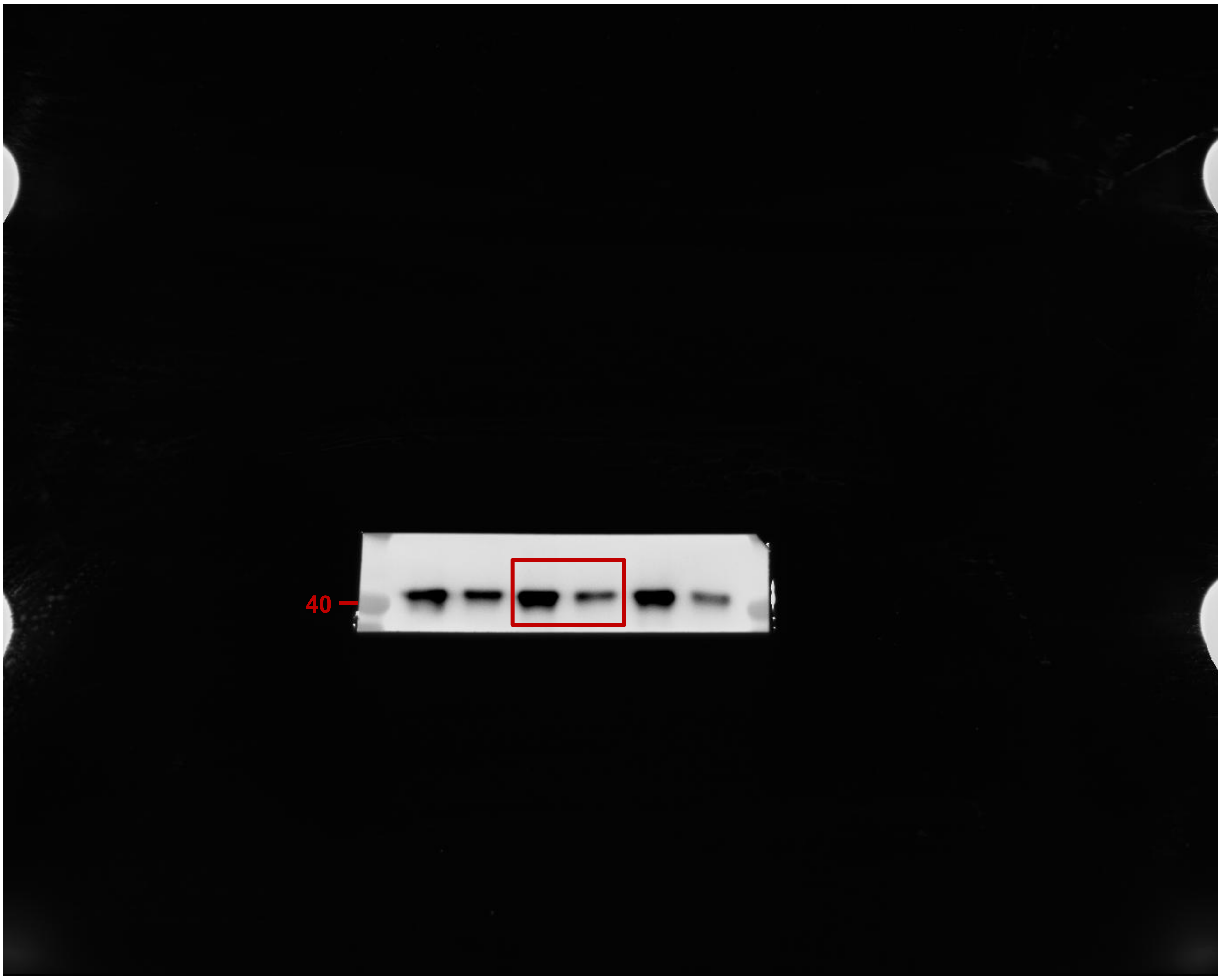

Figure S5C Tubulin

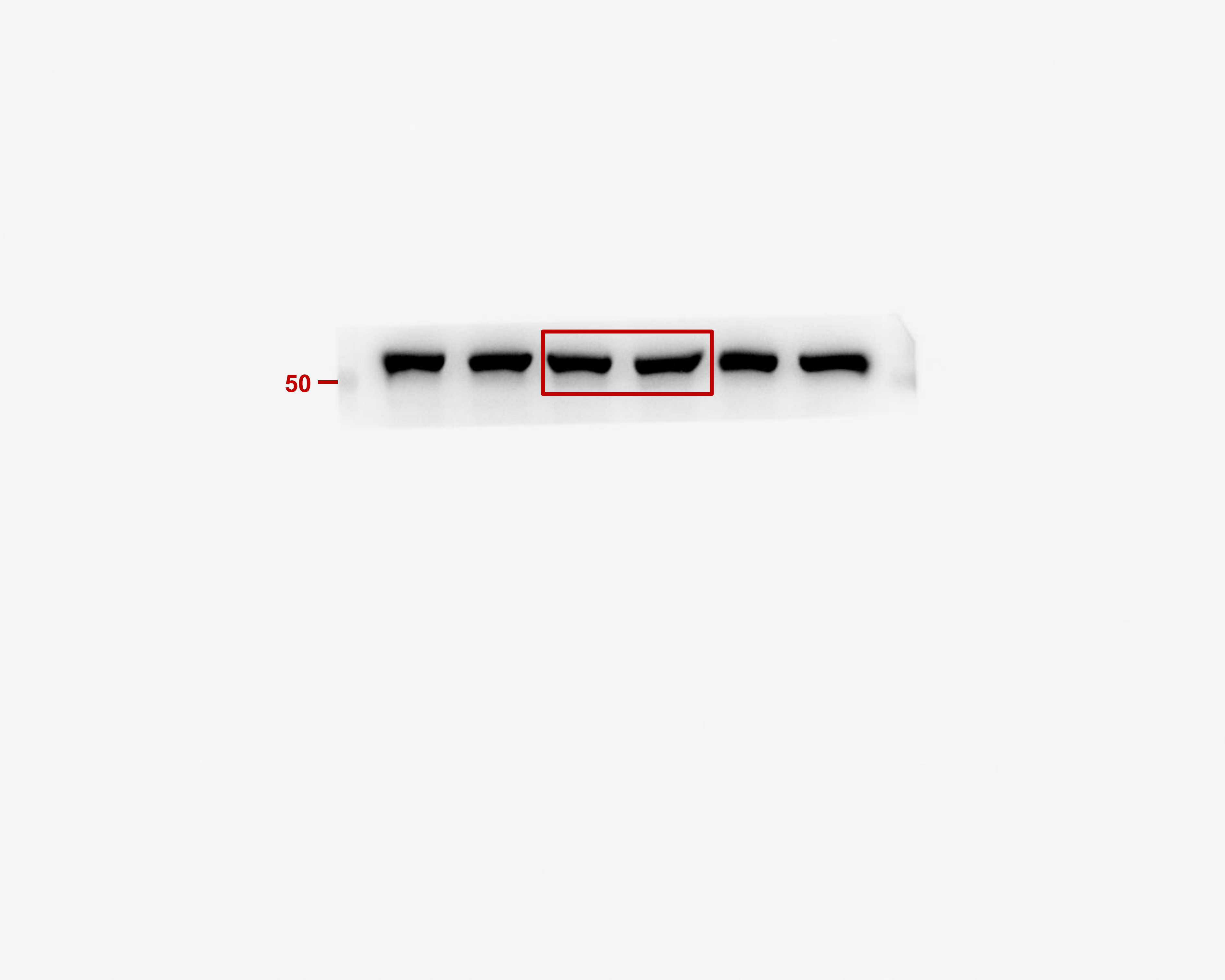

Figure S5C Tubulin

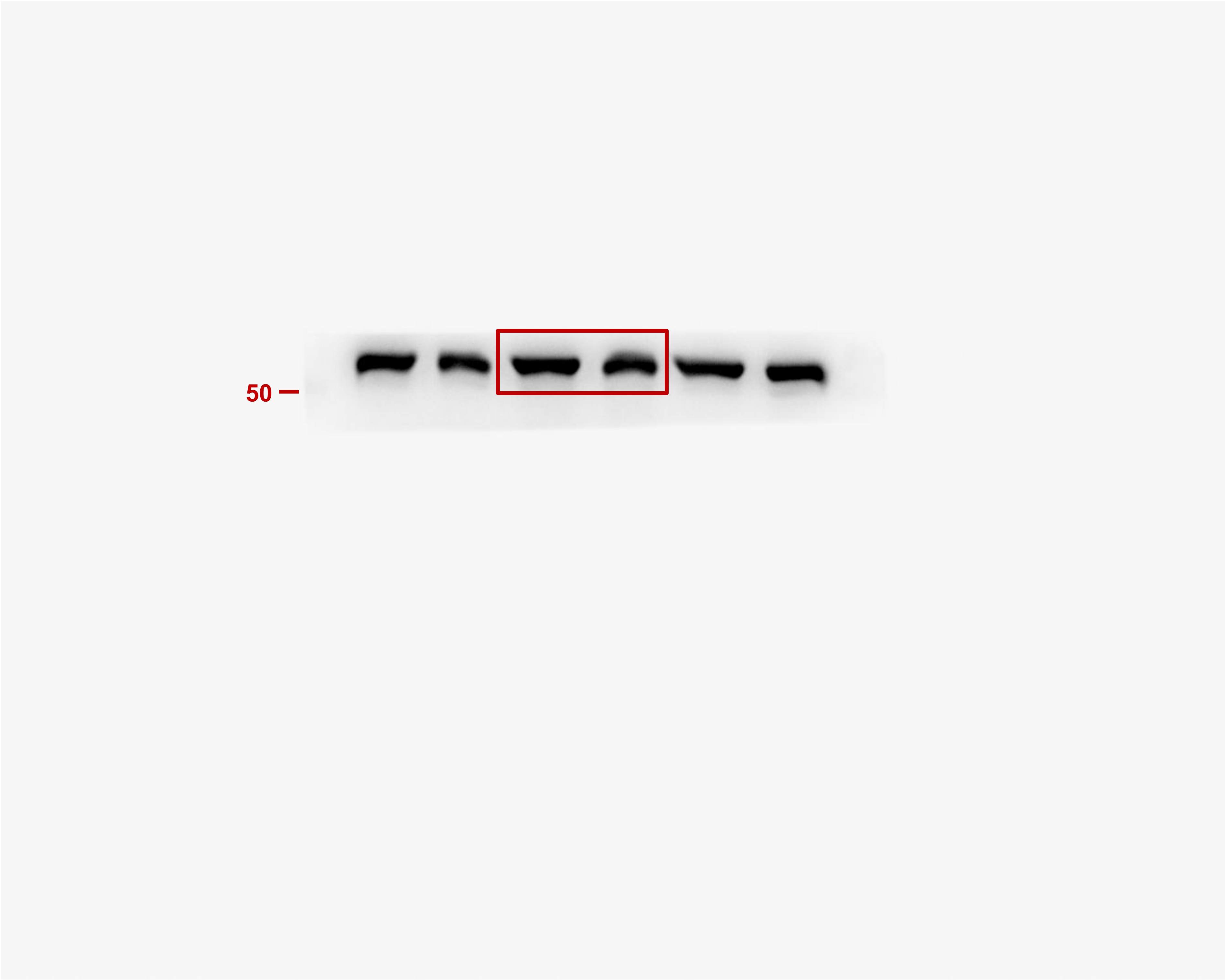

Figure S5E ALDOA

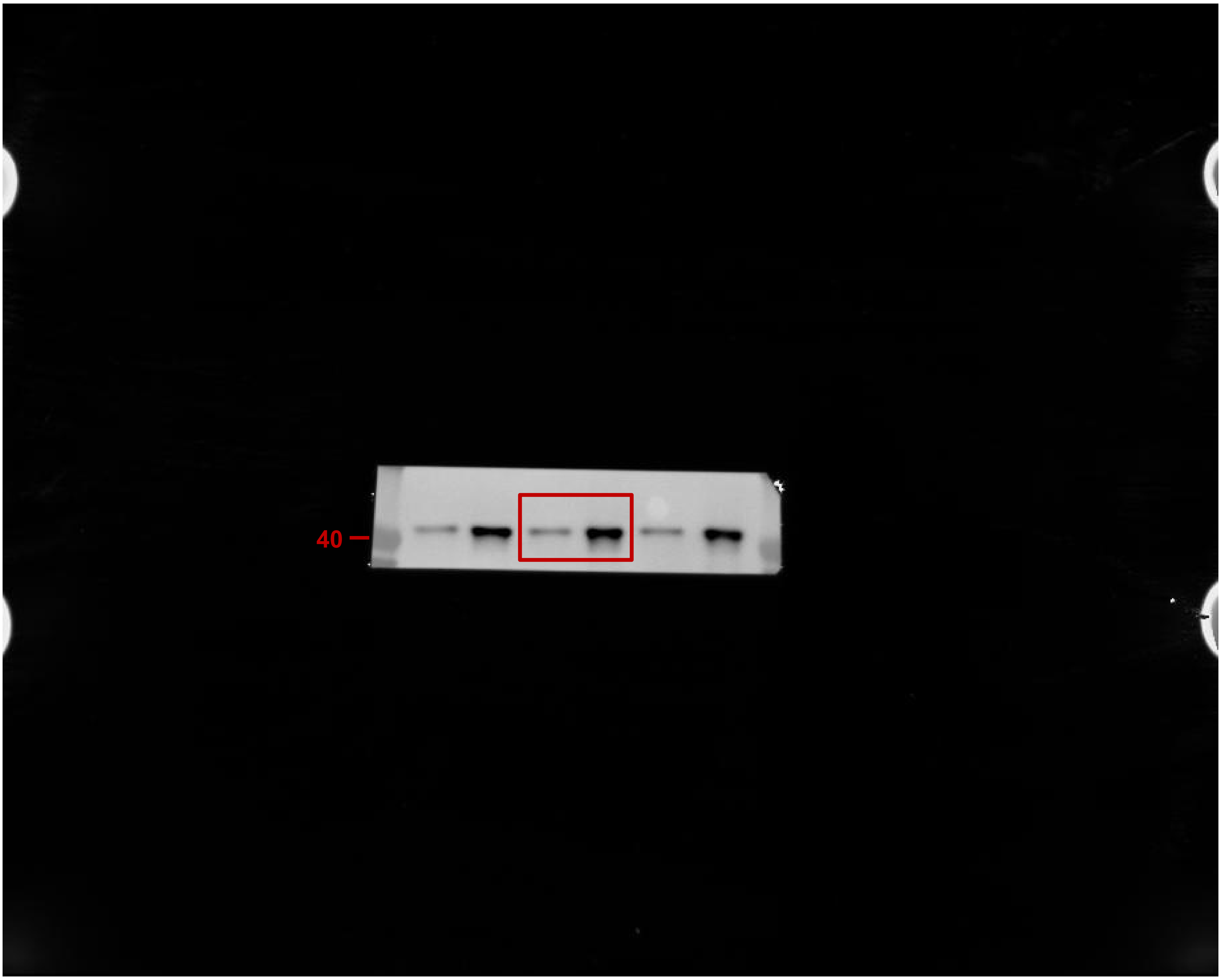

Figure S5E ALDOA

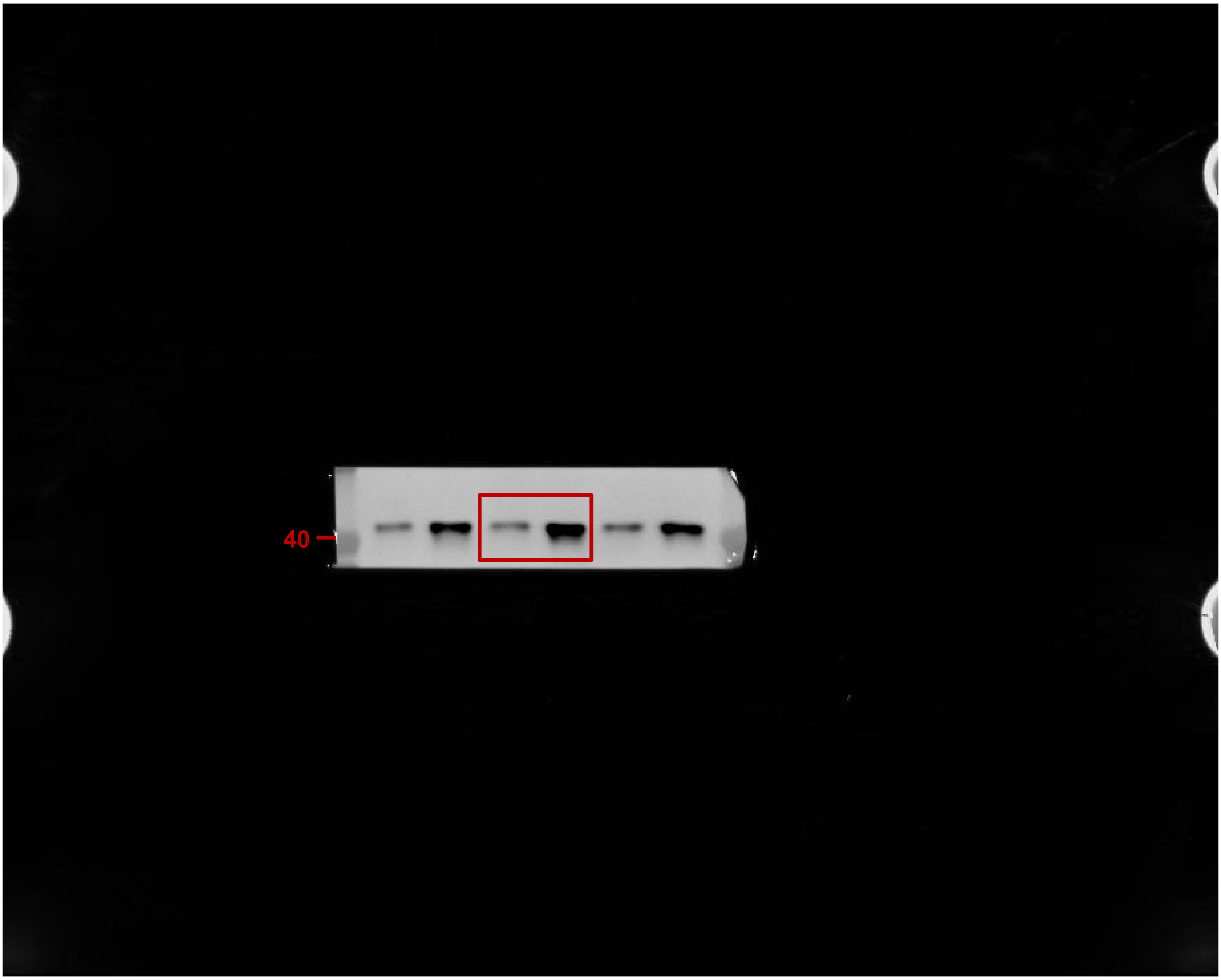

Figure S5E Tubulin

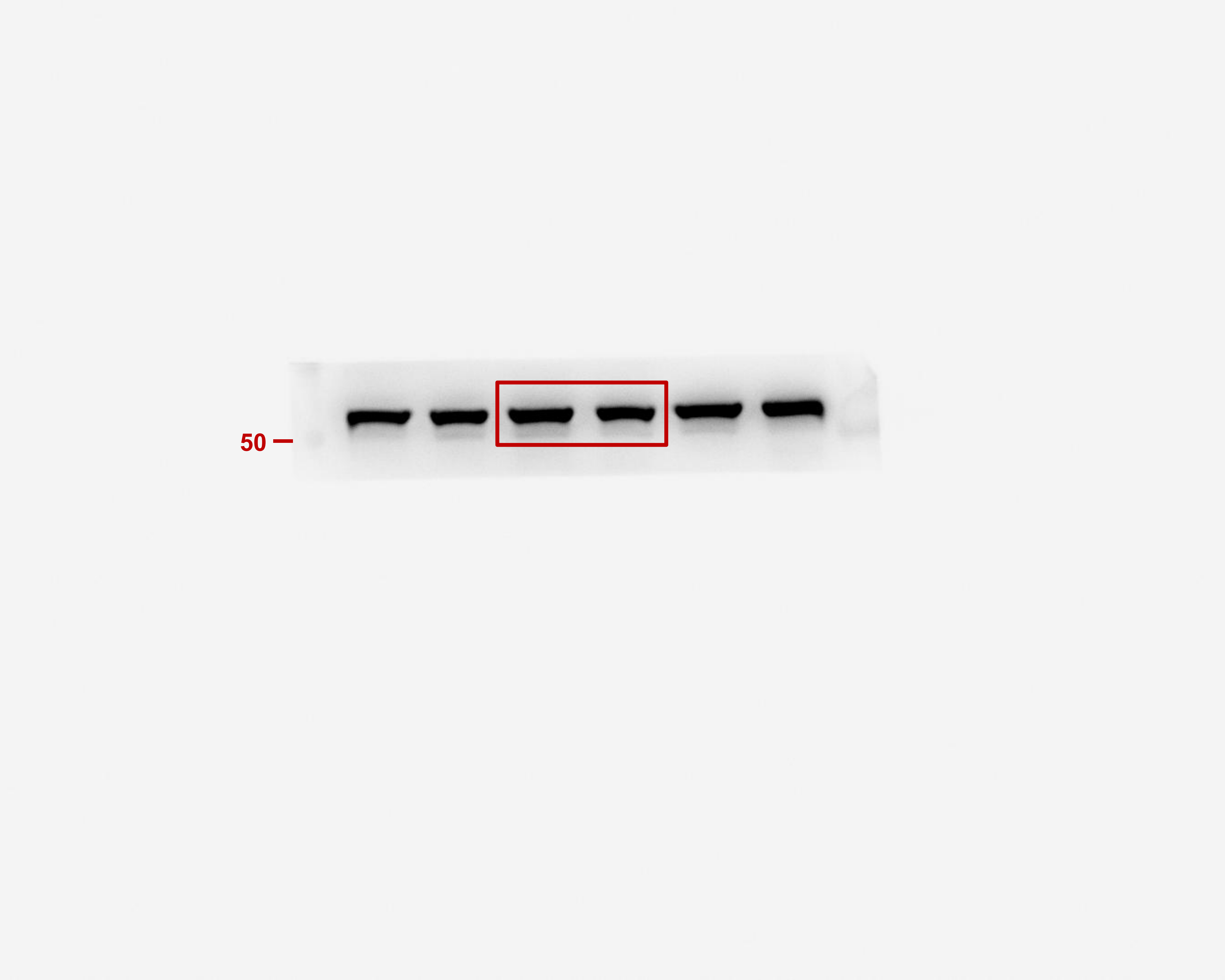

Figure S5E Tubulin

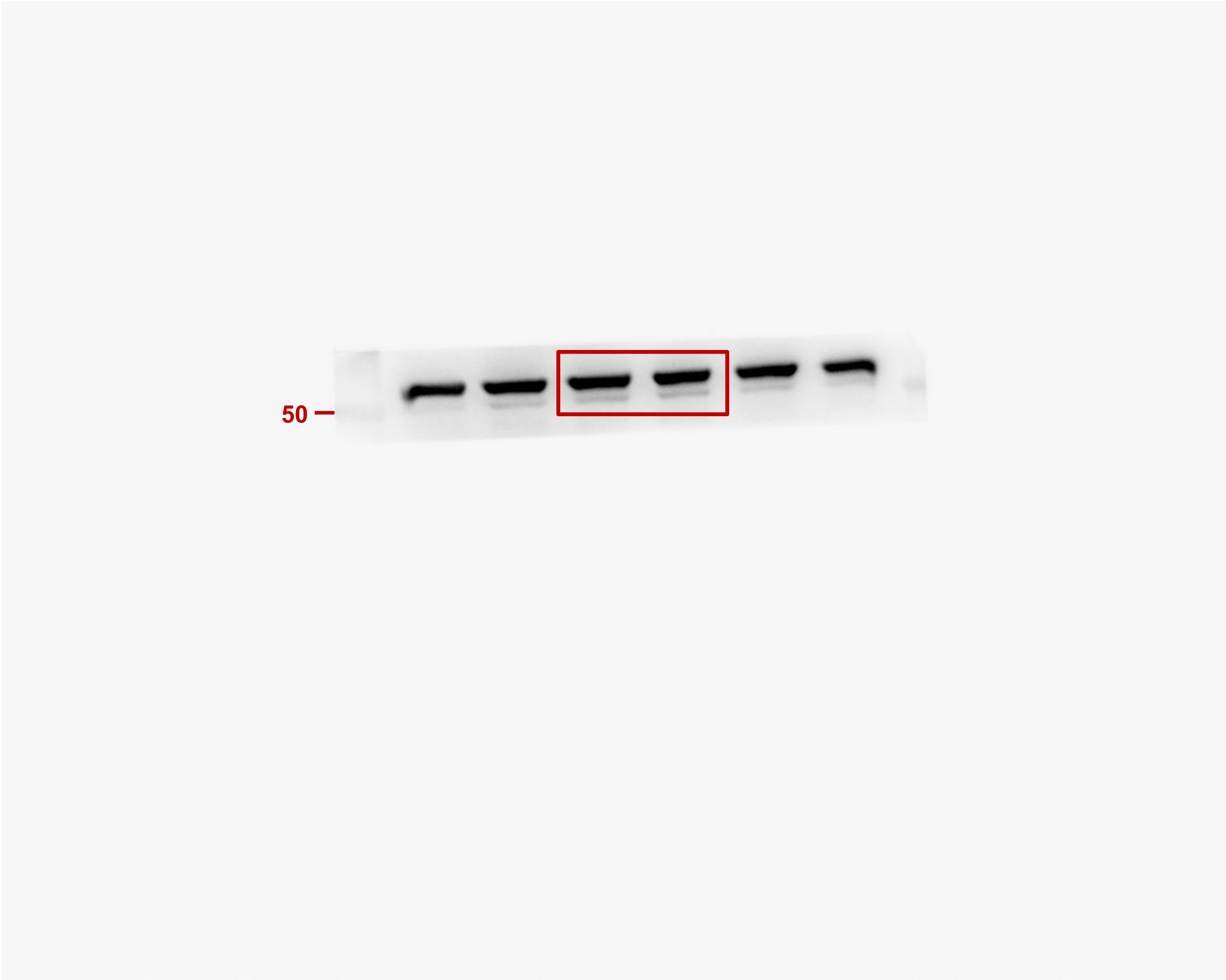

Figure S7 p-LATS1 HT29

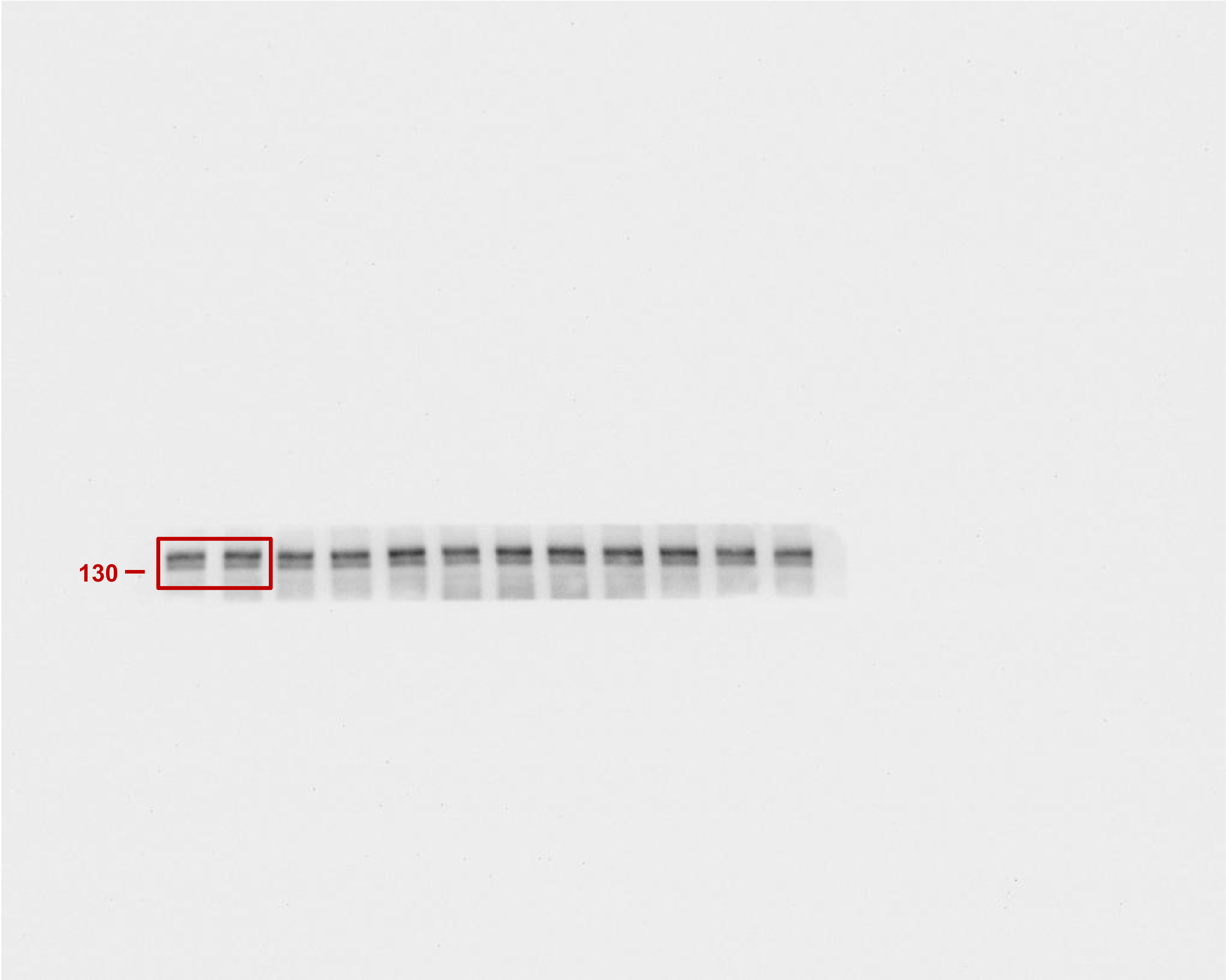

Figure S7 LATS1 HT29

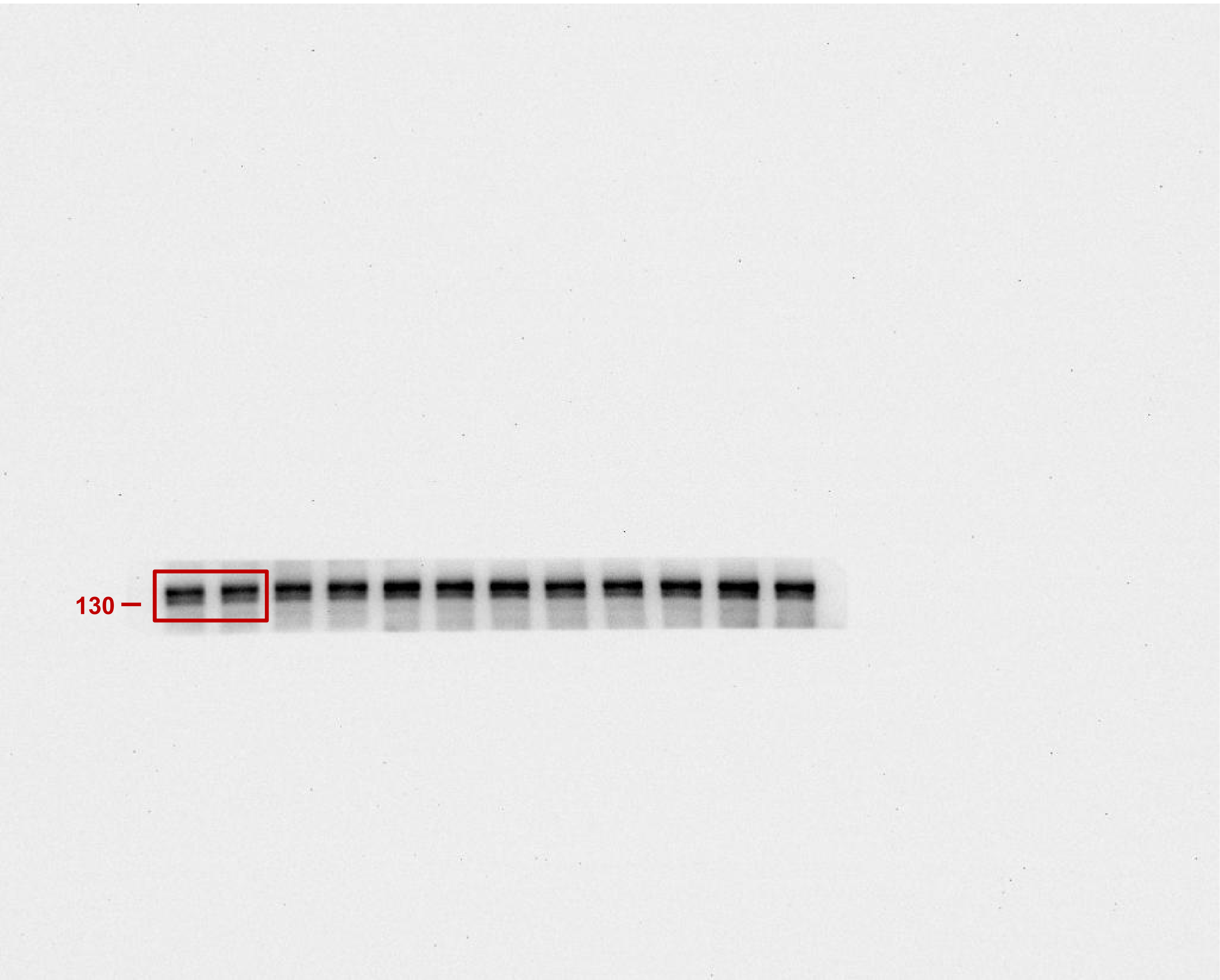

Figure S7 p-LATS1 SW480

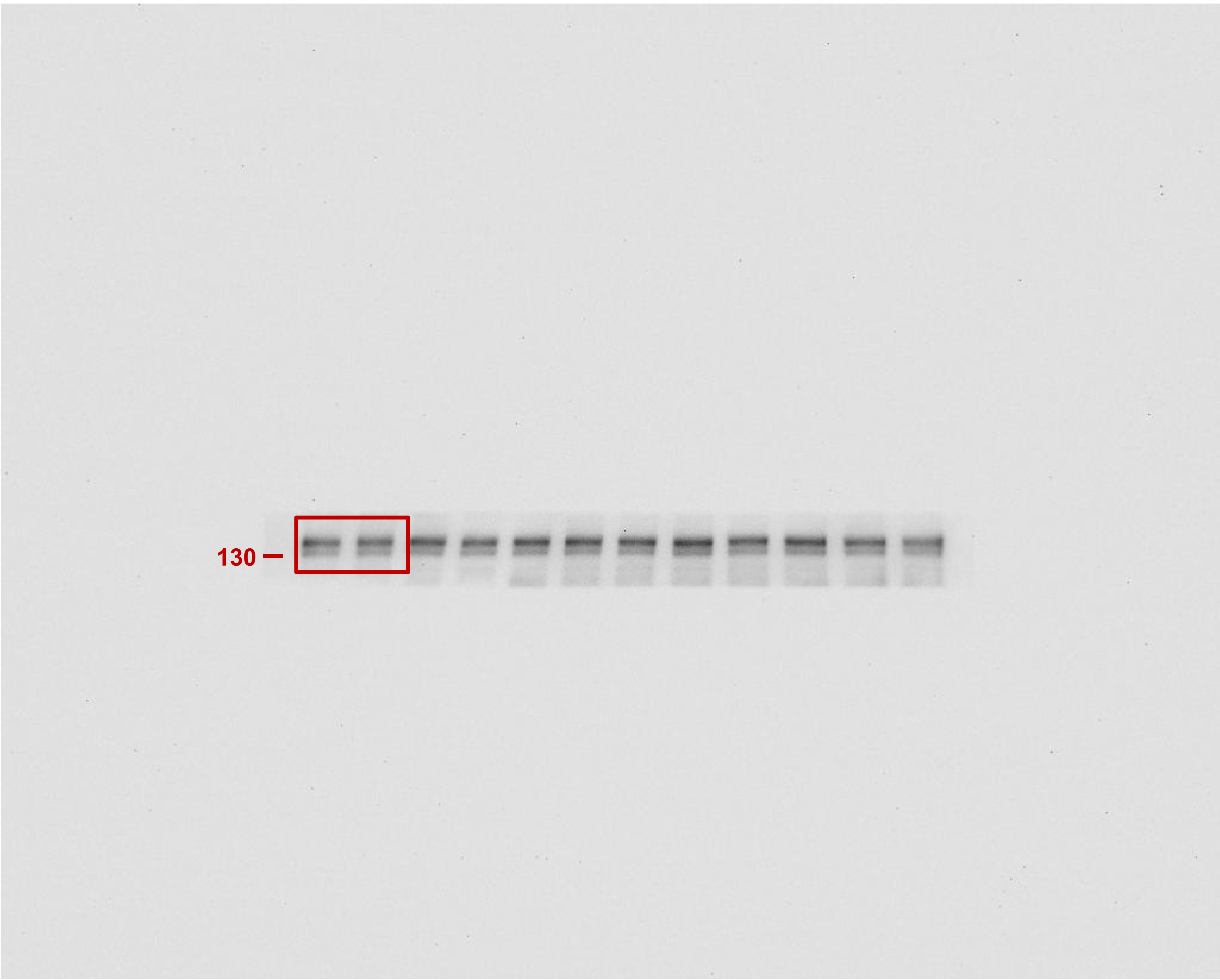

Figure S7 LATS1 SW480

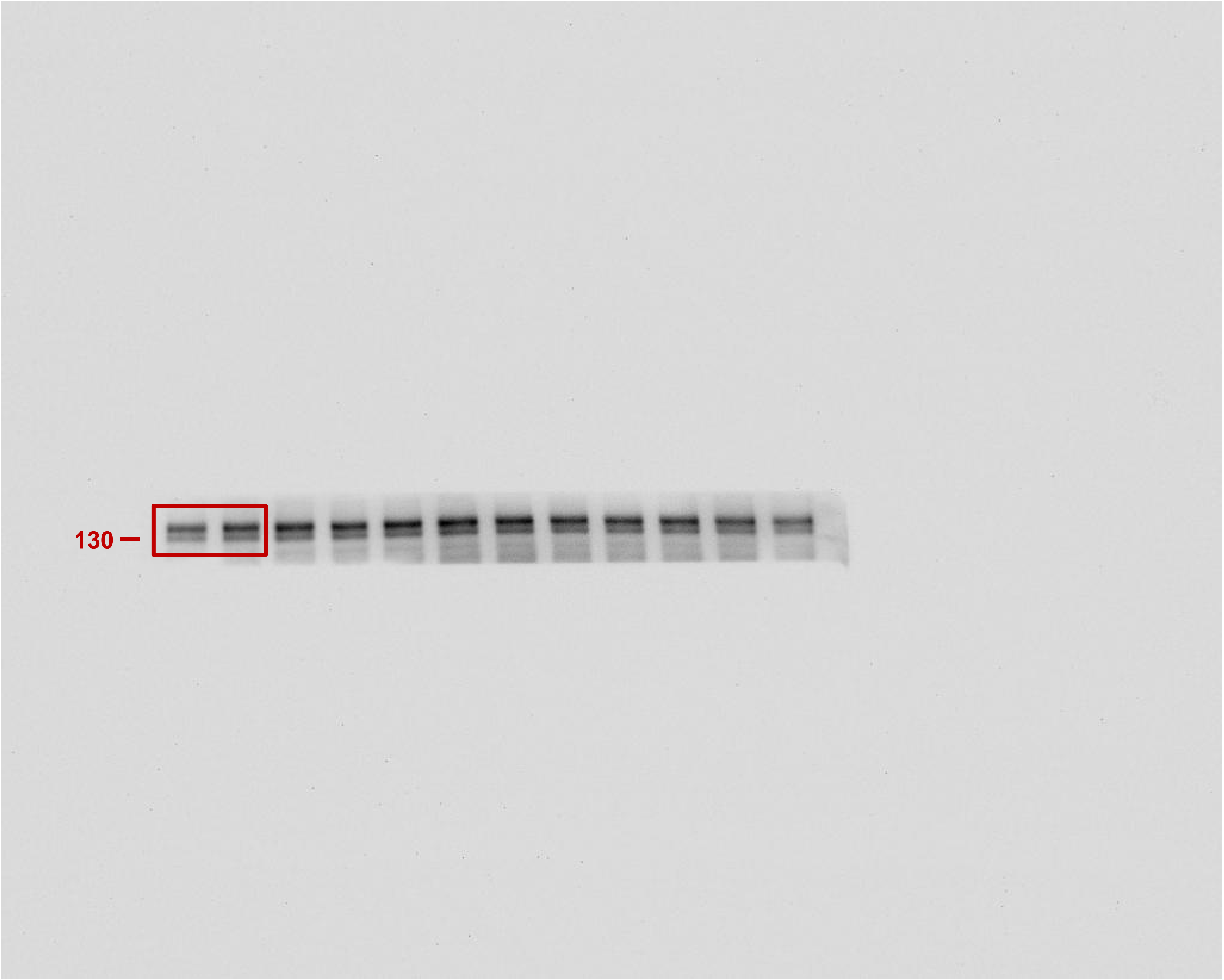

Figure S7 Tubulin HT29

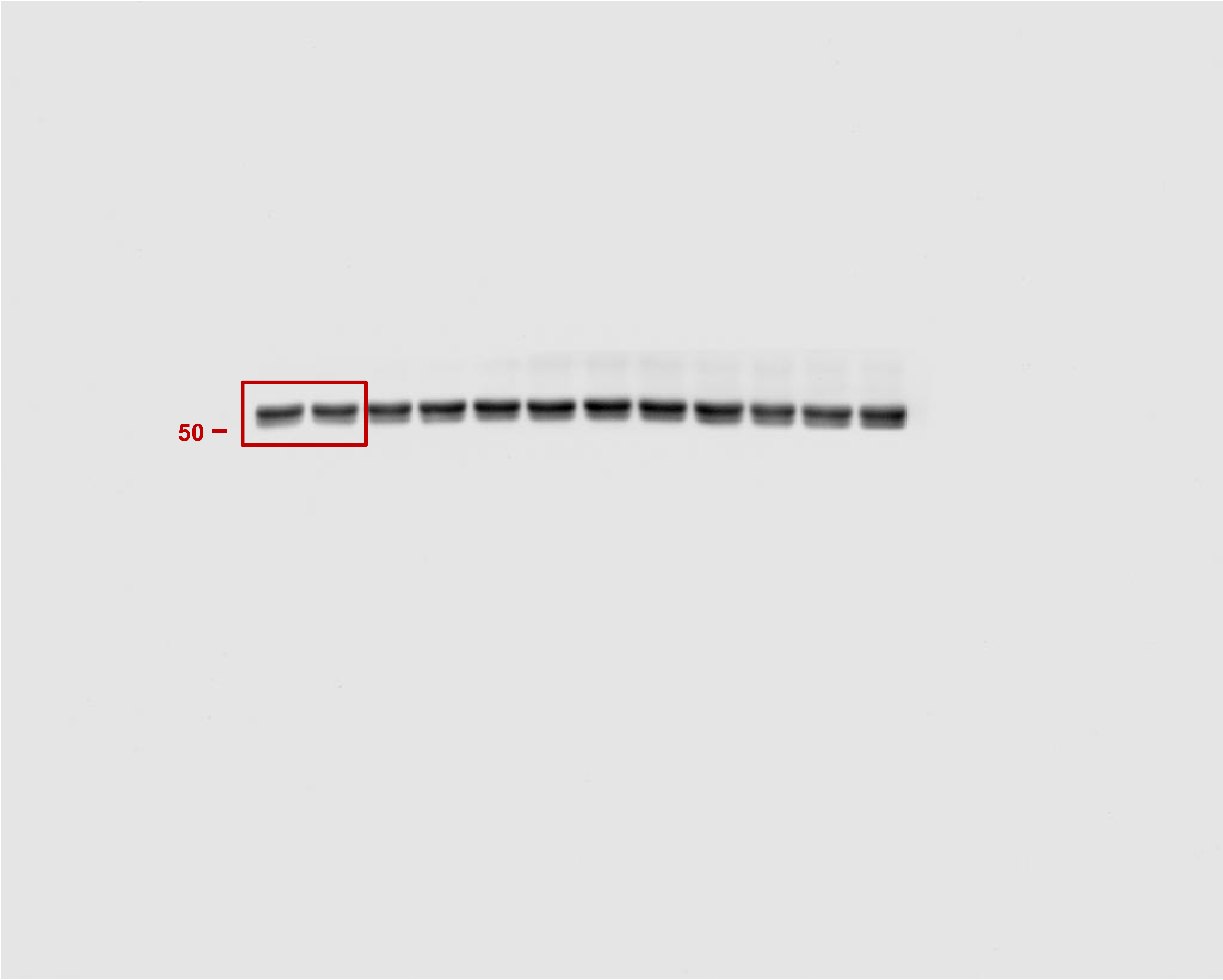

Figure S7 Tubulin SW480

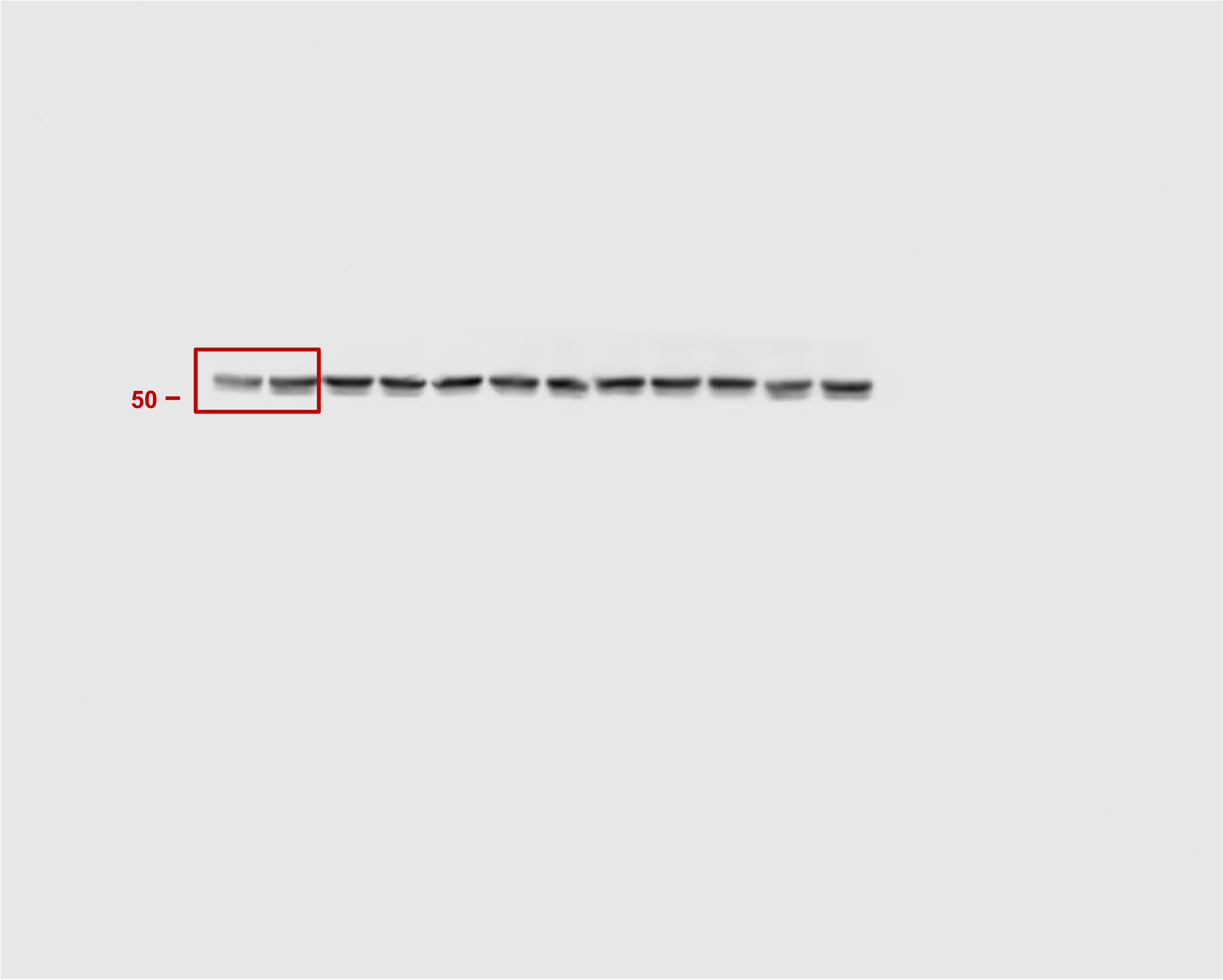

Figure S8A YAP

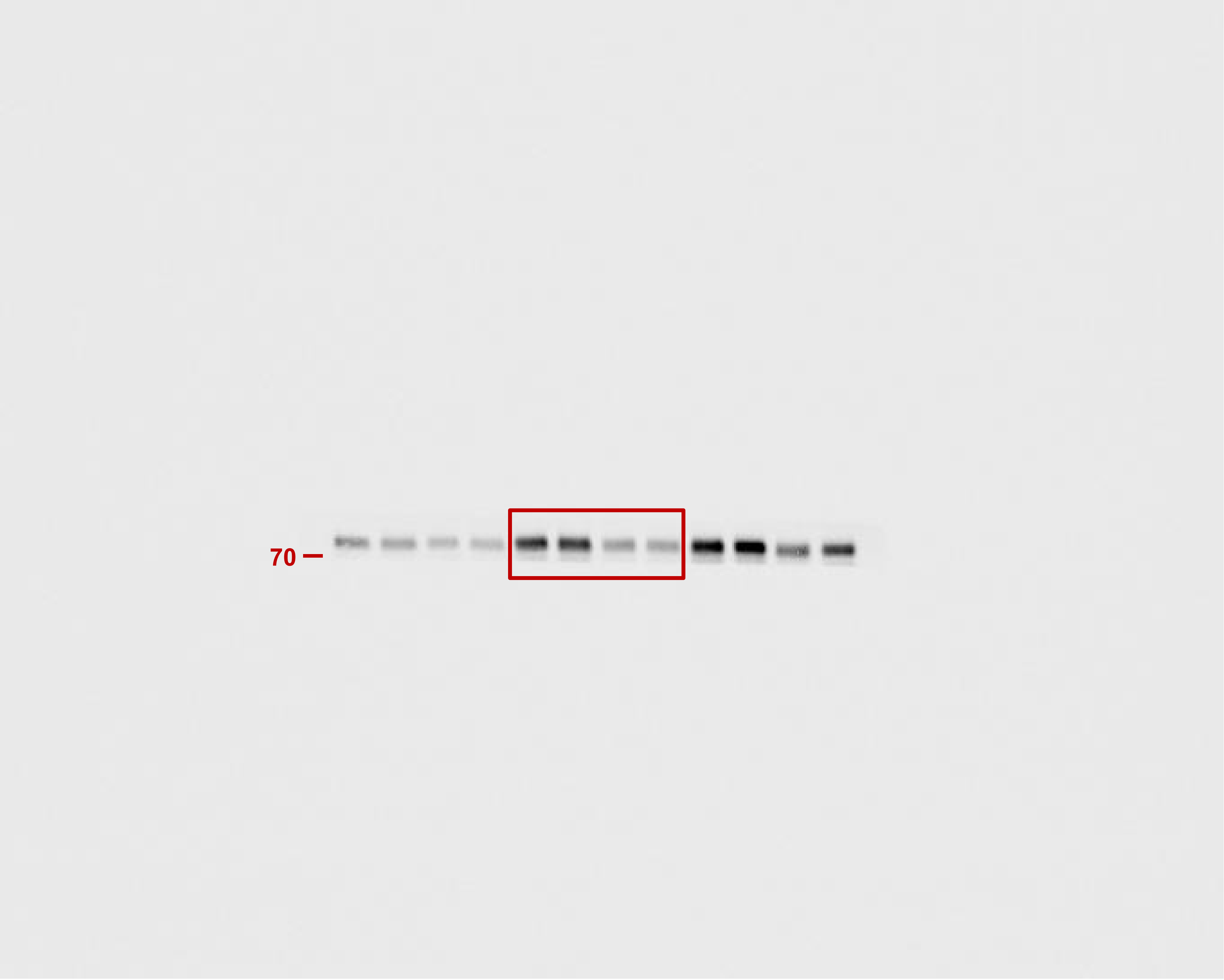

Figure S8A Tubulin

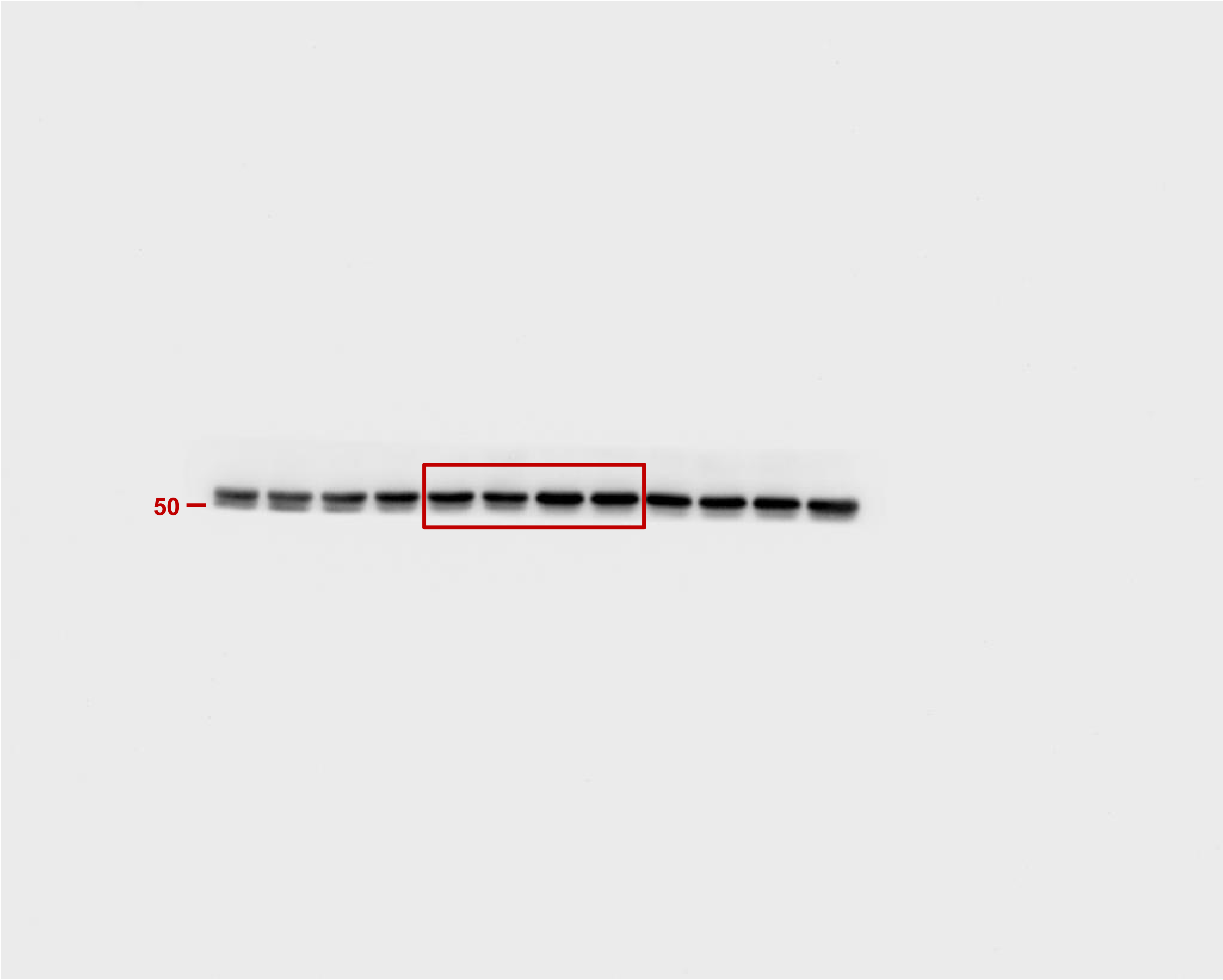

Figure S9 AMPK

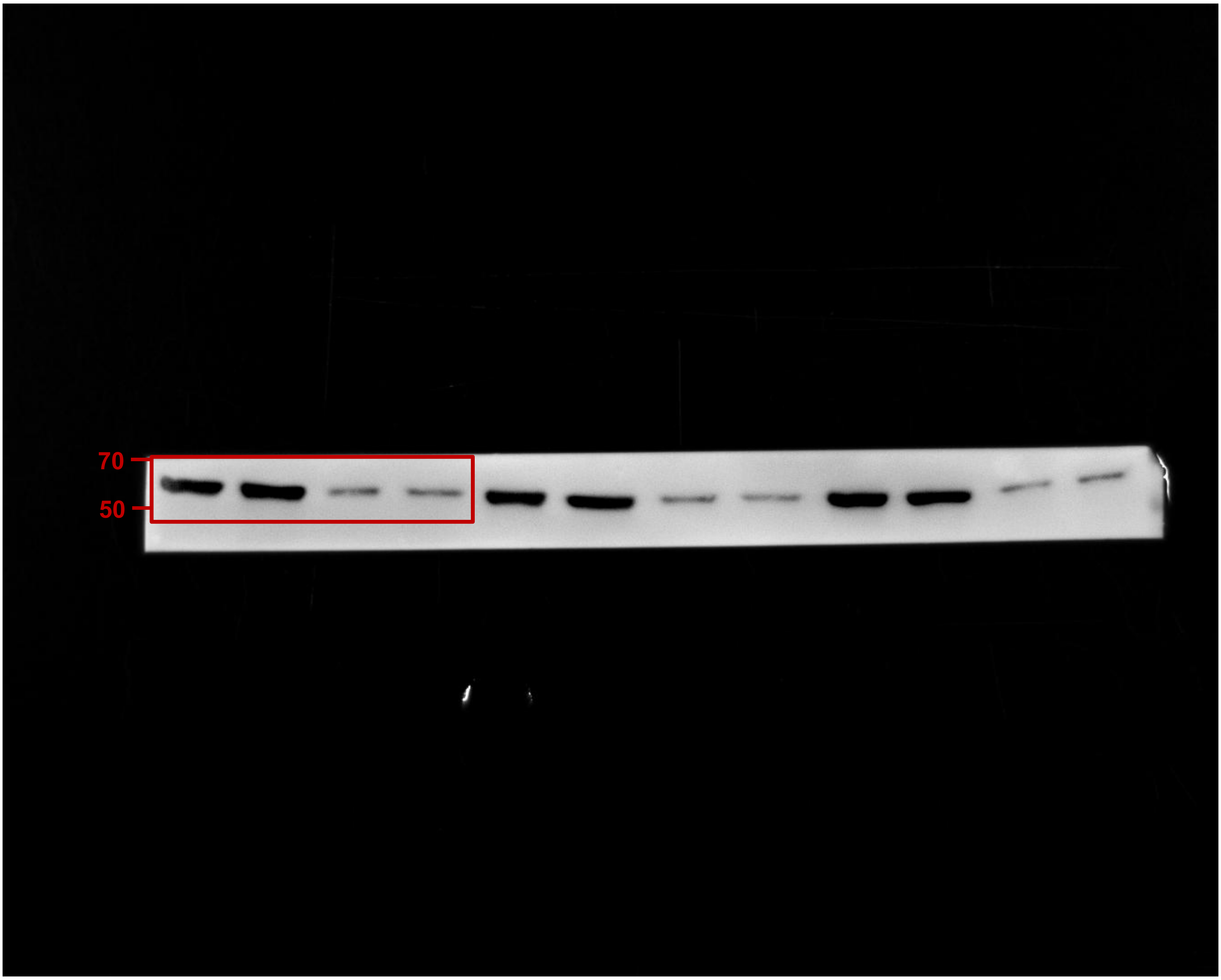

Figure S9 p-YAP

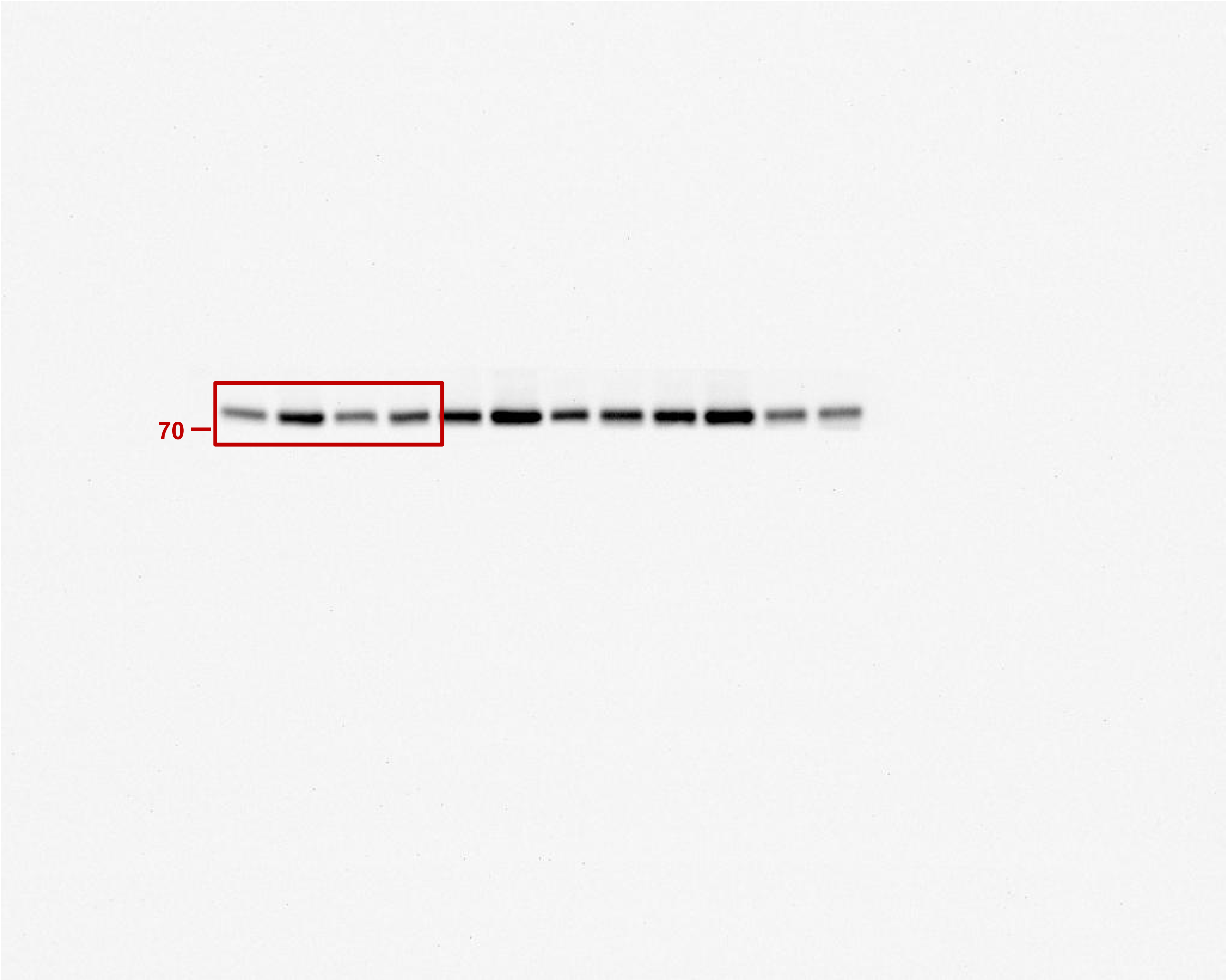

Figure S9 YAP

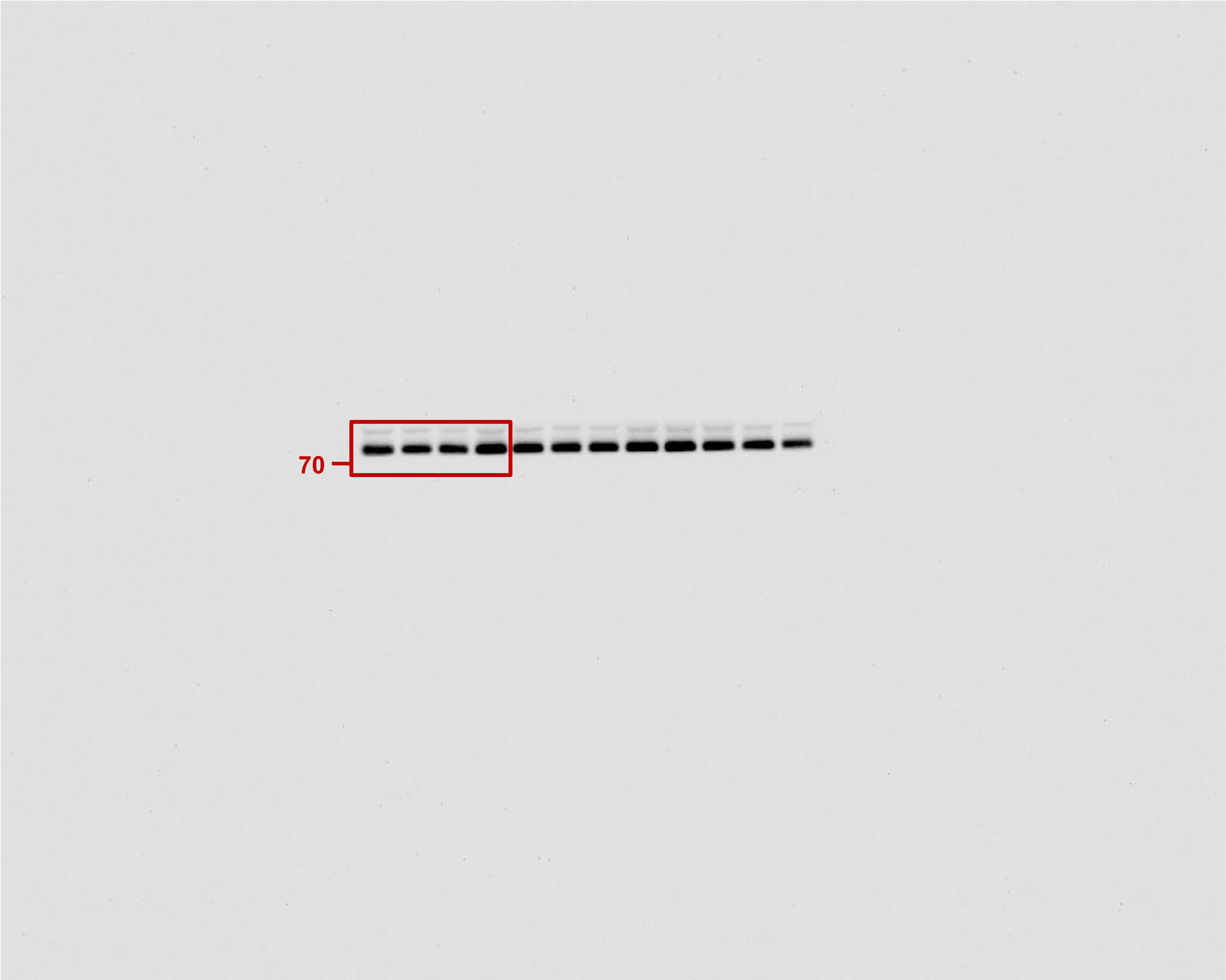

Figure S9 Tubulin

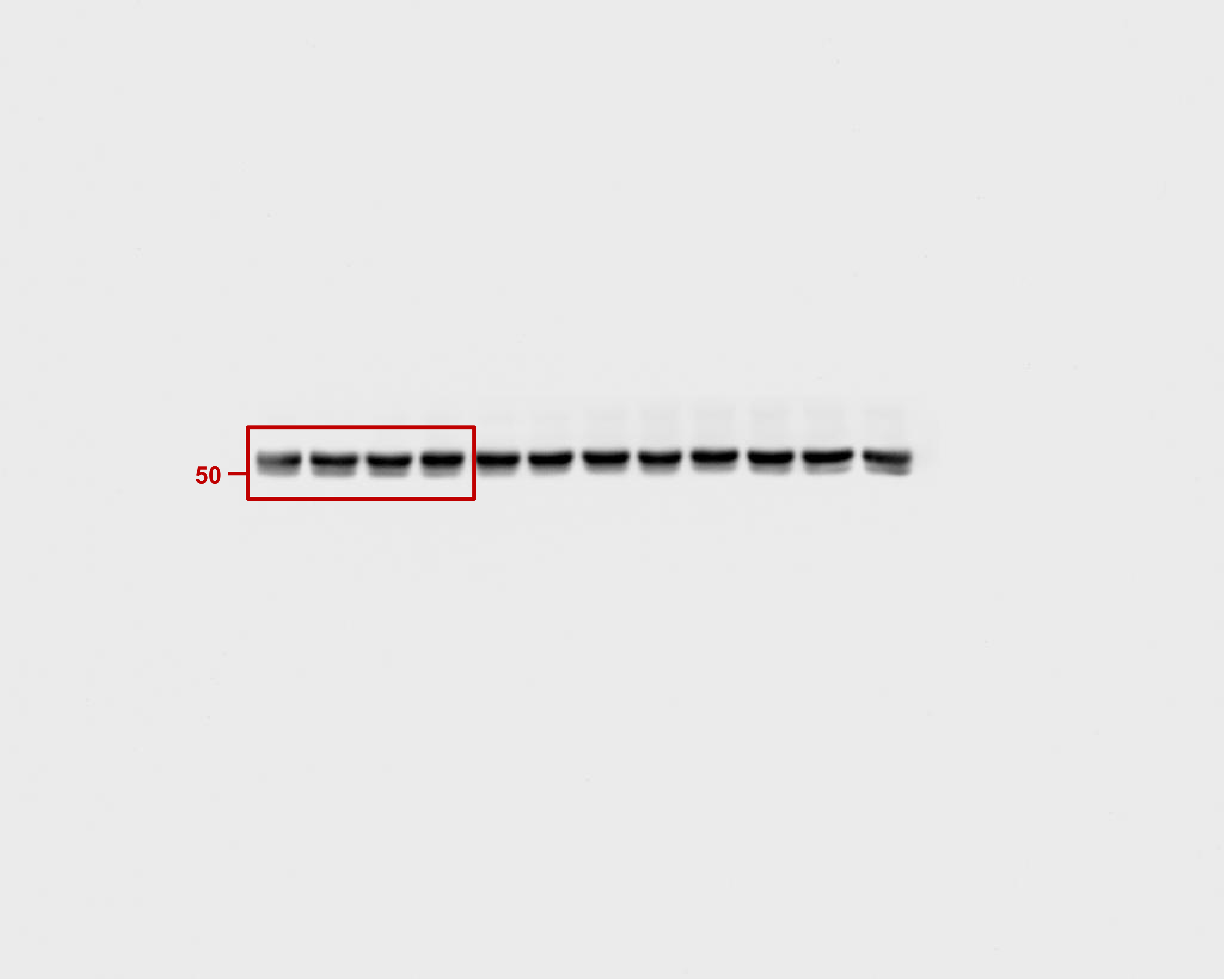

Supplement: Supplementary file 2 — Original Western Blots [file 41420_2024_2249_MOESM2_ESM.pdf]
